# Pnictogen-based vanadacyclobutadiene complexes†

**DOI:** 10.1039/d4sc05884d

**Published:** 2024-11-01

**Authors:** Mehrafshan G. Jafari, John B. Russell, Hwan Myung, Seongyeon Kwon, Patrick J. Carroll, Michael R. Gau, Mu-Hyun Baik, Daniel J. Mindiola

**Affiliations:** a Department of Chemistry, University of Pennsylvania Philadelphia Pennsylvania 19104 USA mindiola@sas.upenn.edu; b Department of Chemistry, Korea Advanced Institute of Science and Technology (KAIST) Daejeon 34141 Republic of Korea; c Center for Catalytic Hydrocarbon Functionalizations, Institute for Basic Science (IBS) Daejeon 34141 Republic of Korea

## Abstract

The reactivity of the V≡C^*t*^Bu multiple bonds in the complex (dBDI)V≡C^*t*^Bu(OEt_2_) (C) (dBDI^2−^ = ArNC(CH_3_)CHC(CH_2_)NAr, Ar = 2,6-^*i*^Pr_2_C_6_H_3_) with unsaturated substrates such as N≡CR (R = Ad or Ph) and P≡CAd leads to the formation of rare 3d transition metal compounds featuring α-aza-vanadacyclobutadiene, (dBDI)V(κ^2^-***C***,***N***-^*t*^Bu**C**C(R)N) (R = Ad, 1; R = Ph, 2) and β-phospha-vanadacyclobutadiene moieties, (dBDI)V(κ^2^-***C***,***C***-^*t*^Bu**C**P**C**Ad) (3). Complexes 1–3 are characterized using multinuclear and multidimensional NMR spectroscopy, including the preparation of the 50% ^15^N-enriched isotopologue (dBDI)V(κ^2^-***C***,***N***-^*t*^BuCC(Ad)^15^N) (1-^15^N). Solid-state structural analysis is used to determine the dominant resonance structures of these unique pnictogen-based vanadacyclobutadienes. A systematic comparison with the known vanadacyclobutadiene (dBDI)V(κ^2^-***C***,***C***-^*t*^Bu**C**C(H)**C**^*t*^Bu) (4) is also presented. Theoretical investigations into the electronic structure of 2–4 highlight the crucial role of unique V–heteroatom interactions in stabilizing the vanadacyclobutadienes and identify the most dominant resonance structures.

## Abstract 2

The reactivity of a [V^V^] alkylidyne with unsaturated substrates such as N≡CR (R = Ad or Ph) and P≡CAd leads to the formation of rare transition metal complexes featuring an α-aza-vanadacyclobutadiene and a β-phospha-vanadacyclobutadiene moieties.

## Graphical abstract

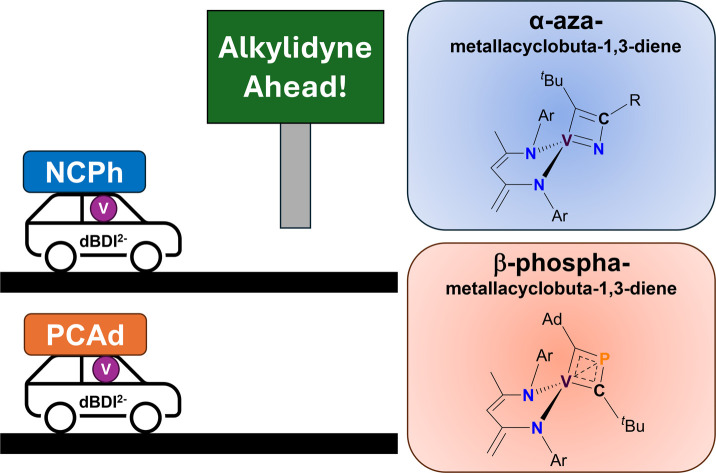

## Introduction

**Fig. 1:**
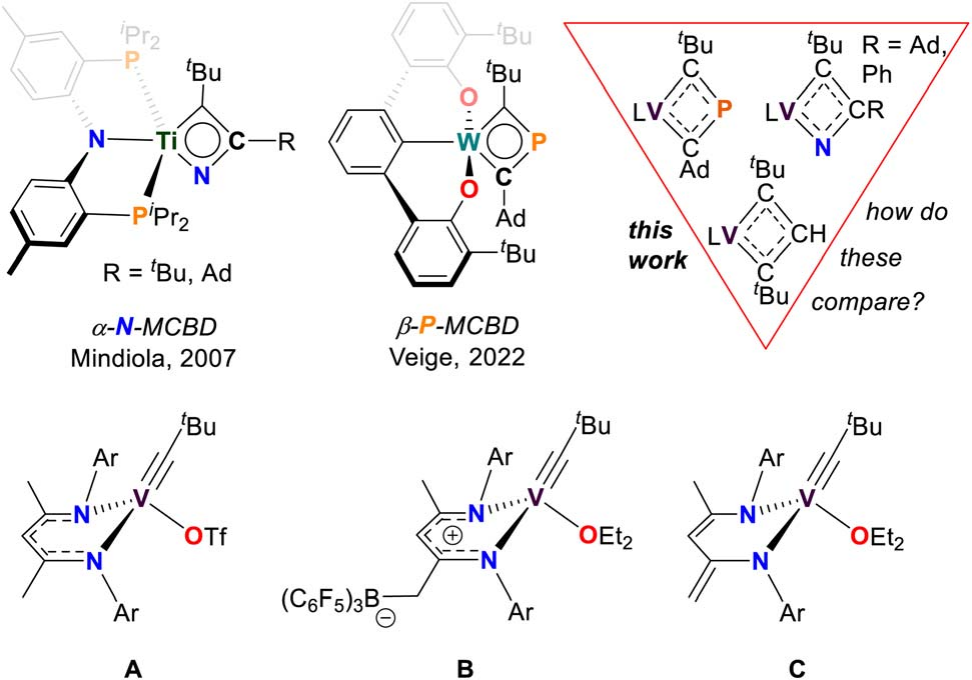
Top: Isolable pnictogen-based metallacyclobutadienes (MCBD) complexes and the vanadacyclobutadiene (VCBD) species reported in this work (L = dBDI^2−^). Bottom: Selected [V^V^] alkylidyne complexes, with complex C being the focus of the present study.

Metallacyclobutadienes (MCBDs) are proposed intermediates, and in some cases, isolable species in alkylidyne-alkyne cross-metathesis reactions.^1–22^ However, when a metal alkylidyne (M≡CR), reacts with a nitrile (N≡CR), or a phosphaalkyne (P≡CR), cross-metathesis proceeds *via* the formation of pnictogen-based heterometallacyclobutadiene. In the case of N≡CR cycloaddition across the M≡C bond, the reaction yields an α-aza-metallacyclobutadiene (α-N-MCBD, top left of Fig. 1).^23,24^ Such species are proposed as intermediates not only in cross-metathesis of a M≡CR with a N≡CR, but also in the exchange of a nitride M≡N with the corresponding alkyne (RC≡CR).^25,26^ To date, the isolation of an α-N-MCBD scaffold has been reported only by us with titanium, in the form of the complex (PNP)Ti(κ^2^-***C***,***N***-^*t*^Bu**C**C(R)**N**)^23^ (R = ^*t*^Bu, Ad; Ad = 1-adamantyl; PNP = N[2-P^*i*^Pr_2_-4-methylphenyl]_2_^−^). Although the latter species could not be structurally confirmed *via* single crystal X-ray diffraction analysis (scXRD), a combination of ^15^N isotopic labelling, NMR spectroscopy, and computational studies, strongly supported the existence of a planar, four-membered ring.

Given the isolobal relationship between C^−^ and P (or RC and P), one would anticipate P≡CR, to undergo metathesis reactions with M≡CR in a manner like alkynes. In this context, Hill and co-workers have explored the reactivity of M≡CR with P≡CR,^27,28^ but it was only recently that Veige and co-workers successfully isolated a monometallic β-phospha-metallacyclobutadiene (β-P-MCBD) scaffold.^29^ In their study, a β-P-MCBD scaffold (middle top of Fig. 1) in complex (^*t*Bu^OCO)W(κ^2^-***C***,***C***-^*t*^Bu**C**P**C**Ad) (^*t*Bu^OCO^3−^ = ipso-C_6_H_3_[2,6-(C_6_H_3_-*o*-^*t*^Bu)_2_]) was formed *via* insertion of a C^*t*^Bu fragment into a side-on bound, 1-adamantyl phosphaethyne, P≡CAd ligand. Due to the electronegativity difference between N (3.04) and P (2.19) on the Pauling scale, the regioselectivity in the cycloaddition step of the pnictogen based alkyne, Pn≡CR (Pn = pnictogen) across the M≡C bond was reversed. Additionally, Hard–Soft Acid–Base (HSAB) considerations could also be applied to these differences suggesting that the hard nitrogen atom would preferentially bind to the hard [Ti^IV^] nucleus while the soft phosphorus atom would not want to interact with the hard [W^VI^] nucleus.

Heteroatom containing MCBDs are seldom reported, and their chemistry remains largely unexplored, with a few notable exceptions shown in Fig. 1.^29–39^ In the context of 3d transition metals, alkylidyne cross-metathesis reactions involving vanadium are virtually unknown, since M≡CR motifs have been rarely documented with this class of metal ions.^23,40–56^ There are only a few examples of stable vanadium Schrock-like carbyne complexes known.^41,47,57^ Notably, vanadium alkylidynes (V≡CR) (A–C in Fig. 1), are catalysts for the formation of cyclic polyphenylacetylene.^58^ It was suggested that the initiation step of the polymerization involved a [2+2] cycloaddition of the V≡CR fragment and a phenylacetylene (PhC≡CH) to form a vanadacyclobutadiene (VCBD). This was further corroborated through our isolation of the VCBD (dBDI)V(κ^2^-***C***,***C***-^*t*^Bu**C**C(H)**C**^*t*^Bu)^58^ (4), (dBDI^2−^ = ArNC(CH_3_)CHC(CH_2_)NAr, Ar = 2,6-^*i*^Pr_2_C_6_H_3_) (top right of Fig. 1). Noting that the barrier for metathesis was quite low for complex C (21 kcal mol^−1^) and given that no structurally characterized examples of an α-N-MCBD exist, and only a single structural example of a β-P-MCBD is known, we sought to explore this rare ligand class with the V≡CR motif and compare their structural topology.

In this study, we report VCBD complexes that incorporate pnictogens (Pn) and compare them to the known carbon-only vanadacyclobutadiene (4), considering the isolobal relationship between CH and P/N. Although these scaffolds are generally represented as fully delocalized, substantial differences in the electronic structure are expected due to the disparity in electronegativities between N (3.04), C (2.55), and P (2.19). Indeed, cycloaddition intermediates between M≡C motifs and Pn≡C bonds of heteroalkynes have rarely been isolated, and with the increasing interest in the chemistry of four-membered hetero-metallacycles,^29,33,59–68^ we decided to investigate the reactivity of V≡CR with Pn≡CR. Herein, we present the successful isolation of the first α-aza-vanadacyclobutadiene (α-N-VCBD) and a rarely reported β-phospha-vanadacyclobutadiene (β-P-VCBD) complexes, complete with single crystal X-ray diffraction (scXRD) studies. We performed a systematic comparison between these complexes, focusing on their spectroscopic features and structural metrics to evaluate the influence of a heteroatom substitution. Additionally, density functional theory (DFT) calculations were employed to assess the impact of the heteroatoms on the electronic and resonance structures of these complexes.

## Results and discussion

Among the vanadium alkylidynes A–C, complex C (Fig. 1) stands out as the most suitable reagent for various reactions, due to its stability against intramolecular degradation and its enhanced reactivity, which is attributed to the lability of the diethyl ether (Et_2_O) ligand. Consequently, we directed our efforts toward investigating the reactivity of this complex in cycloaddition reactions with unsaturated substrates, such as Pn≡CR (Pn = N, R = Ad, Ph; Pn = P, R = Ad), to determine whether they form stable Pn-based VCBDs. These substrates were selected to probe the nucleophilic nature of the alkylidyne carbon and to examine the differences in bond formation between the more electronegative N and the more electropositive P. We envisioned that gaining structural insights into these rare heteroatom-substituted MCBD fragments, by exchanging isolobal moieties CH, N, and P, would allow us to compare their electronic properties.

### Synthesis and characterization of the α-N-VCBD scaffold

**Scheme 1:**
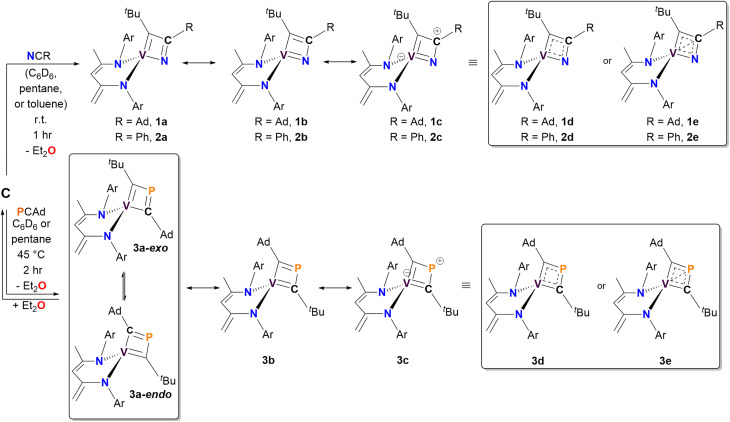
Synthesis of complexes 1–3*via* the [2+2]-cycloaddition of the vanadium alkylidyne C with pnictogen-containing alkynes Pn≡CR (Pn = N, R = Ad or Ph; Pn = P, R = Ad). Potential resonance structures and charge delocalized structures for each complex are also illustrated.

We anticipated that the higher electronegativity of nitrogen (3.04) would favor the formation of a C–C bond between the alkylidyne carbon and the nitrile carbon atoms.^69,70^ As expected, treating C with N≡CR (R = Ad, Ph) in pentane, deuterated benzene (C_6_D_6_), or toluene (for 2) at room temperature for one hour resulted in the formation of α-N-VCBD complexes (dBDI)V(κ^2^-***C***,***N***-^*t*^Bu**C**C(R)**N**) (R = Ad, 1, 49% yield; Ph, 2, 89% yield), as illustrated in the top portion of Scheme 1. For N≡CAd, the reaction yielded a brown-colored complex, 1. However, the formation of 1 required an excess amount of nitrile and dilute reaction conditions to attain full conversion. Repetitive evacuation of the side product, Et_2_O, over the course of the reaction also promoted the formation of 1. In contrast, the reaction of C with one equivalent of N≡CPh proceeded almost immediately, forming purple-red complex 2 in nearly quantitative yields (89% isolated).

**Fig. 2:**
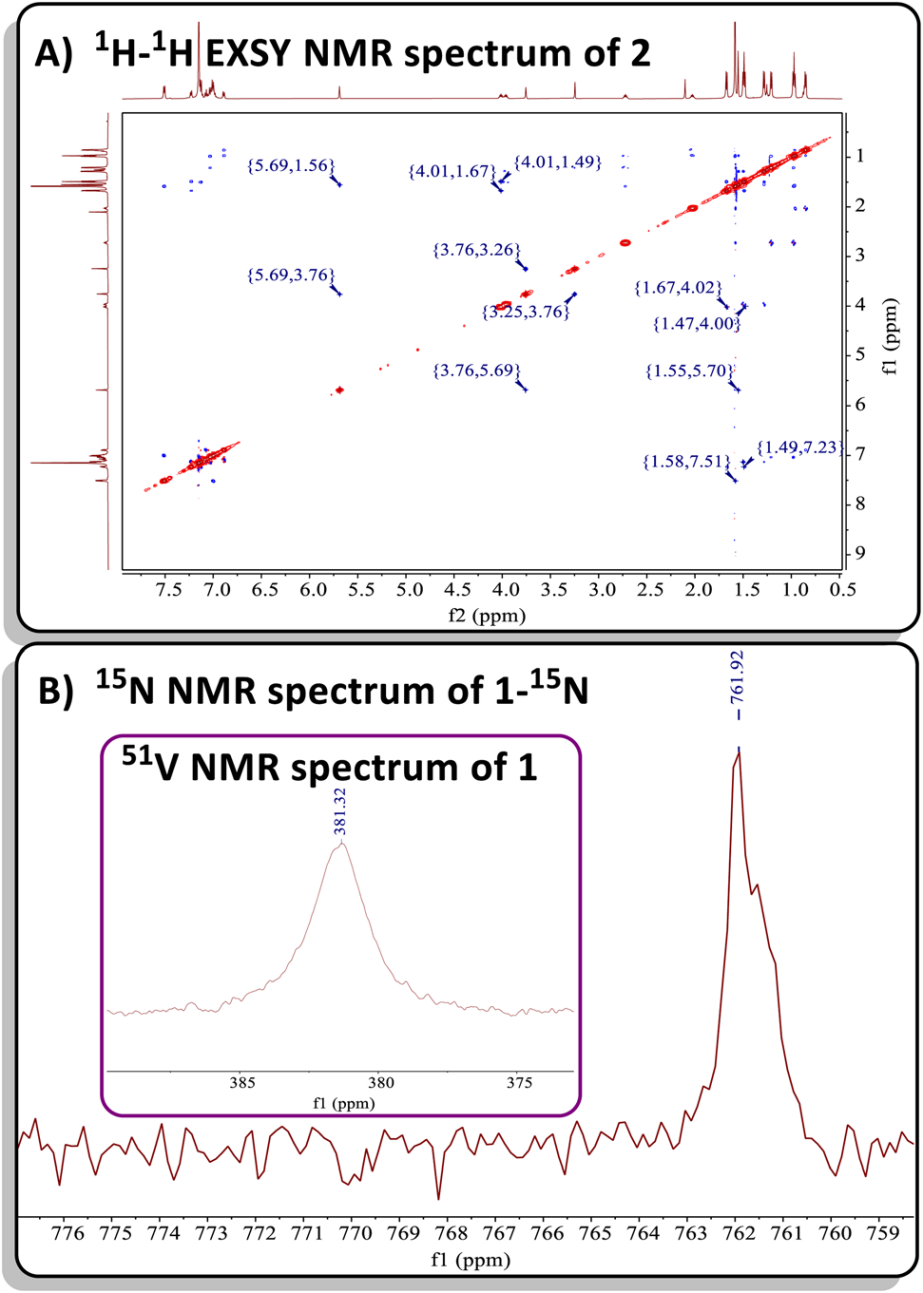
(A) ^1^H–^1^H EXSY NMR spectrum of 2 showcasing its fluxionality at room temperature. (B) ^15^N NMR spectrum of 50% ^15^N enriched complex 1-^15^N. Inset: ^51^V NMR spectrum of 1.

Both complexes 1 and 2 exhibit characteristic resonances in the ^1^H NMR spectrum for the methylene moiety of the bis-anilido ligand (dBDI^2−^), showing a virtual pair of doublets at 3.76 and 3.29 ppm for 1 (Fig. S2†) and at 3.75 and 3.25 ppm for 2 (Fig. S9†). The resulting ^1^H–^13^C HSQC experiment on complex 2 (Fig. S13†) revealed that two inequivalent proton resonances correspond to a single carbon resonance at 88.65 ppm. The corresponding ^13^C{^1^H} DEPT-135 spectrum of 2 (Fig. S11†) revealed the carbon resonance at 88.65 ppm has sp^2^ like character, leading us to assign the methylene (C*H*_2_) fragment as this resonance. Like 1, the ^1^H and ^13^C{^1^H} NMR spectra of 2 reveal extensive overlap of resonances in the ^1^H NMR spectrum, indicating the presence of structurally similar but magnetically distinct compounds. The NMR spectral data for the less sterically hindered complex 2 are less complicated as only a single isomer is present, allowing for a complete assignment of resonances. For instance, the β-carbon in the four-membered ring of 2 was observed at 157.9 ppm in the ^13^C{^1^H} NMR spectrum (Fig. S10†), a shift further upfield than the previously reported titanium α-N-MCBD derivative (PNP)Ti(κ^2^-***C***,***N***-^*t*^Bu**C**C(Ad)**N**), which resonates at 240.5 ppm.^23^ Overall, the ^1^H and ^13^C{^1^H} NMR spectral data for complexes 1 and 2 are in accord with these compounds possessing *C*_1_ symmetry, as indicated by the presence of four inequivalent isopropyl methine resonances for the two aryl groups and two α-carbons for the chelating bis-anilido ligand. A ^1^H–^1^H EXSY NMR spectrum of 2 (Fig. 2A) also reveals the fluxionality of the molecule at room temperature, indicated by extensive off-diagonal couplings of protons, thus corroborating why two diastereomers are unobservable on the NMR time scale at room temperature. Moreover, the ^51^V NMR of 1 (Inset Fig. 2B) and 2 (Fig. S15†) show a single resonance feature at 381.32 ppm (Δ*ν*_1/2_ = 339.2 Hz) and 344.76 ppm (Δ*ν*_1/2_ = 276.0 Hz), respectively.

Due to the lipophilic nature of complex 1, numerous attempts to obtain single crystals were unsuccessful. Consequently, we turned to the ∼50% ^15^N enriched isotopologue, ^15^N≡CAd, prepared using the method reported by Johnson and co-workers.^71^ Previous reports have demonstrated that M–N multiple bonding with a planar, sp^2^-like nitrogen leads to a downfield shift in the ^15^N NMR resonance, compared to a more pyramidalized, sp^3^-like nitrogen involved in a plausible tetrahedrane MCBD structure.^23,72^ Treatment of 50% enriched ^15^N≡CAd with complex C, followed by an analogous workup, resulted in the isolation of the isotopologue (dBDI)V(κ^2^-***C***,***N***-^*t*^Bu**C**C(Ad)^**15**^**N**) (1-^15^N). Subsequent ^15^N NMR spectral analysis revealed a highly downfield resonance at ∼761 ppm, referenced to ^15^N≡CAd at 242 ppm *versus* NH_3_ (l) at 0 ppm at 27 °C (Fig. 2B).^4,69^ This value is consistent with the ^15^N chemical shift observed for the titanium α-N-MCBD complex, (PNP)Ti(κ^2^-***C***,***N***-^*t*^Bu**C**C(Ad)^**15**^**N**) (^15^N NMR: 672.6 ppm at 55 °C), suggesting that the nitrogen atom in 1-^15^N is likely sp^2^-hybridized, forming a planar, four-membered MCCN ring scaffold.^23^ In contrast to the niobium methylidyne complex (PNP)Nb≡CH(OAr), which undergoes cross-metathesis with [N≡CR] to produce (PNP)Nb≡N(OAr) and HC≡CR (R = ^*t*^Bu, Ad),^24^ the four-coordinate complexes 1 and 2 did not undergo [2+2]-cycloreversion and subsequent expulsion of the alkyne ^*t*^BuC≡CR (R = Ad, Ph). Computational studies have revealed that the dissociation of Et_2_O from C involves a transition state with an energy barrier of 26 kcal mol^−1^, while the transient alkylidyne species {(dBDI)V≡C^*t*^Bu} is approximately 21 kcal mol^−1^ higher in energy than its precursor.^58^ Therefore, the elimination of alkyne from 1 or 2 is improbable, as it would result in the formation of a highly reactive three-coordinate vanadium nitride {(dBDI)V≡N} fragment. Additionally, theoretical studies on all carbon based MCBD complexes have suggested that the retro [2+2] cycloaddition reaction is unfavourable for group 4 and 5 MCBDs.^73^ Notably, when an excess amount of ^15^N≡CAd was added to a C_6_D_6_ solution of complex 1, no formation of 1-^15^N was observed (monitored by ^15^N NMR spectroscopy over 24 hours), further indicating that the cycloreversion process is unfavourable under these conditions.

**Fig. 3:**
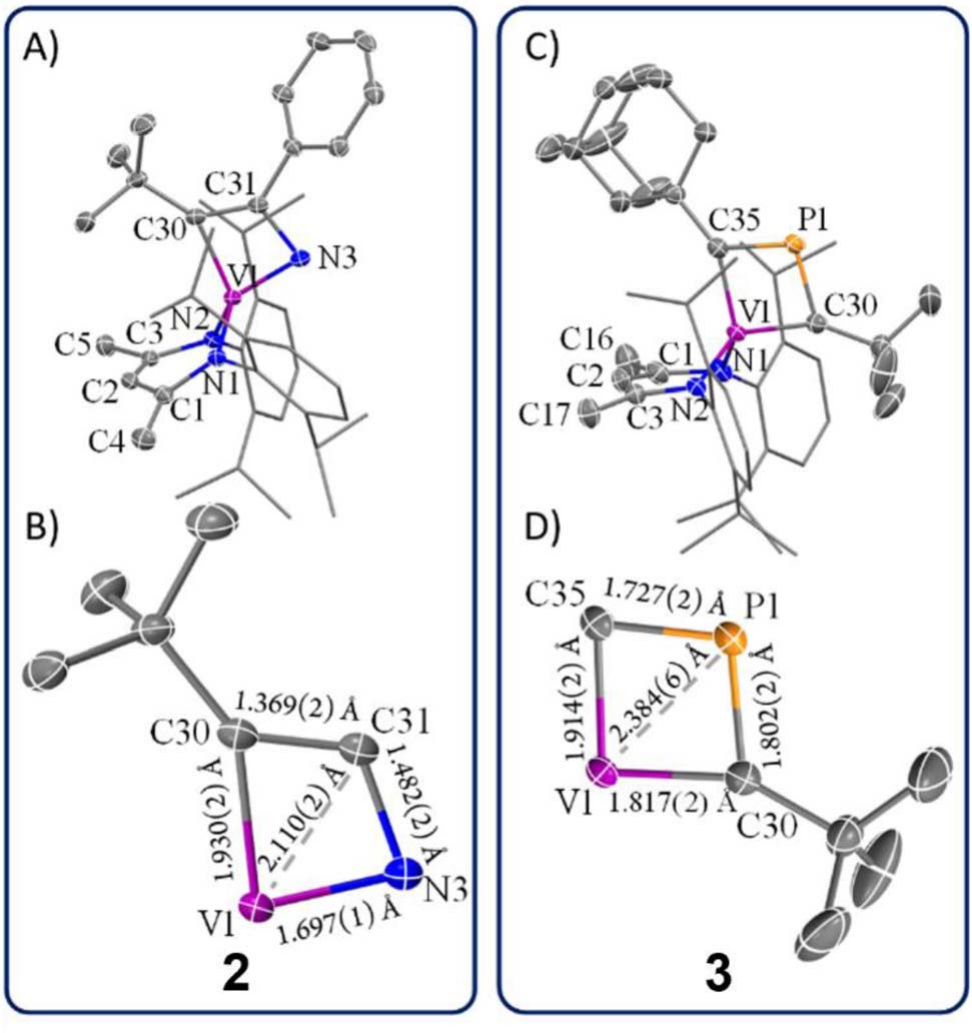
(A) Structural representation of 2 with thermal ellipsoids at 50% probability level and hydrogen atoms omitted for clarity. (B) A closer examination of bond distances in the α-N-VCBD motif in 2. (C) Structural representation of 3 with thermal ellipsoids at 50% probability level and hydrogen atoms omitted for clarity. (D) A closer examination of bond distances in the β-P-VCBD motif in 3.

Unlike complex 1, complex 2 can be crystallized as single crystals from a pentane/toluene vapor-diffused mixture cooled to −35 °C. scXRD analysis revealed that complex 2 crystallizes in the monoclinic and centrosymmetric space group *P*2(1)/*n*. Fig. 3A depicts the structure of 2, which manifests *C*_1_ symmetry, consistent with its solution-phase NMR spectra. The structure shows a short V1–N3 distance of 1.697(1) Å, indicating a strong V–N interaction, while the relatively long N3–C31 distance of 1.482(2) Å suggests a smaller contribution of the 2a resonance structure compared to 2b (Scheme 1). The V1–C30 distance of 1.930(2) Å is notably longer compared to typical V=C double bonds^40,74–91^ (1.7 Å to 1.9 Å), reported in the Cambridge structural database (CCDC), further supporting the dominance of resonance structure 2b over 2a. Additionally, the observed C30–C31 bond length of 1.369(2) Å is indicative of the C=C double bond character, which aligns poorly with an extreme canonical form 2c. Thus, the crystallographic data supports the view that 2b is the most representative resonance structure of 2 compared to 2a or the extreme form 2c.

Fig. 3B provides a close-up view of the metallacyclic framework. The short V1–C31 distance of 2.110(2) Å in complex 2 hints at a possible interaction between vanadium and the β-carbon, suggesting a more delocalized structure such as 2d or even one with delocalized bonding involving a V–C_β_ interaction (2e). The [VNCC] ring is further revealed as a nearly planar with the V1–C30–C31–N3 torsion angle of 1.0(4)°. However, the metallacycle deviates from perfect square geometry, as evidenced by internal angles: V1–C30–C31 (77.4(4)°), C30–C31–N3 (116.2(0)°), C31–N3–V1 (82.8(4)°), and N3–V1–C30 (83.5(0)°). To the best of our knowledge, complex 2 represents the only structural study of an α-N-MCBD scaffold, making these observations particularly significant in understanding the bonding and geometric properties of such complexes.

### Synthesis and characterization of the β-P-VCBD scaffold

**Fig. 4:**
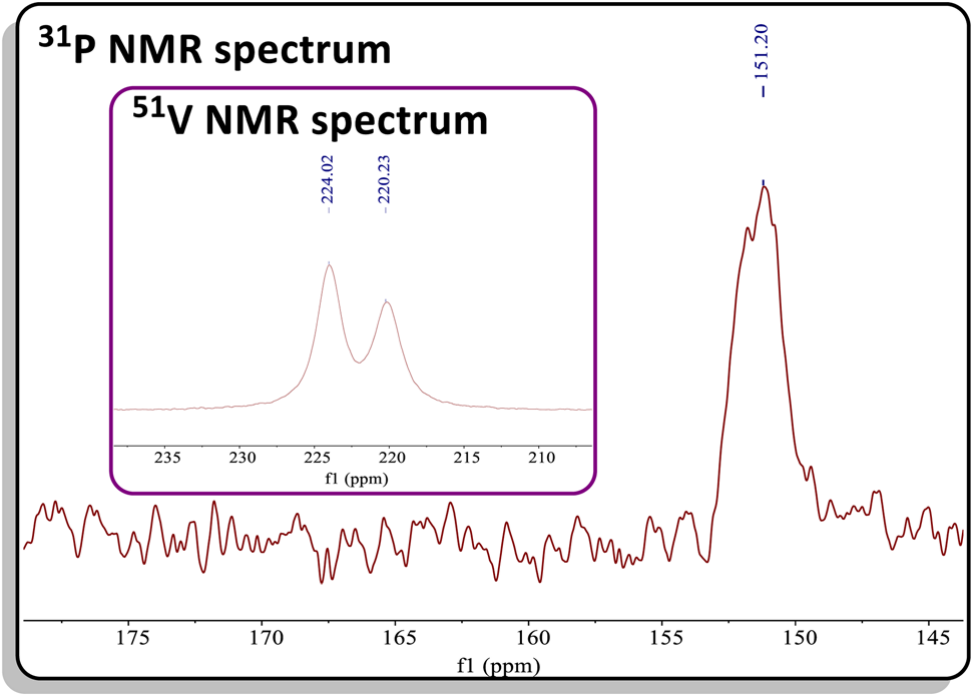
^31^P NMR spectrum of 3. Inset: ^51^V NMR spectrum of 3 showing two resonances with similar chemical shifts.

The reaction of C with P≡CAd in pentane or C_6_D_6_ at 45 °C for two hours produced a new vanadium product in 61% isolated yield, identified as the β-P-VCBD complex (dBDI)V(κ^2^-***C***,***C***-^*t*^Bu**C**P**C**Ad) (3), based on a combination of spectroscopic and structural data (Scheme 1, bottom). While one might expect Et_2_O to be a labile ligand in complex C, achieving full conversion to 3 requires not only an excess of P≡CAd but also dilute reaction conditions. This observation suggests that the cycloaddition and cycloreversion of the phosphaalkyne are in equilibrium, even in the presence of a weak donor ligand such as Et_2_O. According to spectroscopic and structural studies (*vide infra*), complex 3 exhibits overall *C*_1_ symmetry due to the absence of a σ plane or *C*_2_ elements in the dBDI^2−^ scaffold, indicating that the vanadium center in complex 3 is chiral. The ^1^H NMR spectrum of 3 (Fig. S18†) displays two pairs of inequivalent methylene hydrogens at 3.80 and 2.91 ppm, which correlates to a carbon resonance at 82.08, based on a ^1^H–^13^C HSQC spectroscopic experiment (Fig. S22 and S23†). Two distinct signals for the methine C*H* fragment of the bis-anilido backbone were also observed through the ^1^H–^13^C HSQC experiment at 5.75 and 5.69 ppm. In addition, the ^51^V NMR spectrum reveals two distinct resonances (220.0, Δ*ν*_1/2_ = 371 Hz; 223.9 ppm, Δ*ν*_1/2_ = 250 Hz), indicating the presence of two magnetically inequivalent but structurally similar species in solution^92^ (inset in Fig. 4), which is corroborated by the appearance of many more resonances in the ^13^C NMR spectrum (Fig. S19†). The ^31^P{^1^H} NMR spectrum revealed only one diagnostic feature at 151 ppm, and this resonance is quite broad (Δ*ν*_1/2_ = 437 Hz, Fig. 4). The broadening of this ^31^P{^1^H} NMR resonance, which is attributed to the β-P atom, most likely stems from coupling with the quadrupolar ^51^V nucleus (*I* = 7/2, 99.75%) as well as dynamic phenomena involving the two diastereomers of a species with *C*_1_ symmetry. Variable–Temperature (VT) NMR studies at elevated temperatures did not reveal the interconversion and coalescence of the two species, as this complex slowly decomposes above 55 °C (Fig. S20†). Additionally, it should be noted that Piers and co-workers have reported similar dynamic behavior involving the formation of *endo*/*exo* diastereomers in zwitterionic systems such as ([ArNC^*t*^Bu]_2_CH)Sc(CH_3_)[CH_3_B(C_6_F_5_)_3_] (Ar = 2,6-^*i*^Pr_2_C_6_H_3_).^72,93^ The reaction to form 3 is regioselective, but it is most likely non-stereospecific, depending on whether the P≡CAd approaches the non-planar {(dBDI)V≡C^*t*^Bu} framework from the *exo* or *endo* side to ultimately cycloadd across the V≡C^*t*^Bu ligand in C (Scheme 1). Adding to this complexity, this cycloaddition process is reversible, likely leading to the decomposition of {(dBDI)V≡C^*t*^Bu}. Based on VT NMR studies, these two conformers do not interconvert below the decomposition temperature, likely due to steric hindrance imposed by bulky substituents (*e.g.*, Ar, ^*t*^Bu, and Ad groups).

The solid-state structure of 3 is shown in Fig. 3C. Upon inspection, one can observe the short but varying V–C(^*t*^Bu) (1.817(2) Å) and V–C(Ad) (1.914(2) Å) distances, both of which fall within the range of V=C double bond lengths when compared to previously reported and structurally characterized compounds with V=C double bonds compiled in the CCDC.^40,74–91^ Additionally, the short C_α_–P distances (P1–C30, 1.802(2) Å; P1–C35, 1.727(2) Å) are comparable to those observed in structurally characterized phosphaalkenes reported in the CCDC (1.65–1.75 Å).^94^ Notably, the V1–P1 distance of 2.384(6) Å is much shorter than the sum of the van der Waals radii,^95^ which tantalizingly suggests the potential interaction between the metal center and P atom; however, such an interaction is likely to be extremely fragile, akin to those described for isolobal κ^2^-***C***,***C***-deprotiometallacyclobutadiene (dMCBD) scaffolds.^2,10,73,96–100^ A close examination of the [VCPC] ring in Fig. 3D reveals a relatively planar but more kite-like framework, with angles V1–C30–P1 (82.4(3)°), C30–P1–C35 (101.6(2)°), P1–C35–V1 (81.6(6)°), and C35–V1–C30 (94.2(9)°). The V1–C30–P1–C35 torsion angle of 0.3(0)° further emphasizes the planarity of the four-membered ring in 3 compared to 2. Given the ability of the double bonds to delocalize within the four-membered ring, complex 3 can be described as an average of two possible canonical forms, 3a and 3b, as indicated in Scheme 1, but with some contribution from the more puckered resonance structure 3c, which leads to a shortening of the vanadium distance to all other atoms in the metallacycle. Akin to complex 2, resonance structure 3c represents a more extreme scenario where the β-P could possess some formal cationic character while interacting with the electron-rich metal center. Scheme 1 also depicts a more delocalized resonance structure, 3d and 3e, similar to how the MCBD and dMCBD complex has been described in the literature for Mo,^2,16,98^ W,^96,100^ and more recently group four^36,101–103^ and five^58,104^ transition metals. It is also noteworthy that in the solid-state structure of this molecule, the Ad group points towards the dBDI^2−^ ligand, in accord with the *endo* isomer shown in Scheme 1.

### Impact of the heteroatom substitution on the structures of 2–4

In our recent studies, we synthesized the VCBD complex (dBDI)V(κ^2^-***C***,***C***-^*t*^Bu**C**C(H)**C**^*t*^Bu) (4) by treating complex C with the terminal alkyne HC≡C^*t*^Bu.^58^ The solid-state structure confirmed the diamond-like shape of the VCBD moiety, with the V–C_α_ bond lengths of 1.891(7) and 1.788(9) Å. The C_α_–C_β_ distances of 1.464(1) and 1.410(1) Å are between typical C–C single bonds and C=C double bonds. The relatively short V–C_β_ distance of 2.004(8) Å, compared to 2.110(2) Å in complex 2 and 2.384(6) Å in complex 3, suggests a potential interaction between the vanadium center and the β-carbon.^91^

With all VCBD complexes supported with the same dBDI ligand structurally confirmed, we can perform a meaningful comparison of their geometries. Table 1 provides a comparative analysis of these scaffolds, emphasizing key metrical parameters for the VCBD. One striking observation is that the V–X_α_ bond in complex 2 is significantly shorter than in complexes 3 and 4, underscoring the strong interaction between the vanadium center and the α-N atom. The stronger interaction is attributed to the higher electronegativity of the nitrogen atom, which likely facilitates a stronger bond with vanadium. Conversely, there is a gradual decrease in the V–C_α_ bond length from 2 to 4, suggesting an increasing contribution of the resonance structure featuring the V=C_α_ double bond. Moreover, the V–X_β_ bond in complex 3 is significantly longer than in complexes 2 and 4, which can be attributed to the larger covalent radius for phosphorus when compared to carbon. When comparing the C_α_–X_β_–X_α_ angle, complex 3 stands out as the closest to a square geometry, with this angle being close to 90°. On the other hand, complex 4, despite its symmetrical bonding nature, deviates the most from the square geometry with an angle of 124.3°. The notable differences in these structural parameters suggest that the inclusion of heteroatoms significantly alters the electronic properties of the VCBDs. These changes likely influence the reactivity and stability of these complexes, providing insights into the role of heteroatoms in modulating the electronic structures of the VCBDs.

**Table 1:** Selected metrical parameters for the comparison of VCBD scaffolds in complexes 2–4. Bond lengths are in Angstrom (Å), and bond angles are in degrees (°). L = dBDI^2−^ = ArNC(CH_3_)CHC(CH_2_)NAr, Ar = 2,6-^*i*^Pr_2_C_6_H_3_

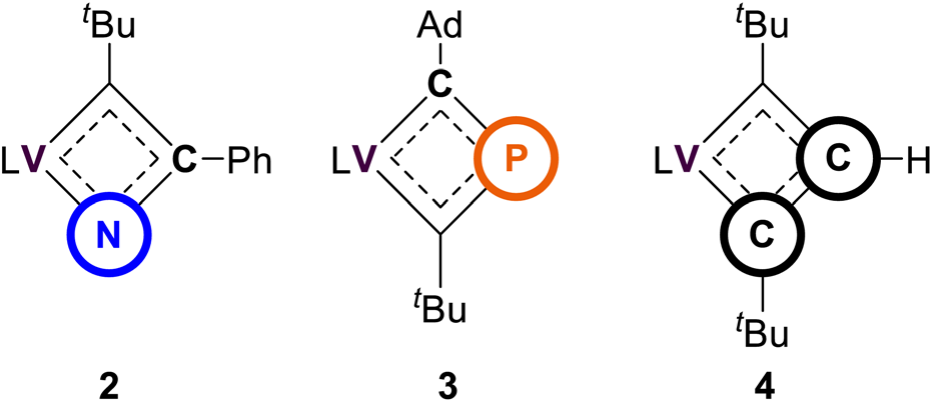			
	2 (X_α_ = N; X_β_ = C)	3 (X_α_ = C; X_β_ = P)	4 (X_α_ = X_β_ = C)
V–C_α_ (Å)	1.930(2)	1.817(2)	1.788(7)
V–X_α_ (Å)	1.671(1)	1.914(2)	1.891(7)
V–X_β_ (Å)	2.110(2)	2.384(6)	2.004(8)
C_α_–X_β_ (Å)	1.369(2)	1.802(2)	1.464(1)
X_α_–X_β_ (Å)	1.482(2)	1.727(2)	1.410(1)
V–C_α_–X_β_ (°)	77.4(4)	82.4(9)	75.3(4)
V–X_α_–X_β_ (°)	82.9(1)	81.7(8)	73.1(4)
C_α_–X_β_–X_α_ (°)	116.3(1)	101.6(1)	124.3(7)
X_α_–V–C_α_ (°)	83.5(1)	94.3(9)	87.3(3)

### Theoretical investigation on the electronic and resonance structures of 2–4

**Fig. 5:**
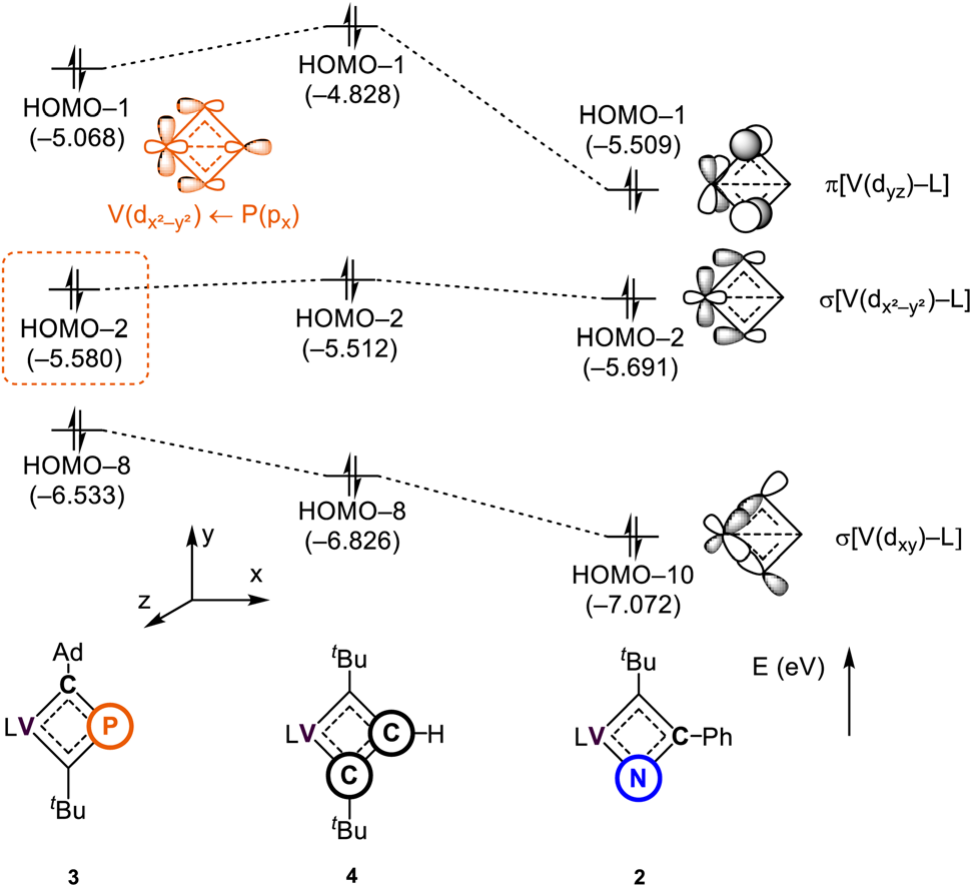
Molecular orbitals of 2–4 interacting between the metal center and the vanadacyclobutadiene scaffolds. L = dBDI^2−^ = ArNC(CH_3_)CHC(CH_2_)NAr, Ar = 2,6-^*i*^Pr_2_C_6_H_3_.

To further investigate the impact of heteroatom substitution on the electronic structures of the VCBDs, we performed DFT calculations at the PBE-D3(BJ)/TZ2P//DZP level of theory.^105–109^Fig. 5 illustrates the frontier orbitals of three complexes, highlighting primary interactions involving the VCBD ligands, identified as π, σ, and σ-bonding orbitals. The energy level of the lowest σ-bonding orbital follows the anticipated electronegativity trend of P, C, and N atoms, with significant stabilization observed in complex 2, indicating a strong V–N bond. Interestingly, an interaction between the β-P atom and the vanadium center is observed in the HOMO–2 of 3, stabilizing its energy level below that of 4. This interaction is characterized as a dative bond, where the lone pair of the β-P atom donates to the vacant d-orbital of the metal center, which is not seen in 2 and 4 due to the presence of β-(C–R) bonds (R = Ph (2) and H (4)). These findings suggest that the substitution of heteroatoms results in unique bonding interactions, where in the case of P, the V–P interaction contributes some to the stabilization of the four membered-ring. The remaining d-orbitals on vanadium form σ-bonds with the dBDI^2−^ ligand (Fig. S37†).

**Fig. 6:**
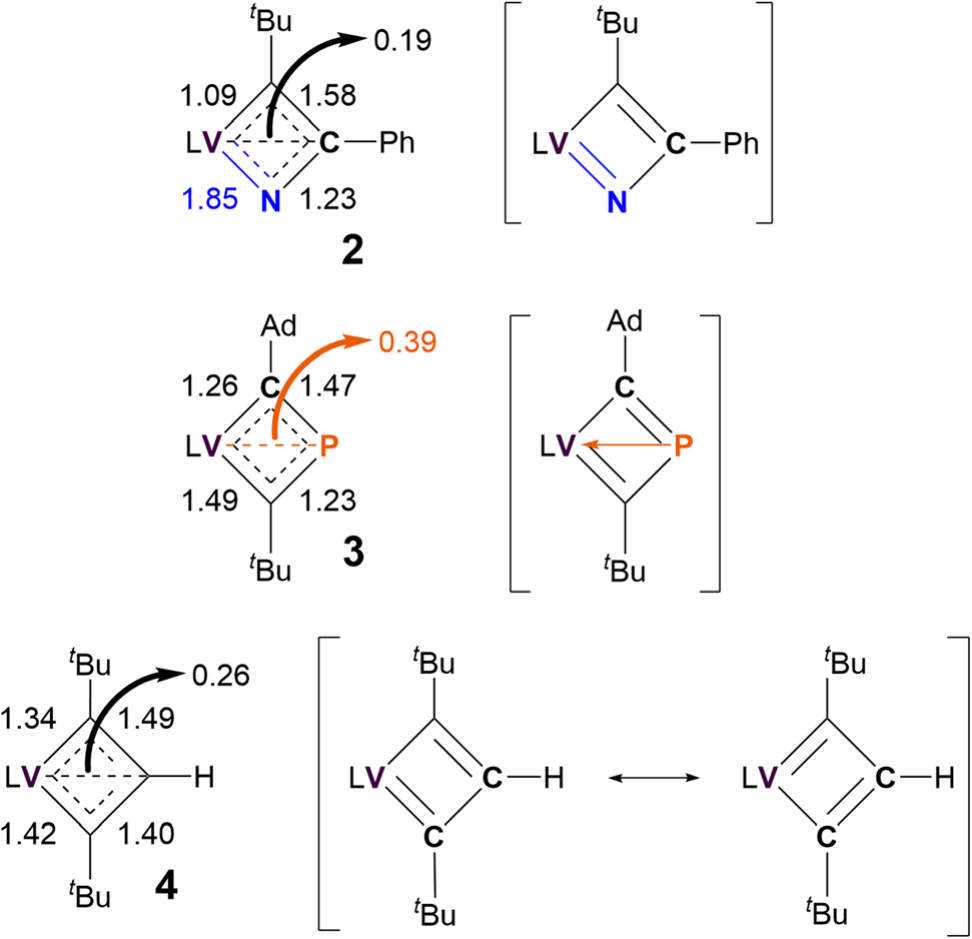
Nalewajski-Mrozek bond orders of VCBD scaffolds and most probable resonance structures of 2–4. L = dBDI^2−^ = ArNC(CH_3_)CHC(CH_2_)NAr, Ar = 2,6-^*i*^Pr_2_C_6_H_3_.

As shown in Scheme 1, the ambiguous bonding nature within the VCBD scaffolds leads to numerous possible resonance- and charge delocalized-structures for these complexes. To identify the most contributing resonance structure, we analyzed the Nalewajski-Mrozek bond order,^110^ which offers a valence bond interpretation of DFT results, similar to the Mayer bond order analysis^111^ (Fig. S39†). The corresponding bond orders and resonance structures are summarized in Fig. 6.

Unsurprisingly, the resonance between the two Lewis valence structures of the VCBD is strongest in complex 4, with the computed bond order of 1.45 being very close to the ideal resonance value of 1.5. Note, that the calculated numbers in Fig. 6 show a slight variation (1.49/1.40) due to deviations of the molecular structure from the ideal *C*_2v_ symmetry. Incorporating a strongly donating imido-like functionality in complex 2 distorts the resonance form much more toward a localized Lewis valence form where a V=N double bond character is emphasized, with a calculated bond order of 1.85. Consequently, the N–C bond has lost a notable amount of its double bond character and shows a bond order of only 1.23. Thus, the localized valence structure is a much more appropriate depiction of bonding in 2 than 4, suggesting that resonance form 2b in Scheme 1, is the most reasonable, which agrees with our scXRD analysis and optimized structure obtained *via* computational studies. In 3, the P–C bonds show an average bond order of 1.35, with the individual bond orders being 1.47 and 1.23 due to structural distortions caused by the Ad and ^*t*^Bu ligands. Thus, a localized Lewis structure is also a good representation for 3, but only for steric reasons. Interestingly, all three complexes show notable α,β-[CCC/CPC/NCC] agostic interactions^112^ that can be envisioned as direct donation from the β-atom of the metallacycle into the metal center. The formal bond orders of this interaction are 0.26, 0.39 and 0.19 for complexes 4, 3, and 2, respectively. This trend for the 3-center-4-electron interaction is easy to understand, given that the C–P bonds are more polarizable than C–C, while C–N is less polarizable than C–C. This trend suggests that complex 3 should be illustrated with charge delocalization around the four membered ring with some electronic interaction between the vanadium and the β-phosphorous atom, *i.e.* complex 3e in Scheme 1. Different electron partitioning schemes, such as atoms in molecules^113,114^ and the electron localization function^115^ analysis paint a similar picture of the electronic structure and are shown in the ESI (Fig. S40–S42).† Thus, from a purely electronic viewpoint, β-P-VCBD is best prepared to engage in C–P bond activation reactions, followed by α-N-VCBD, while the purely carbon based VCBD should in principle be the least reactive species in the series. These Lewis valence structures that best reflect the underlying electronic structure of the three complexes are shown in Fig. 6.

## Conclusions

In this study, the vanadium alkylidyne complex C has been demonstrated to undergo regiospecific [2 + 2]-cycloaddition with N≡CR (R = Ad or Ph) and P≡CAd. Based on the ^15^N NMR chemical shift observed in 50% ^15^N-enriched sample of 1, we anticipate that its geometry is like that of the Ph derivative 2, consistent with a planar α-N-VCBD where nitrogen is directly coordinated to the metal center. In the case of 3, the more electropositive phosphorus atom causes a reversal in regioselectivity, resulting in the formation of a β-P-VCBD scaffold. No evidence of cross-metathesis was observed for these complexes. The successful identification of the crystal structures of 2 and 3 allowed for a systematic comparison of their geometries with the all-carbon analogue 4, which was structurally confirmed in a previous report. This series of complexes reveals significant differences in the bonding nature between the metal center and adjacent atoms, as well as variations in the shape of the VCBD scaffolds, indicating different distributions of electron density within these metallacyclic framework. Theoretical investigations into the electronic and resonance structures of 2 and 3 suggest that the unique V–N and V–P interactions enhance the stability of the VCBD scaffolds, resulting in a single dominant resonance structure, in contrast to their analogue 4. We are currently exploring the reactivity of these metallacycles, with a particular interest in the weak V–X_β_ interactions and the Lewis basicity of the phosphorus atom in 3.

## Data availability

The data supporting this article have been included as part of the ESI.†

## Author contributions

MGJ: writing – review & editing, writing – original draft, methodology, investigation, formal analysis. JBR: writing – review & editing, writing – original draft, methodology, investigation, formal analysis. HM: formal analysis, investigation, methodology, visualization, writing – original draft, writing – review & editing; SK: formal analysis, investigation, methodology, visualization, writing – original draft, writing – review & editing. PJC: investigation, formal analysis. MRG: investigation, formal analysis. MHB: funding acquisition, resources, supervision, writing – review & editing. DJM: writing – review & editing, supervision, funding acquisition.

## Conflicts of interest

The authors declare no competing conflict of interest.

## Supplementary Material

SC-015-D4SC05884D-s001

SC-015-D4SC05884D-s002
