# Supplementary material for: Pnictogen-based vanadacyclobutadiene complexes
Source: Chem Sci. 2024 Nov 1;15(47):19752–63. doi: 10.1039/d4sc05884d (PMC11575583; doi:10.1039/d4sc05884d)
Supplement: SC-015-D4SC05884D-s001 [file SC-015-D4SC05884D-s001.pdf]

Supplementary Information (SI) for Chemical Science.
This journal is © The Royal Society of Chemistry 2024

Pnictogen–Based Vanadacyclobutadiene Complexes

Mehrafshan G. Jafari,[a] John B. Russell,[a] Hwan Myung,[b],[c] Seongyeon Kwon,[b],[c] Patrick J. Carroll,[a] Michael R. Gau,[a] Mu-Hyun Baik,[b],[c],* and Daniel J. Mindiola[a],*

[a] Prof. Dr. Daniel J. Mindiola, Department of Chemistry, University of Pennsylvania, Philadelphia, PA 19104, USA. E-mail: mindiola@sas.upenn.edu

[b] Prof. Dr., Mu-Hyun Baik, Department of Chemistry, Korea Advanced Institute of Science and Technology (KAIST), Daejeon 34141, Republic of Korea. E-mail: mbaik2805@kaist.ac.kr

[c] Center for Catalytic Hydrocarbon Functionalizations, Institute for Basic Science (IBS), Daejeon 34141, Republic of Korea.

Table of Contents

List of Figures, Tables,and Schemes 3

1. General Procedures 6

2. Syntheses 8

 2.1: (dBDI)V(κ^2-***C***,***N***-^{t}Bu**C**C(Ad)**N**) (**1**) 8

 2.2: (dBDI)V(κ^2-***C***,***N***-^{t}Bu**C**C(Ad)^{15}**N**) (**1-^{15}N**) 10

 2.3: (dBDI)V(κ^2-***C***,***N***-^{t}Bu**C**C(Ph)**N**) (**2**) 11

 2.4: (dBDI)V(κ^2-***C***,***C***-^{t}Bu**C**P**C**Ad) (**3**) 13

3. NMR Spectroscopic Data 15

 3.1: (dBDI)V(κ^2-***C***,***N***-^{t}Bu**C**C(Ad)**N**) (**1**) 15

 3.2: (dBDI)V(κ^2-***C***,***N***-^{t}Bu**C**C(Ph)**N**) (**2**) 22

 3.3: (dBDI)V(κ^2-***C***,***C***-^{t}Bu**C**P**C**Ad) (**3**) 31

4. IR Spectroscopic Data 41

5. UV-Vis Spectroscopic Data 44

 5.1: (dBDI)V(κ^2-***C***,***N***-^{t}Bu**C**C(Ad)**N**) (**1**) 44

 5.2: (dBDI)V(κ^2-***C***,***N***-^{t}Bu**C**C(Ph)**N**) (**2**) 45

 5.3: (dBDI)V(κ^2-***C***,***C***-^{t}Bu**C**P**C**Ad) (**3**) 48

6. Computational Data 51

 6.1. Functional Benchmark Study 51

 6.2. Molecular Orbital Correlation Diagram between V and dBDI 53

 6.3. Mayer Bond Order Analysis 55

 6.4. QTAIM and ELF Analysis 56

7. X-ray Crystallographic Data 59

8. Appendix 63

 8.1. Cartesian Coordinates of the Optimized Structures 63

9. References 78

List of Figures, Tables,and Schemes

Figure S1: NMR assignments of Complex **1** 15

Figure S2: ^{1}H NMR spectrum of **1** (500 MHz, C_6D_6, 300K). Elaborate assignment of all resonances is unattainable given the extensive overlaps of peaks. 16

Figure S3: ^{13}C{^{1}H} NMR spectrum of **1** (126 MHz, C_6D_6, 300K) 17

Figure S4: ^{1}H–^{1}H COSY NMR spectrum of **1** (500 MHz, C_6D_6, 300K) 18

Figure S5: ^{15}N{^{1}H} NMR spectrum of **1-^{15}N** (61 MHz, C_6D_6, 300K). The resonance at 242pm corresponds to the internal standard (^{15}N≡CAd). 19

Figure S6: ^{51}V NMR spectrum of **1** (132 MHz, C_6D_6, 300K) 20

Figure S7: Stacked ^{1}H NMR spectrum of **1** (500 MHz, C_6D_6) at varible temperatures. 298K (red, bottom), 288K (olive green, 2nd from bottom), 278K (forrest green, middle), 268K (blue, 2nd from top), 258K (purple, top). 21

Figure S8: NMR assignments of Complex **2** 22

Figure S9: ^{1}H NMR spectrum of **2** (600 MHz, C_6D_6, 300K). The resonance assigned by * arises from residual toluene. 23

Figure S10: ^{13}C{^{1}H} NMR spectrum of **2** (151 MHz, C_6D_6, 300K) 24

Figure S11: ^{13}C{^{1}H} DEPT–135 NMR spectrum of **2** (151 MHz, C_6D_6, 300K) 25

Figure S12: ^{1}H–^{1}H COSY NMR spectrum of **2** (600 MHz, C_6D_6, 300K) 26

Figure S13: ^{1}H–^{13}C HSQC NMR spectrum of **2** ((600 MHz,151 MHz), C_6D_6, 300K) 27

Figure S14: ^{1}H–^{13}C HMBC NMR spectrum of **2** ((600 MHz, 151 MHz), C_6D_6, 300K) 28

Figure S15: ^{51}V NMR spectrum of **2** (132 MHz, C_6D_6, 300K) 29

Figure S16: Stacked ^{1}H NMR spectrum of **2** (500 MHz, C_6D_6) at varible temperatures. 30

Figure S17: NMR assignments of Complex **3** 31

Figure S18: ^{1}H NMR spectrum of **3** (400 MHz, C_6D_6, 300K). Elaborate assignment of all resonances is unattainable given the extensive overlaps of peaks. Some P≡CAd impurity also remain in the sample. 32

Figure S19: ^{13}C{^{1}H} NMR spectrum of **3** (126 MHz, C_6D_6, 300K). The insets show various resonances that appear as two sets of resonances close in chemical shift. 33

Figure S20: Stacked ^{1}H NMR spectrum of **3** (500 MHz, C_6D_6) at varible temperatures. 298K (red, bottom), 308K (green, 2nd from bottom), 318K (blue, 2nd from top), 328K (purple, top). .. 34

Figure S21: $^{1}H–^{1}H$ COSY NMR spectrum of **3** (500 MHz, C_6D_6, 300 K)................................ 35
Figure S22: $^{1}H–^{13}C$ HSQC NMR spectrum of **3** ((600 MHz,151 MHz), C_6D_6, 300K).............. 36
Figure S23: A zoomed in section of the $^{1}H–^{13}C$ HSQC NMR spectrum of **3** ((600 MHz,151 MHz), C_6D_6, 300K). This highlights the pair of inequivalent methylene (=C*H*$_2$) hydrogens at 3.80 and 2.91 ppm in the ^{1}H NMR are correlated to the same carbon resonance at 82.08 ppm. Additionally, the methine (C*H*) fragment of the bis anilido ligand appears as two distinct signals at (5.75 ppm, 104.78 ppm) and (5.69 ppm, 104.14 ppm) in this spectrum. This suggests that complex **3** exhibits two magnetically inequivalent but structurally similar species in solution... 37
Figure S24: $^{1}H–^{13}C$ HMBC NMR spectrum of **3** ((600 MHz,151 MHz), C_6D_6, 300K)............. 38
Figure S25: $^{31}P\{^{1}H\}$ NMR spectrum of **3** (202 MHz, C_6D_6, 300K). The resonance at -68.21 pm corresponds to the internal standard (P≡CAd).. 39
Figure S26: ^{51}V NMR spectrum of **3** (132 MHz, C_6D_6, 300K).. 40
Figure S27: IR spectra of **1** *vs.* **C**.. 41
Figure S28: IR spectra of **2** *vs.* **C**.. 42
Figure S29: IR spectra of **3** *vs.* **C**.. 43
Figure S30: UV-Vis spectrum of **1** in pentane at various concentrations (red trace = 7.1×10^{-4} M and blue trace = $3.6\ 1 \times 10^{-4}$ M).. 44
Figure S31: UV-Vis spectrum of **2** in pentane at various concentrations (red trace = 8.0×10^{-4} M and black trace = 3.4×10^{-4} M).. 45
Figure S32: UV-Vis spectrum of a more concentrated sample of **2** in pentane (8.0×10^{-4} M)... 46
Figure S33: UV-Vis spectrum of a more dilute solution of **2** in pentane (3.4×10^{-4} M). 47
Figure S34: UV-Vis spectrum of **3** in pentane with various concentrations (red trace = 7.2×10^{-4} M, green trace = 2.4×10^{-4} M, blue trace = 1.3×10^{-4} M, and black trace = 6.0×10^{-5} M). 48
Figure S35: UV-Vis spectrum of concentrated sample of **3** in pentane (7.2×10^{-4} M). 49
Figure S36: UV-Vis spectrum of dilute sample of **3** in pentane (6.0×10^{-5} M). 50
Figure S37: Molecular orbitals of **2-4** interacting between the metal center and the dBDI ligands 53
Figure S38: Molecular orbital correlation diagram of **4** describing the interactions between Vd_{xz}, BDInb, and $L\pi^*$. 54
Figure S39: Mayer bond order between atoms in the metallacyclobutadienes. 55

Figure S40: (Left) QTAIM plot and (Right) ELF plot of the MCBD scaffold in complex **2**. Blue lines indicate bond path points, and red dots indicate bond critical points................................. 56
Figure S41: (Left) QTAIM plot and (Right) ELF plot of the MCBD scaffold in complex **3**. Blue lines indicate bond path points, and red dots indicate bond critical points................................. 57
Figure S42: (Left) QTAIM plot and (Right) ELF plot of the MCBD scaffold in complex **4**. Blue lines indicate bond path points, and red dots indicate bond critical points................................. 58
Figure S43: ORTEP drawing of **2** showing thermal ellipsoids with 50% probability level. Hydrogen atoms have been omitted for clarity.. 60
Figure S44: ORTEP drawing of **3** showing thermal ellipsoids with 50% probability level. Hydrogen atoms (except salient hydrogens of the ligand backbone) have been omitted for clarity 61
Scheme S1: Synthesis of Complex **1**... 8
Scheme S2: Synthesis of Complex **1-^{15}N**.. 10
Scheme S3: Synthesis of Complex **2**... 11
Scheme S4: Synthesis of Complex **3**... 13
Table S1: MAE and RMSE of key atomic distances in **2-4** computed with various functionals. 52
Table S2: Summary of structure determination of compounds **2** and **3**...................................... 62

1. General Procedures

All operations were performed in a M. Braun glove box or using standard Schlenk techniques under a nitrogen atmosphere unless otherwise stated. Anhydrous solvents were purchased from Fisher Scientific or Aldrich. Hexamethyldisiloxane was dried by reflux in the presence of calcium hydride overnight, followed by distillation into a Straus flask. All other solvents were purified and dried by passage through two columns of activated alumina and Q-5 drying agent in a Grubbs-type solvent system. Stabilizer-free ethereal solvents, diethyl ether (Et_2O) and tetrahydrofuran (THF), were purchased from Alfa Aesar and dried by passage through two columns of activated alumina. All bulk solvents were kept over sodium and 4 Å molecular sieves. Benzene–*d*$_6$ (C_6D_6, Cambridge Isotope Laboratories) was dried and degassed over a potassium mirror and vacuum transferred to another flask prior to use. Celite and 4 Å molecular sieves were activated under vacuum overnight at 200 °C. Compound **C** was prepared according to the reported procedure.[1] 1-adamantyl carbonitrile, N≡CAd, was sublimed under vacuum at 80 °C prior to use and benzonitrile, N≡CPh, was distilled under vacuum at a slightly elevated temperature. ^{15}N-labeled 1-adamantyl carbonitrile, ^{15}N≡CAd, was prepared as previously reported.[2] 1-adamantyl phosphaethyne, P≡CAd, was brought into the glovebox as received and re-crystallized from a concentrated pentane solution and the crystals were dried under vacuum at room temperature prior to use.

NMR spectroscopic studies were recorded on Bruker NEO 600 MHz, AVIII Bio500, UNI400 or AVIII 500 MHz spectrometers. ^{1}H and ^{13}C NMR chemical shifts are reported referenced to the internal proton or carbon resonances of C_6D_6 (δ = 7.15 or 128.06). ^{15}N NMR and ^{31}P NMR chemical shifts were referenced to ^{15}N≡CAd (δ = 242 vs. NH_3(l) at 0.0) and ^{31}P≡CAd (δ = -68.21 vs. 85% H_3PO_4 in D_2O at 0.0), respectively, which were used as internal standards. For ^{51}V NMR, the reference is $VOCl_3$ in C_6D_6, which is defined as δ = 0.0.

FT-IR spectroscopic studies were measured on JASCO FT/IR-4600 instrument and all IR samples were prepared with KBr plates using the JASCO tablet master device.

UV-Vis spectroscopic data were obtained on a Cary 5000 UV-Vis-NIR spectrophotometer (Agilent Technologies) using sealed 1 cm quartz cell vessels with a J-Young valve.

Computational details had all density functional theory (DFT)[3] calculations performed using the AMS 2024 package program.[4] No frozen cores were used to ensure an all-electron basis set, and the integration accuracy was set to 'good' numerical quality. Based on the functional benchmark study to be discussed in Section 6, we selected the PBE functional[5] with the Grimme's D3 dispersion correction[6] and the Becke-Johnson damping function[7] for geometry optimizations and electronic structure analysis. Geometry optimizations were carried out with the DZP basis set,[8] and single-point calculations were performed at the same geometry with the high-quality triple-zeta basis set TZ2P.

The quantum theory of atoms in molecules (QTAIM)[9, 10] and the electron localization function (ELF)[11] for each complex were analyzed at the same level of theory used for the electronic structure analysis. For the QTAIM analysis, the initial cartesian grid spacing was set to 0.5 Bohr to ensure sufficient accuracy in the critical point search, and the electron density from -0.3 to 0.3 are plotted. ELF was calculated using the densf program implemented in the AMS 2024 package, with the grid point distances set to 0.1 Bohr to achieve high-resolution density mapping, and ELF values between 1.00×10^{-10} to 0.98 are plotted. The charge density and kinetic energy density were derived from the self-consistent field obtained from single-point calculations.

2. Syntheses

2.1: (dBDI)V(κ^2-***C*****,*****N***-^{t}Bu**C**C(Ad)**N**) (**1**)

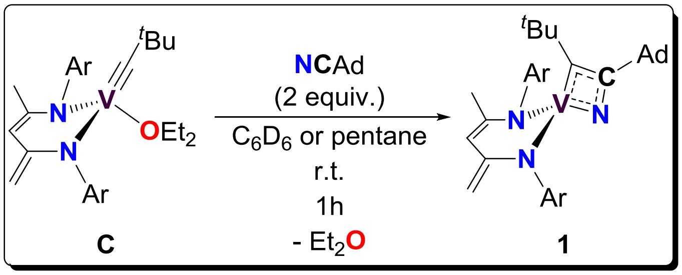

Scheme S1: Synthesis of Complex **1**

Compound **C** (100 mg, 163.5 µmol) and N≡CAd (52.7 mg, 326.9 µmol) were dissolved in 20 ml pentane (or ≈ 1 ml C_6D_6) and the solution was stirred at 45 °C for one hour, resulting in a color change from red to brown. To assist full conversion to **1**, the diethyl ether side-product was removed twice over the course of the reaction *in vacuo*. After one hour, all solvents were removed *in vacuo* and the brown product was dissolved in minimum amount of hexamethyldisiloxane (ca. 5 mL) and was filtered through Celite. Thereafter, the solution was further concentrated under vacuum and left inside the freezer at -35 °C overnight, which results in precipitation of **1**. Consequently, the product was dried *in vacuo* after decantation of the mother liquor. Yield: 55.3 mg (two crops, 48.5 %). Hexamethyldisiloxane is used only due to the high solubility of the product in pentane, merely, granting a better yield. Crystals of **1** suitable for X-ray crystallography were grown by concentrating the pentane solution of **1** at -35 °C using toluene as the sorbent.

Note: This reaction is sluggish, and the formed product is unstable and decomposes over time. **^{1}H NMR** (500 MHz, C_6D_6, 300K): δ 7.19 (m, 2H), 7.10 (m, ~2H), 7.04 (m, 1H), 6.93 (m, 1H), 5.70 (d, 0.5 H), 5.64 (d, 0.5 H), 4.00 (m, 2H), 3.76 (d by virtue, 1H), 3.29 (d by virtue, 1H), 2.77 (m, 1H), 2.47 (d, 1H), 2.40 (d, 1H), 2.33 (m, 1H), 2.11, 2.06, 2.02, 1.99, 1.97, 1.96, 1.86, 1.80, 1.75, 1.71, 1.68, 1.65, 1.60, 1.59, 1.57, 1.56, 1.51, 1.47, 1.30, 1.29, 1.24, 1.21, 1.12, 1.01, 0.94, 0.87. **^{13}C{^{1}H} NMR** (126 MHz, C_6D_6, 300K): δ 163.07, 153.55, 153.41, 153.27, 147.03, 146.83, 146.59, 146.50, 143.96, 143.22, 143.01, 142.83, 142.06, 127.15, 126.96, 124.96, 124.29, 123.66, 111.82, 87.89, 85.90, 65.89, 45.28, 44.89, 44.57, 44.11, 43.99, 43.47, 43.35, 42.49, 42.19, 37.11, 36.78, 34.46, 33.72, 33.44, 31.86, 30.98, 30.74, 29.67, 28.99, 28.77, 28.06, 27.76, 26.72, 25.77,

25.47, 25.25, 24.99, 24.91, 24.70, 22.74, 14.30. **^{15}N NMR**(61 MHz, C_6D_6, 300K): δ 761.92 (FWHM= 78.6 Hz) for **1-^{15}N** (see **Section 2.2**). **^{51}V NMR**, 132 MHz, C_6D_6, 300K): δ (ppm) 381.32 (FWHM= 339.2 Hz). **UV/Vis**, pentane, λ [nm, ε (max/sh, M^{-1} cm^{-1})]: 685 (sh, 634), 650 (sh, 704), 488 (max, 1528). **IR** (solid, KBr pellet): 593, 608, 613, 629, 641, 677, 760, 797, 854, 935, 991, 1014, 1056, 1100, 1147, 1176, 1201, 1254, 1318, 1344, 1362, 1383, 1436, 1457, 1528, 1684, 1696, 1700, 2850, 2866, 2903, 2962, 3035, 3056, 3090.

2.2: (dBDI)V(κ^2-***C***,***N***-^{t}Bu**C**C(Ad)^{15}**N**) (**1-^{15}N**)

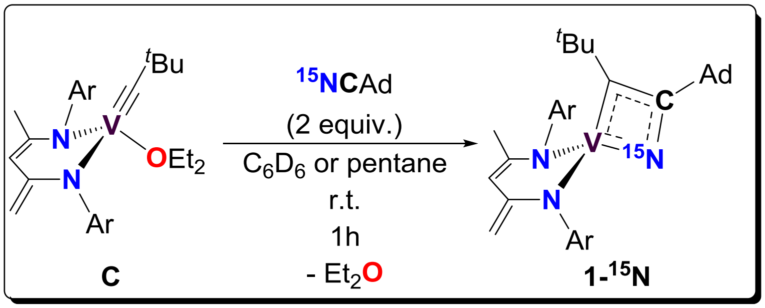

Scheme S2: Synthesis of Complex **1-^{15}N**

Compound **C** (1 equiv.) and ^{15}N≡CAd (1 equiv.) were dissolved in 20 ml pentane (or ≈ 1 ml C_6D_6) and the solution was stirred at 45 °C for one hour, resulting in a color change from red to brown. To assist full conversion to **1-^{15}N**, the diethyl ether side-product was removed twice over the course of the reaction *in vacuo*. After one hour, all solvents were removed *in vacuo* and the brown product was dissolved in minimum amount of hexamethyldisiloxane (ca. 5 mL) and was filtered through Celite. Thereafter, the solution was further concentrated under vacuum and left inside the freezer at -35 °C which results in precipitation of **1-^{15}N**. Consequently, the product was dried *in vacuo* after decantation of the mother liquor. Spectral features are listed in Section 2.1 and do not differ significantly from **1-^{15}N**.

2.3: (dBDI)V(κ^2-***C***,***N***-^{t}Bu**C**C(Ph)**N**) (**2**)

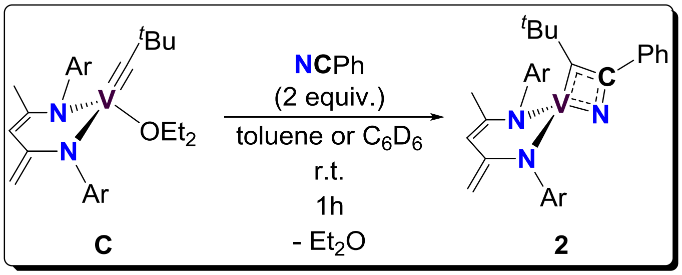

Scheme S3: Synthesis of Complex **2**

Compound **C** (50 mg, 81.7 µmol) was dissolved in ca. 3 ml toluene (or ≈ 1 ml C_6D_6) and to the solution was added benzonitrile (8.5 µL, 82.4 µmol), resulting in a rapid color change from red to purple. After 30 minutes, all volatiles were removed *in vacuo* and the purple product was dissolved in minimum amount of pentane (ca. 3-4 mL) and was filtered through Celite. Thereafter, the solution was further concentrated under vacuum and was crystallized by concentrating the solution further at -35 °C inside the freezer, using toluene as the sorbent. Consequently, the product was dried *in vacuo* after decantation of the mother liquor. Yield: 46.5 mg (two crops, 89 %). Crystals of **2** suitable for X-ray crystallography were grown by concentrating the pentane solution of **2** at -35 °C using toluene as the sorbent.

^{1}H NMR (600 MHz, C_6D_6, 300K): δ 7.51 (d, 2H, $^3J_{H–H}$=7.4 Hz), 7.23 (d, 1H, $^3J_{H–H}$=7.7 Hz), 7.07 (t, 2H, $^3J_{H–H}$=7.7 Hz), 7.03 (d, 2H, $^3J_{H–H}$=7.7 Hz), 7.00 (d, 2H, $^3J_{H–H}$=7.4 Hz), 6.98 (t, 1H, $^3J_{H–H}$=7.4 Hz), 6.89 (d, 1H, $^3J_{H–H}$=7.7 Hz), 5.69 (s, 1H), 4.01 (septet, 2H, $^3J_{H–H}$=6.9 Hz), 3.96 (septet, 2H, $^3J_{H–H}$=6.8 Hz), 3.75 (d by virtue, 1H), 3.25 (d by virtue, 1H), 2.72 (septet, 1H, $^3J_{H–H}$=6.9 Hz), 2.03 (septet, 1H, $^3J_{H–H}$=6.8 Hz), 1.67 (d, 3H, $^3J_{H–H}$=6.9 Hz), 1.58 (s, 9H), 1.55(s, 3H), 1.49 (t(dd by virtue), 6H, $^3J_{H–H}$=6.9 Hz), 1.28 (d, 3H, $^3J_{H–H}$=6.8 Hz), 1.21 (d, 3H, $^3J_{H–H}$=6.9 Hz), 0.97 (t(dd by virtue), 6H, $^3J_{H–H}$= 6.8 Hz), 0.85 (d, 3H, $^3J_{H–H}$=6.8 Hz). **^{13}C{^{1}H} NMR** (151 MHz, C_6D_6, 300K): δ 156.96, 152.85, 152.41, 146.33, 146.16, 145.81, 144.33, 142.71, 142.68, 142.53, 142.51, 141.86, 141.77, 137.89, 134.90, 131.97, 130.19, 129.85, 129.33, 129.05, 128.92, 128.57, 128.44, 127.61, 127.20, 126.86, 126.44, 125.70, 124.76, 124.48, 124.24, 124.10, 123.94, 110.47, 85.07, 41.95, 34.44, 32.14, 31.17, 30.05, 29.10, 28.83, 27.66, 27.50, 27.04, 25.86, 25.71, 25.36, 25.26, 24.99, 24.97, 24.89, 24.53, 22.76, 21.76, 21.43, 14.28. **^{51}V NMR**, 132 MHz, C_6D_6, 300K): δ 344.76 (FWHM= 276.0 Hz). **UV/Vis**, pentane, λ [nm, ε (max/sh, M^{-1} cm^{-1})]: 1053 (max, 114),

743 (sh, 499), 663 (sh, 646), 492 (max, 1903). **IR** (solid, KBr pellet): 677, 701, 735, 754, 767, 799, 823, 839, 869, 916, 936, 954, 1005, 1013, 1109, 1145, 1178, 1204, 1250, 1292, 1321, 1361, 1383, 1434, 1462, 1535, 2866, 2926, 2961, 3034, 3058.

2.4: (dBDI)V(κ^2-***C***,***C***-^{t}Bu**C**P**C**Ad) (**3**)

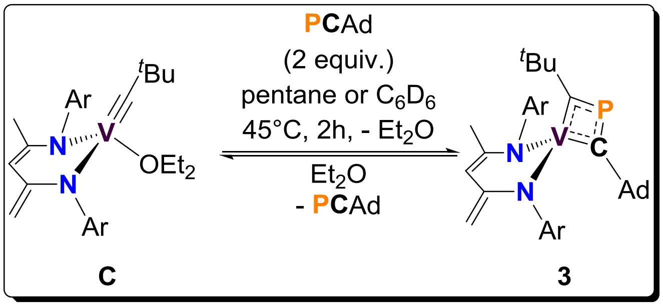

Scheme S4: Synthesis of Complex **3**

Compound **C** (200 mg, 326.9 µmol) and P≡CAd (116.5 mg, 653.7 µmol) were dissolved in 20 ml pentane (or ≈ 1 ml C_6D_6) and the solution was transferred to a sealed J-young flask. The sealed reaction vessel was brought outside the glovebox and was heated at 45 °C for two hours, resulting in a gradual color change from red to brown. The reaction vessel was transferred back into the glovebox where the solvent was removed *in vacuo*. The brown product was dissolved in minimum amount of hexamethyldisiloxane and was filtered through Celite. Solution was concentrated *in vacuo* (ca. 3 mL) and was left inside the freezer at -35 °C overnight. Thereafter, the mother liquor was decanted, and the brown precipitated product was dried under vacuum. Yield: 143.5 mg (two crops, 61.4 %). Hexamethyldisiloxane is used only due to the high solubility of the product in pentane, merely granting a better yield. Crystals of **3** suitable for X-ray crystallography were grown by concentrating the pentane solution of **3** at -35 °C using toluene as the sorbent.

Note: For full conversion of **C** to **3**, the addition of excess P≡CAd was deemed necessary. Moreover, the reaction either requires dilute conditions (as mentioned above) or in the case of a reaction in more concentrated conditions, removal of the side product diethyl ether is necessary, over the course of the reaction. It is also noteworthy to mention that the formed product is unstable in a diethyl ether solution and decomposes over time. **^{1}H NMR** (400 MHz, C_6D_6, 300K): δ 7.25 (m, 2H), 7.07 (m, ~3H, residual toluene), 7.00 (m, 2H), 6.80 (d, 1H, ^3J= 6.7 Hz), 6.69 (d, 0.45H, ^3J= 5.4 Hz), 5.73 (s, 0.55H, FWHM=7.0 Hz), 5.67 (s, 1H, FWHM=7.2 Hz), 4.53 (m, 1H, FWHM= 21.9 Hz), 4.38 (m, 1H, FWHM= 24.5 Hz), 3.79 (s, 1H, FWHM= 8.8 Hz), 2.90 (s, 1H, FWHM= 8.3 Hz), 2.55 (s,3H, FWHM=9.5 Hz), 2.19 (s, ~3H), 2.10 (s, ~2H), 2.05, 1.89,

1.84, 1.70, 1.61, 1.56, 1.47, 1.41, 1.39, 1.34, 1.27, 1.06, 0.86, 0.80, 0.47, 0.41. (extensive overlap of peaks in the aliphatic region) **^{13}C{^{1}H} NMR** (126 MHz, C_6D_6, 300K): δ 184.69 (d, $^{1}J_{C-P}$ = 34.1 Hz), 152.03, 147.69, 146.75, 143.55, 142.95, 142.57, 139.42, 139.07, 123.97, 123.64, 105.18, 104.56, 82.41, 48.71, 46.13, 43.39, 38.89, 38.75, 37.16, 36.51, 36.21, 35.72, 33.31, 30.24, 29.56, 29.07, 28.59, 28.18, 26.67, 26.05, 25.46, 25.23, 24.96, 24.64, 23.88, 23.03, 22.73, 22.56, 22.23, 22.11, 21.94. **^{31}P{^{1}H} NMR**, 202 MHz, C_6D_6, 300K): δ 151.2 (m, FWHM= 437.1 Hz). **^{51}V NMR**, 132 MHz, C_6D_6, 300K): δ 219.96 , 223.86. **UV/Vis**, pentane, λ [nm, ε (max/sh, M^{-1} cm^{-1})]: 745 (sh, 202), 326 (max, 9108). **IR** (solid, KBr pellet): 516, 519, 529, 544, 579, 589, 668, 728, 741, 752, 779, 792, 827, 861, 908, 926, 935, 954, 996, 1015, 1032, 1099, 1108, 1152, 1176, 1195, 1209, 1254, 1295, 1308, 1334, 1360, 1369, 1381, 1434, 1448, 1462, 1536, 1585, 2847, 2898, 2962, 3019, 3056, 3106.

3. NMR Spectroscopic Data

3.1: (dBDI)V(κ^2-***C***,***N***-^{t}Bu**C**C(Ad)**N**) (**1**)

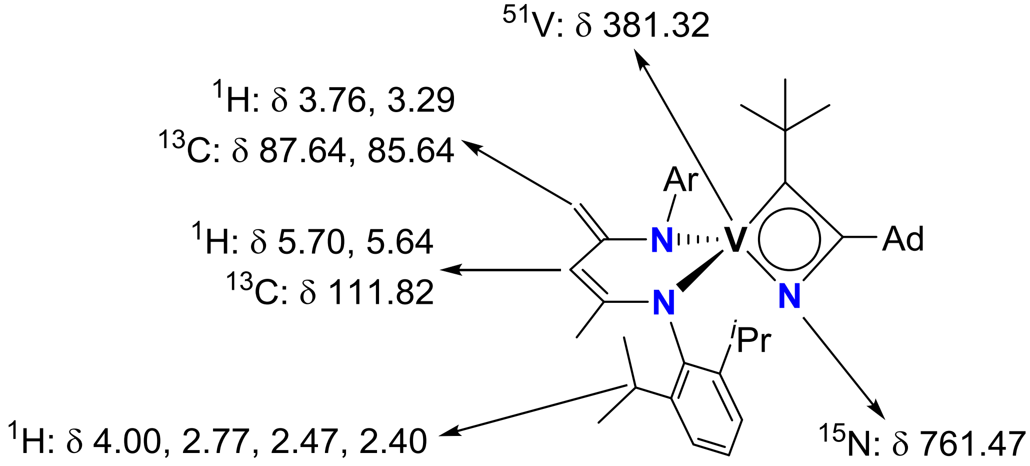

Figure S1: NMR assignments of **1**

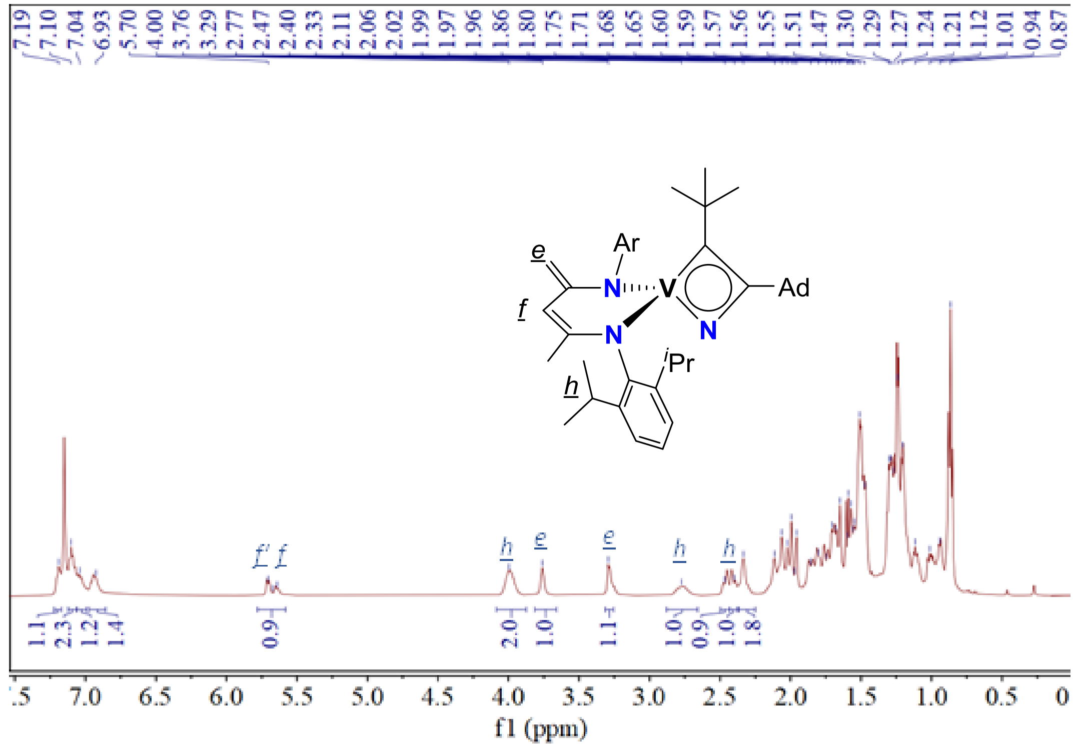

Figure S2: ^{1}H NMR spectrum of **1** (500 MHz, C_6D_6, 300K). Elaborate assignment of all resonances is unattainable given the extensive overlaps of peaks.

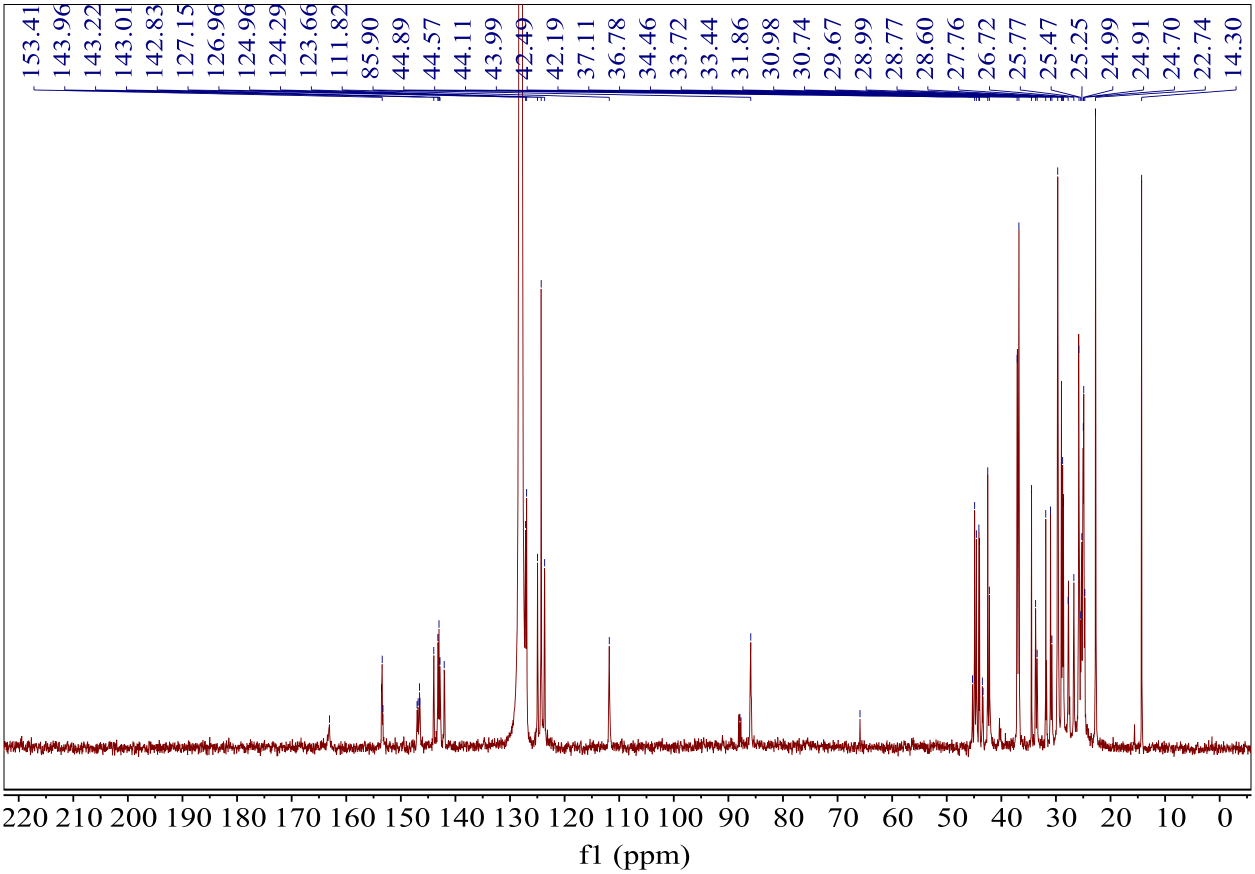

Figure S3: $^{13}C\{^{1}H\}$ NMR spectrum of **1** (126 MHz, C_6D_6, 300K)

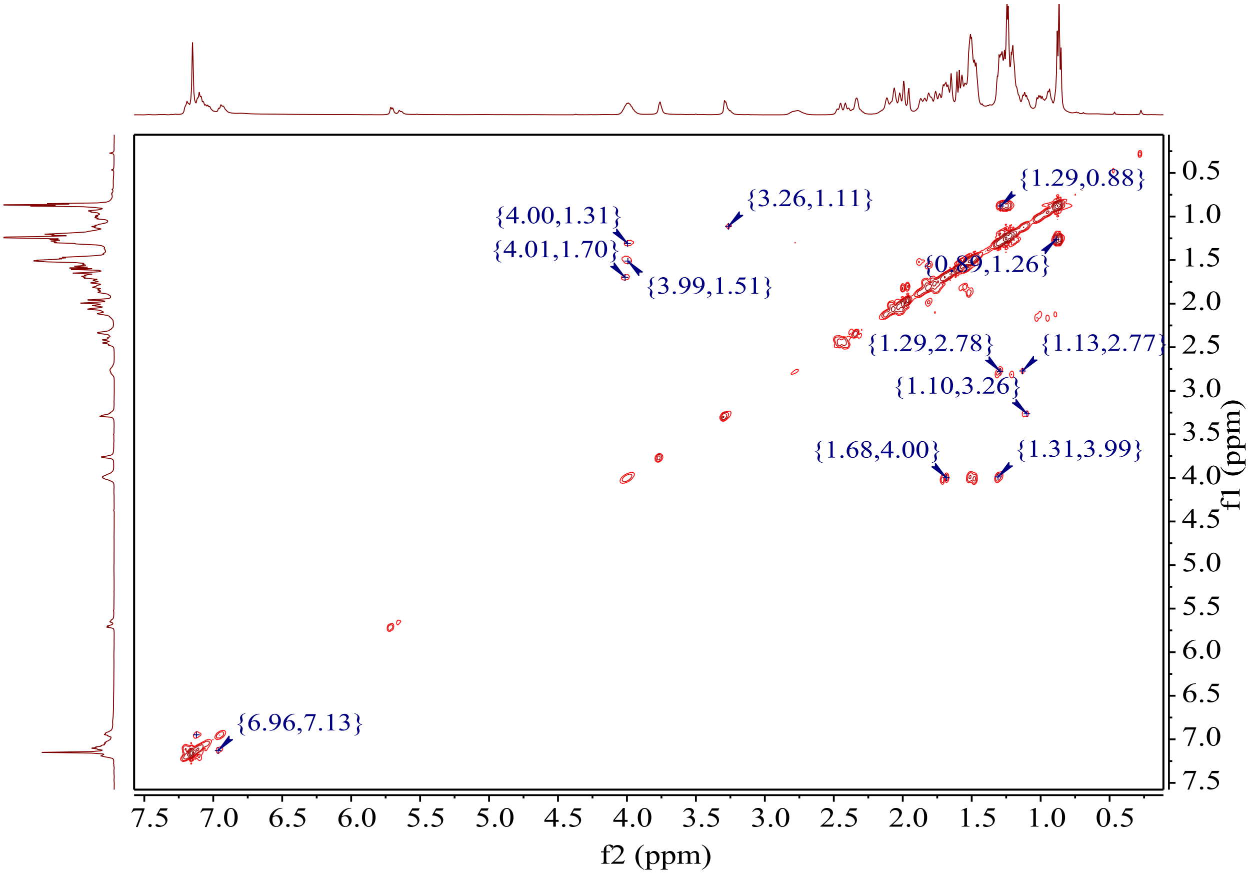

Figure S4: ^{1}H–^{1}H COSY NMR spectrum of **1** (500 MHz, C_6D_6, 300K)

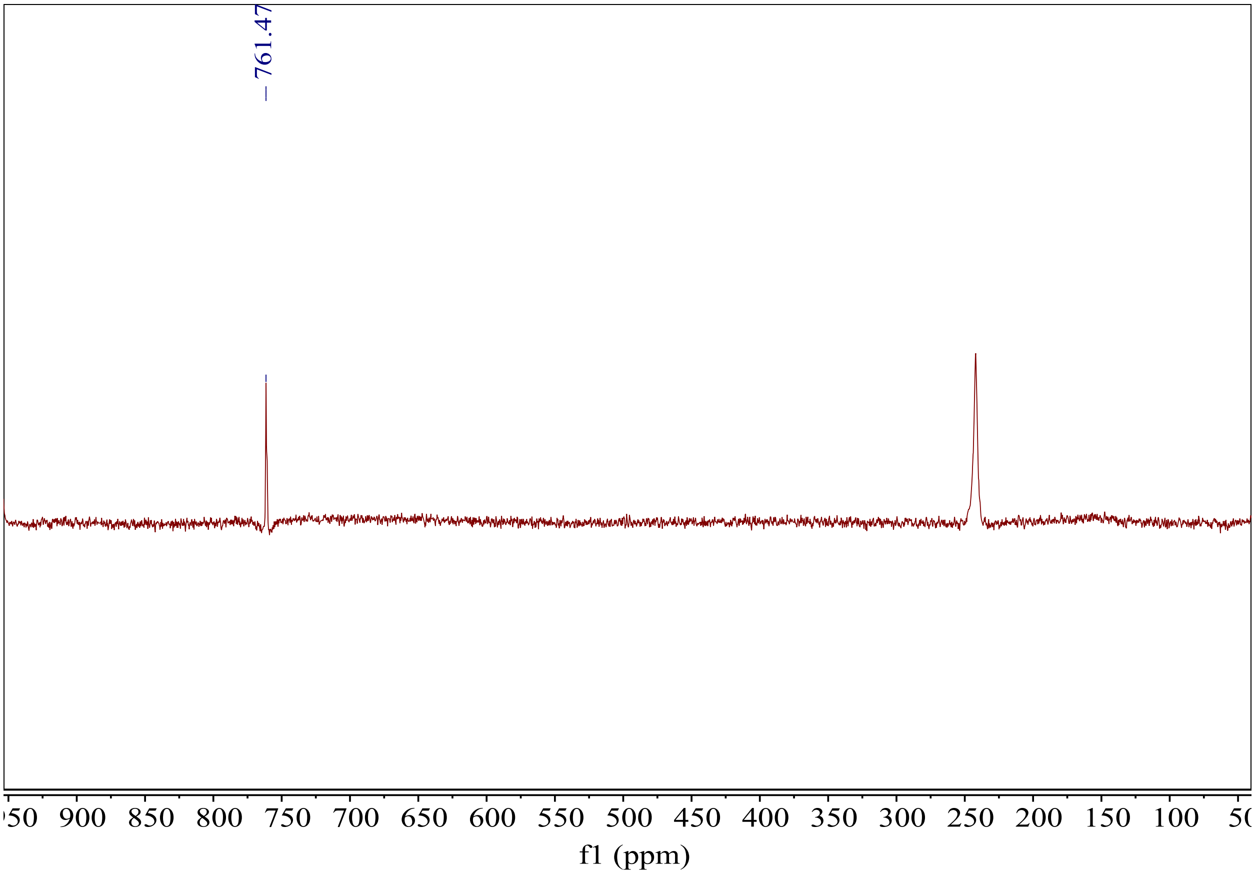

Figure S5: $^{15}N\{^{1}H\}$ NMR spectrum of **1-^{15}N** (61 MHz, C_6D_6, 300K). The resonance at 242pm corresponds to the internal standard ($^{15}N{\equiv}CAd$).

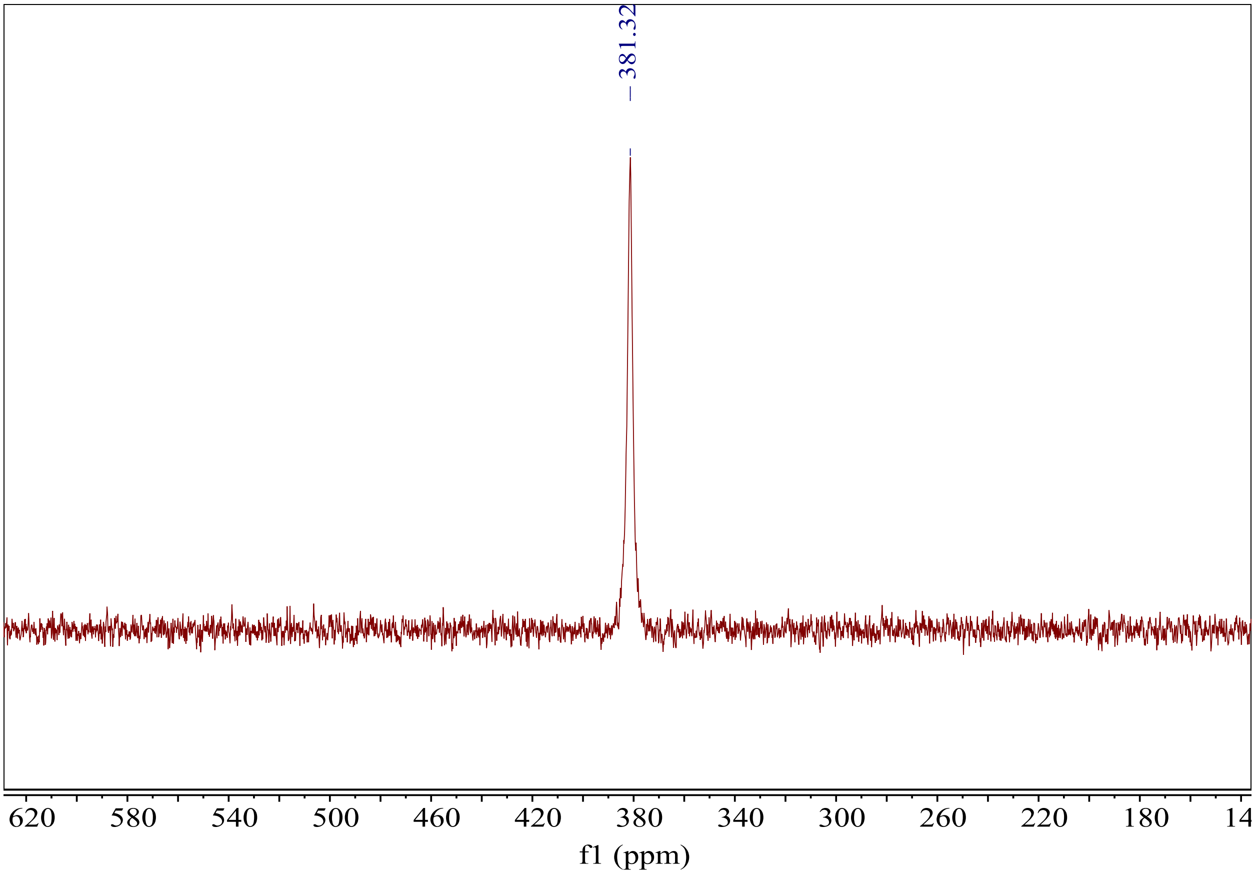

Figure S6: ^{51}V NMR spectrum of **1** (132 MHz, C_6D_6, 300K)

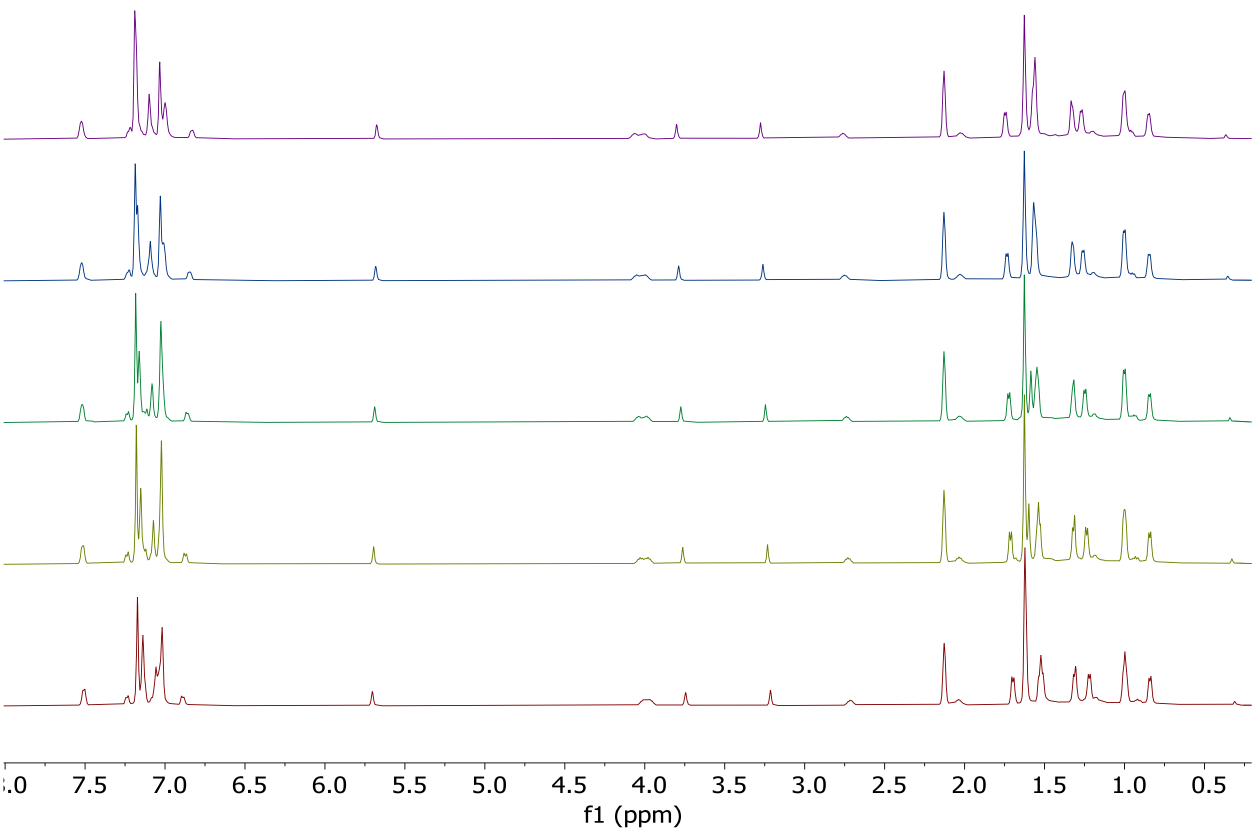

Figure S7: Stacked ^{1}H NMR spectrum of **1** (500 MHz, C_6D_6) at varible temperatures. 298K (red, bottom), 288K (olive green, 2nd from bottom), 278K (forrest green, middle), 268K (blue, 2nd from top), 258K (purple, top).

3.2: (dBDI)V(κ^2-***C***,***N***-^{t}Bu**C**C(Ph)**N**) (**2**)

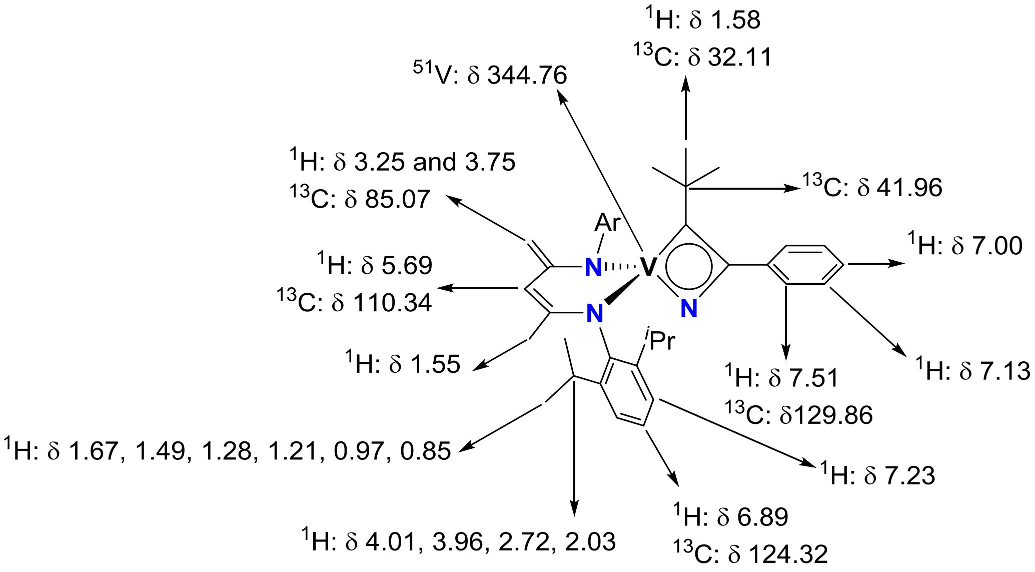

Figure S8: NMR assignments of **2**

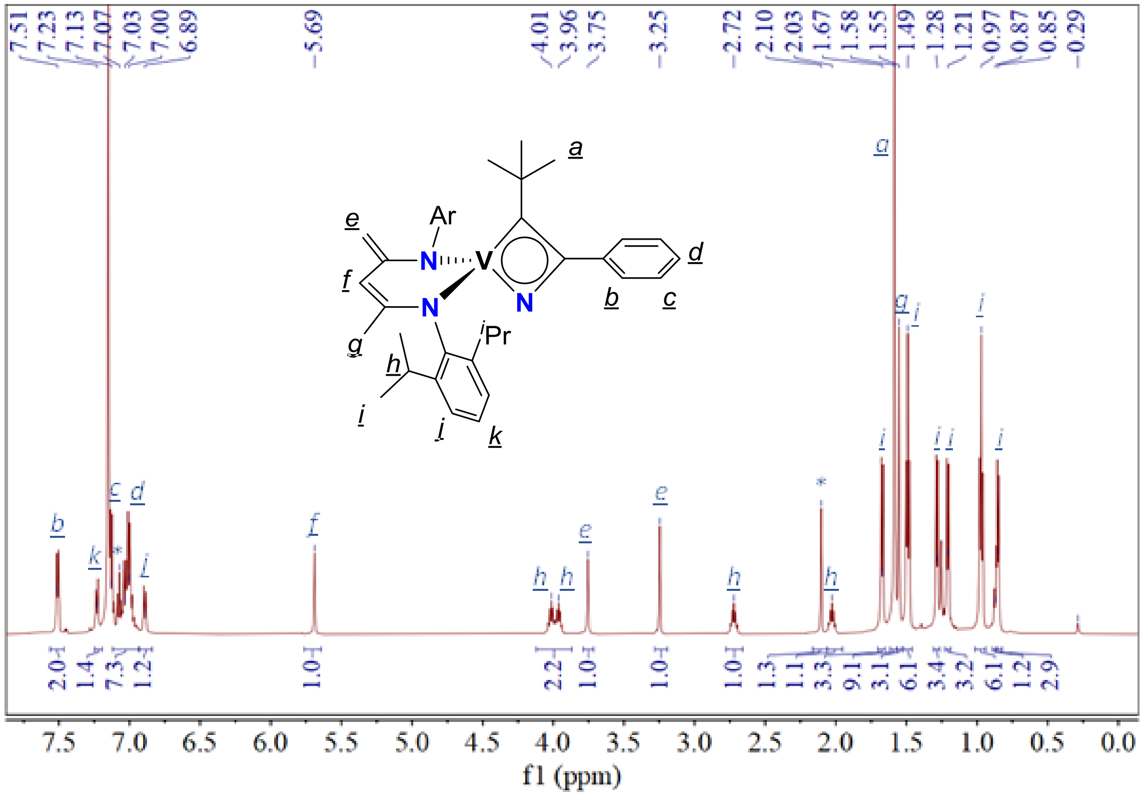

Figure S9: ^{1}H NMR spectrum of **2** (600 MHz, C_6D_6, 300K). The resonance assigned by * arises from residual toluene.

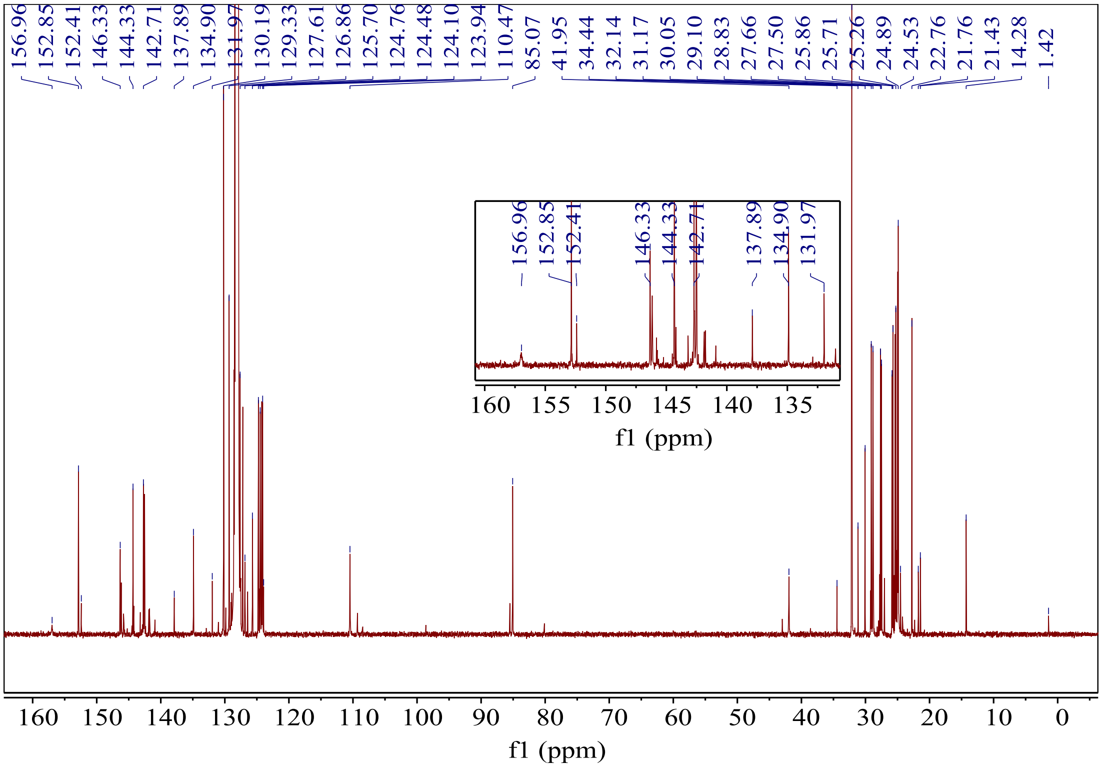

Figure S10: $^{13}C\{^{1}H\}$ NMR spectrum of **2** (151 MHz, C_6D_6, 300K)

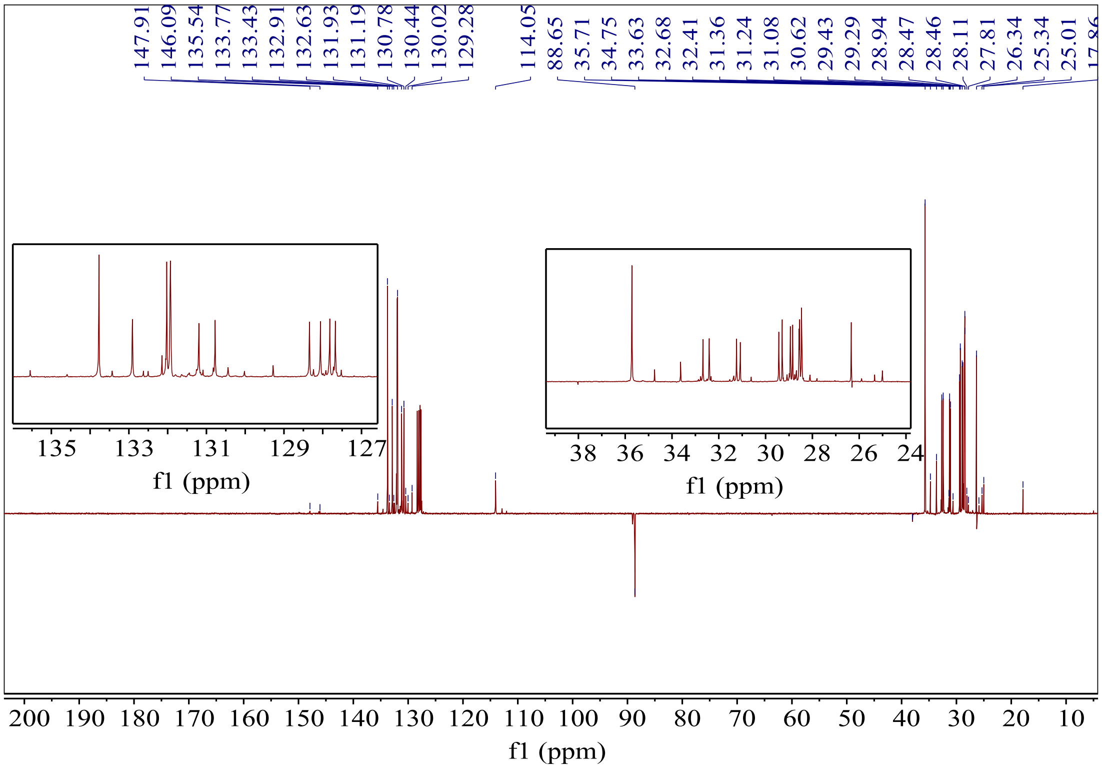

Figure S11: $^{13}C\{^{1}H\}$ DEPT–135 NMR spectrum of **2** (151 MHz, C_6D_6, 300K)

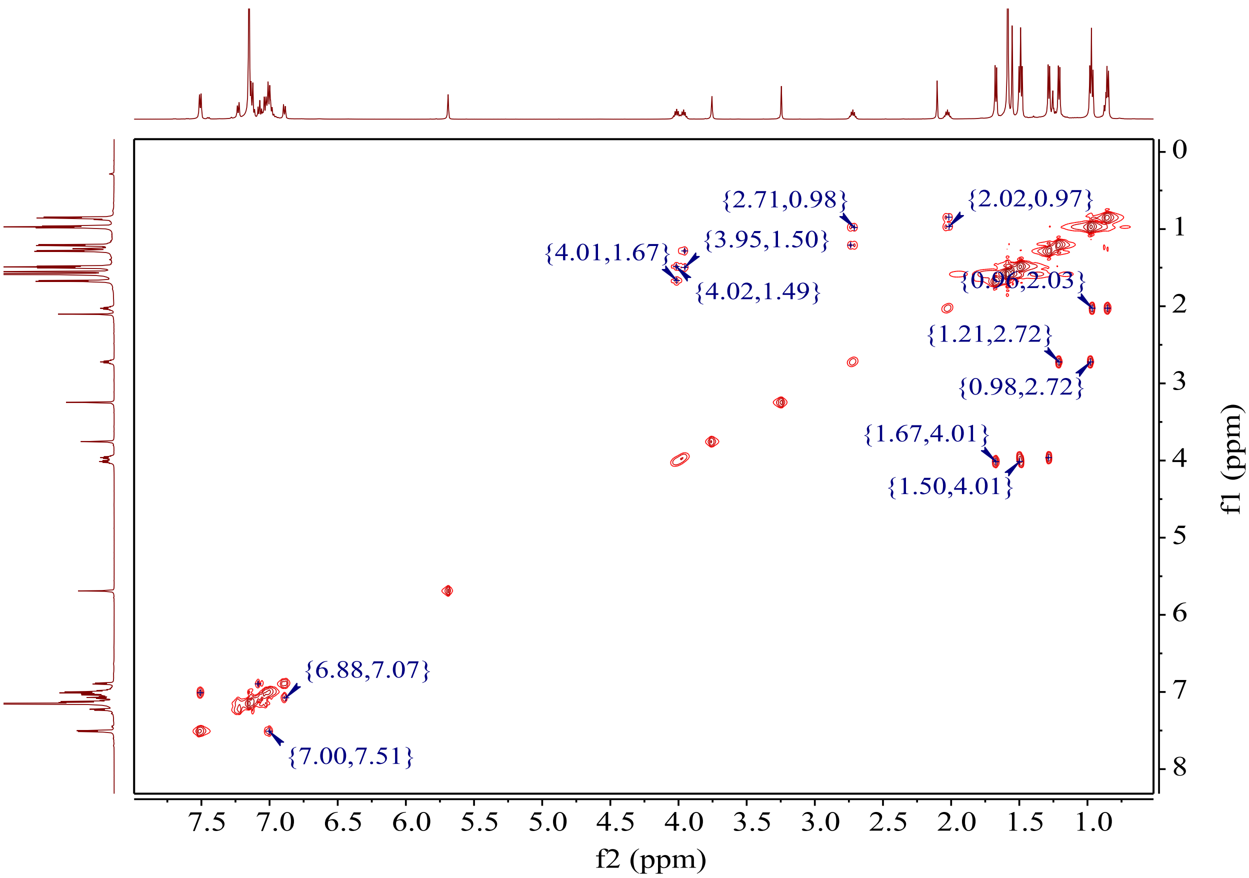

Figure S12: ^{1}H–^{1}H COSY NMR spectrum of **2** (600 MHz, C_6D_6, 300K)

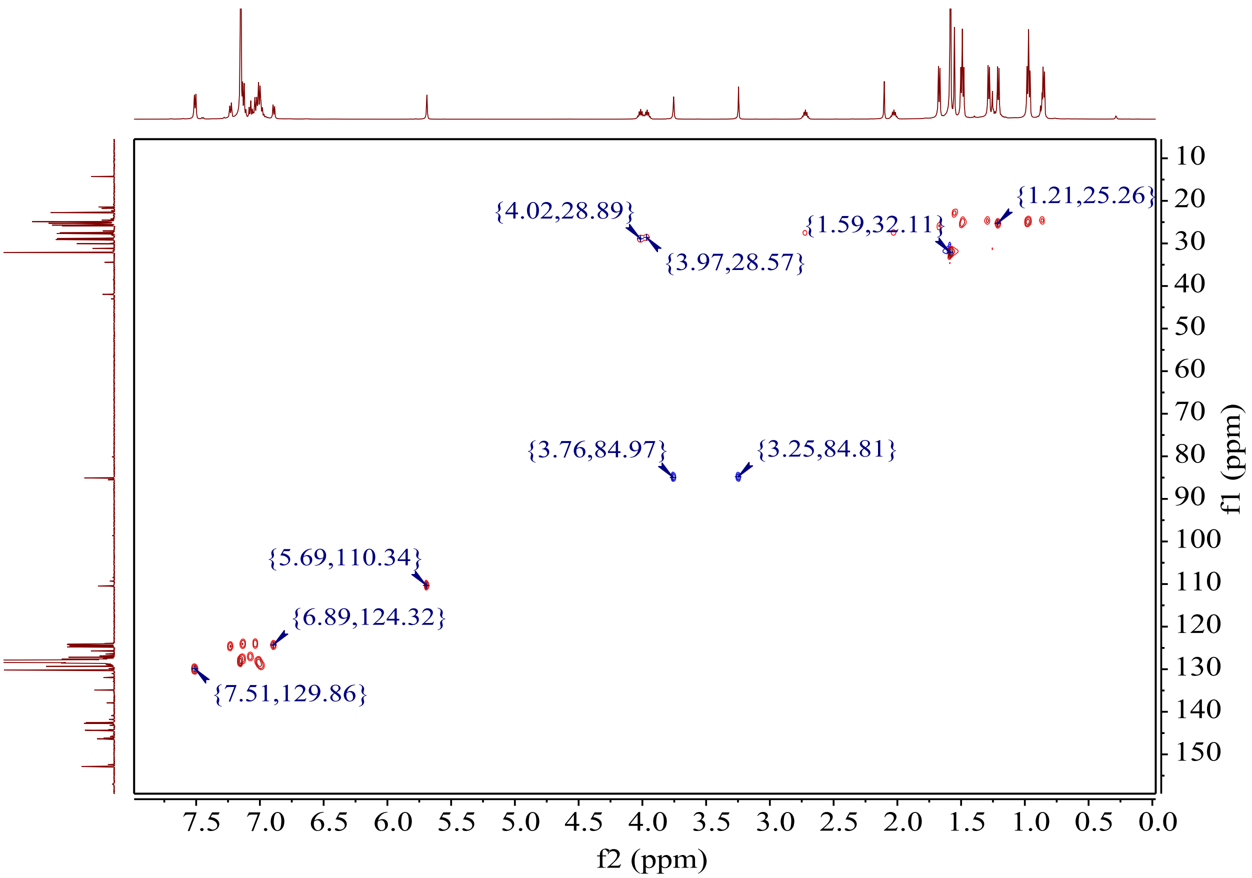

Figure S13: ^1H–^{13}C HSQC NMR spectrum of **2** ((600 MHz,151 MHz), C_6D_6, 300K)

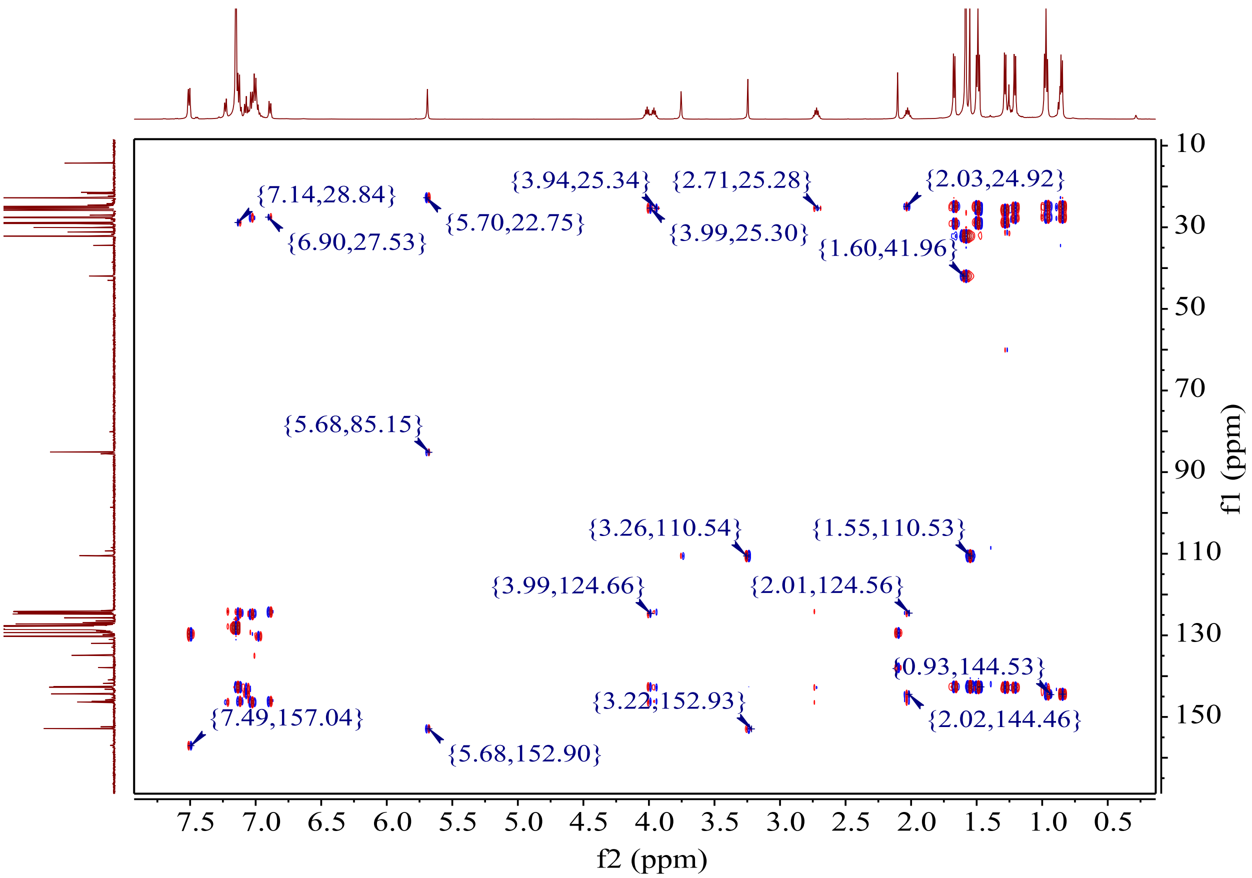

Figure S14: ^{1}H–^{13}C HMBC NMR spectrum of **2** ((600 MHz, 151 MHz), C_6D_6, 300K)

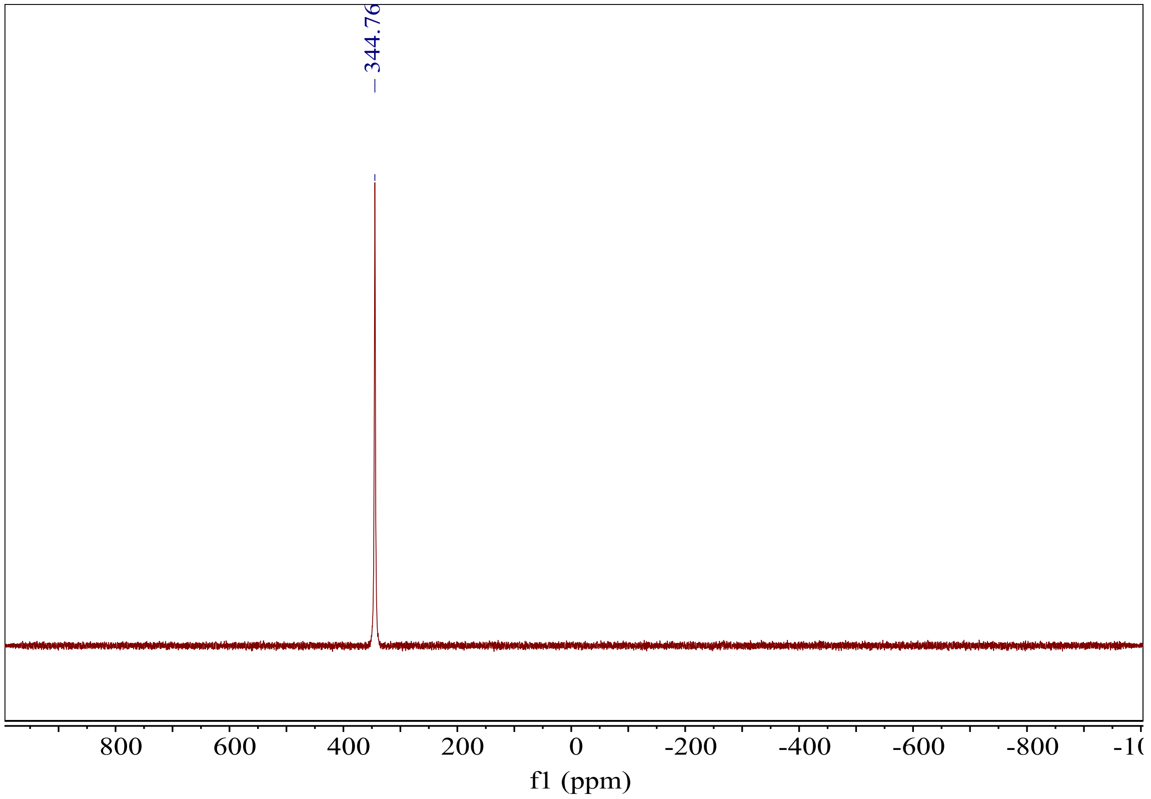

Figure S15: ^{51}V NMR spectrum of **2** (132 MHz, C_6D_6, 300K)

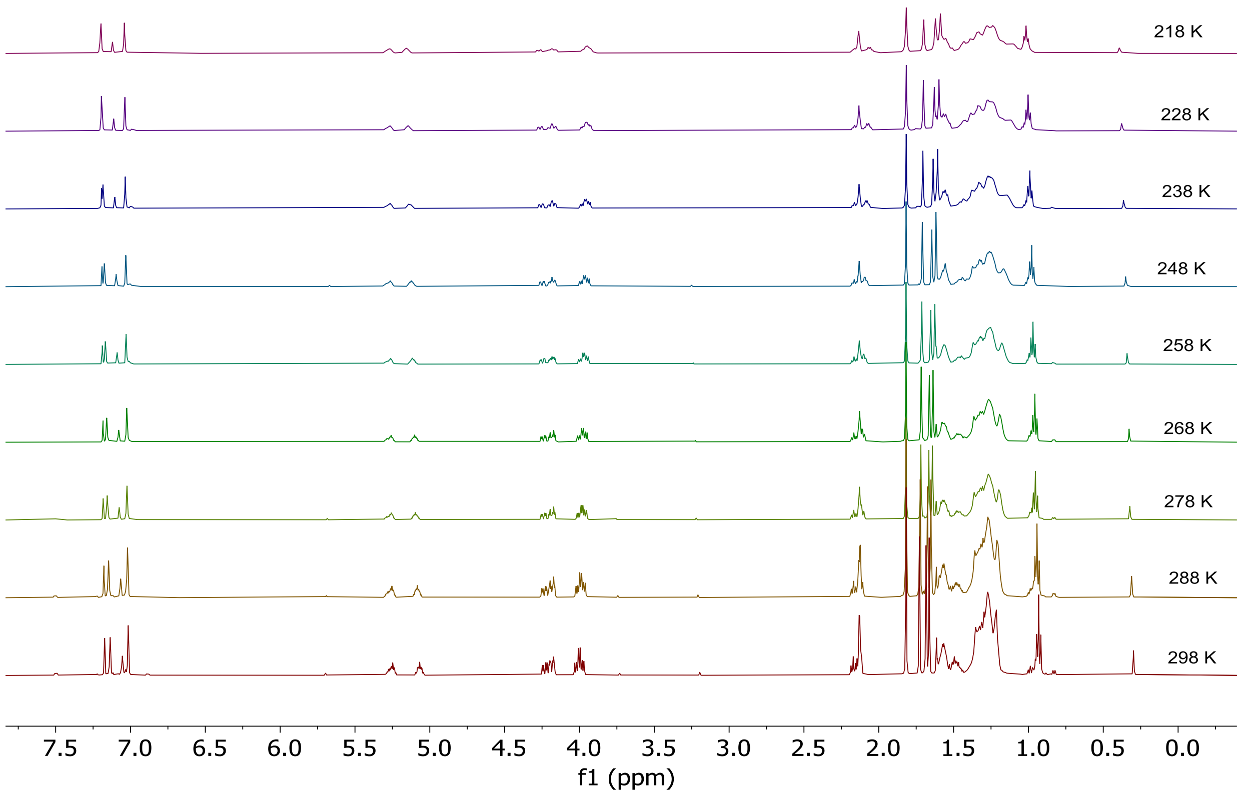

Figure S16: Stacked ^{1}H NMR spectrum of **2** (500 MHz, C_6D_6) at varible temperatures.

3.3: (dBDI)V(κ^2-***C***,***C***-^{t}Bu**C**P**C**Ad) (**3**)

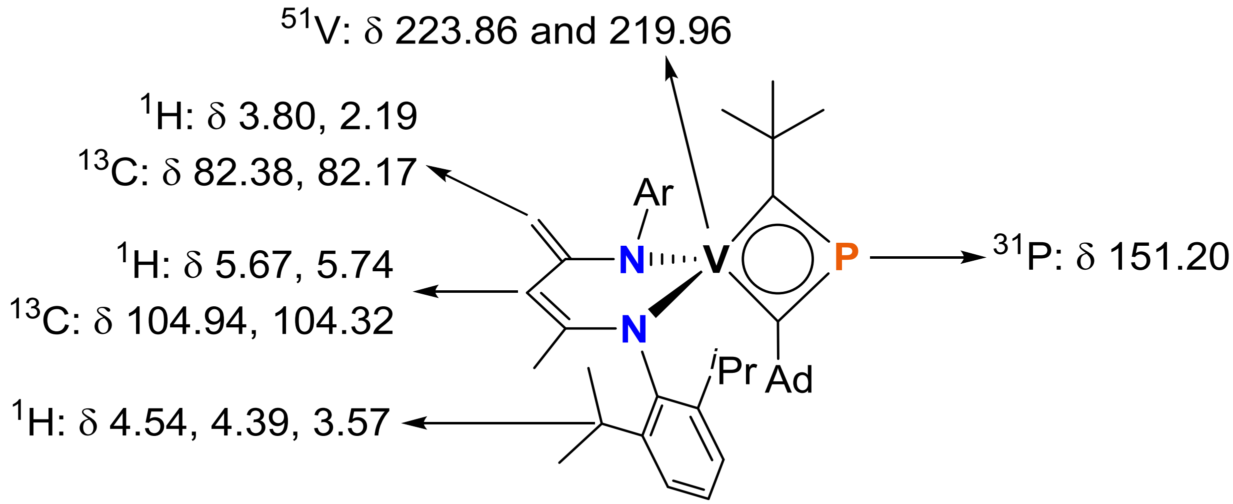

Figure S17: NMR assignments of **3**

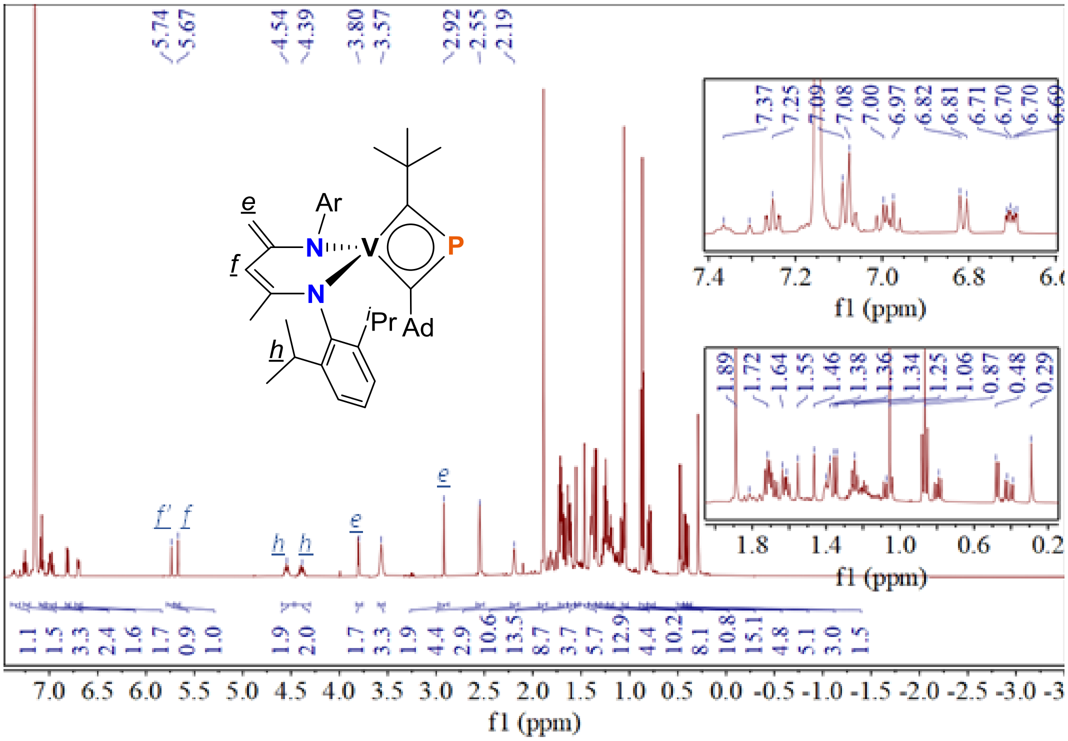

Figure S18: ^{1}H NMR spectrum of **3** (400 MHz, C_6D_6, 300K). Elaborate assignment of all resonances is unattainable given the extensive overlaps of peaks. Some P≡CAd impurity also remain in the sample.

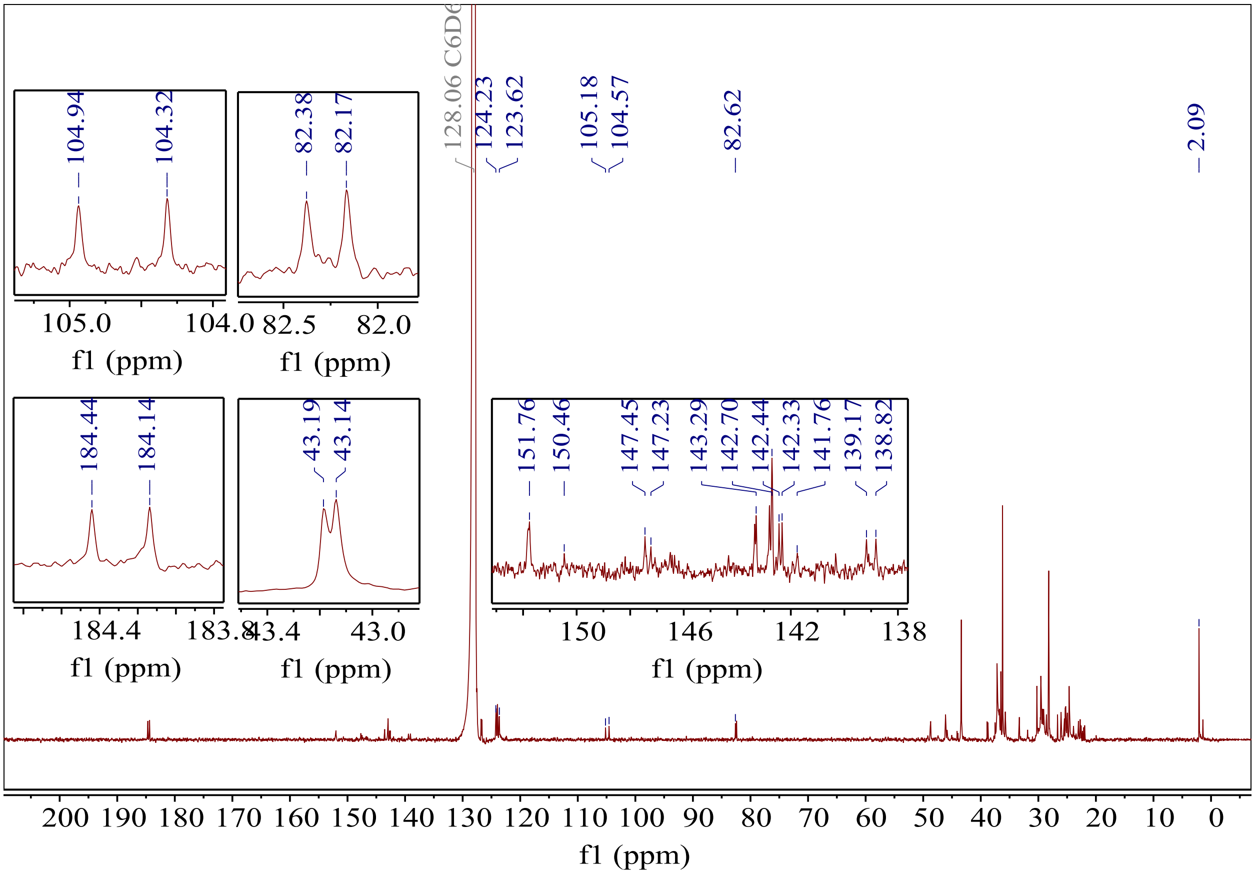

Figure S19: $^{13}C\{^{1}H\}$ NMR spectrum of **3** (126 MHz, C_6D_6, 300K). The insets show various resonances that appear as two sets of resonances close in chemical shift.

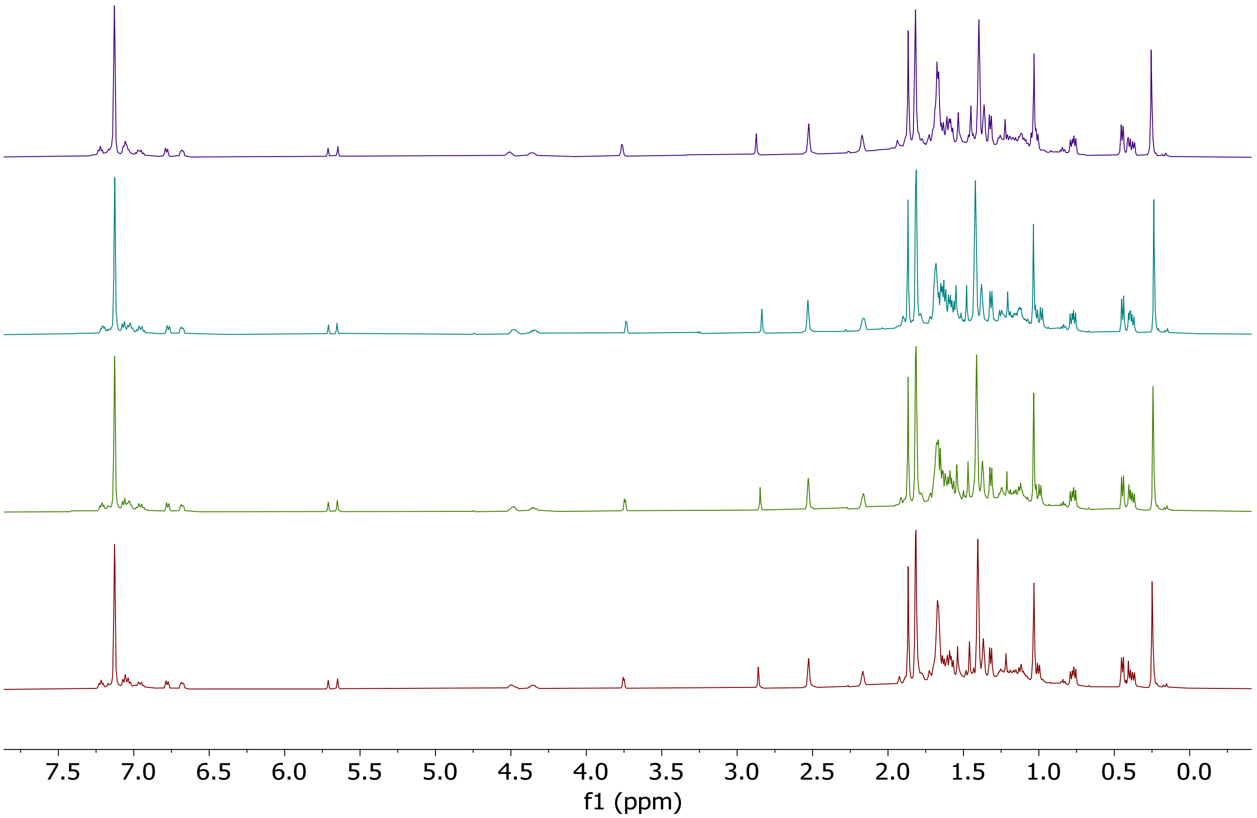

Figure S20: Stacked ^{1}H NMR spectrum of **3** (500 MHz, C_6D_6) at varible temperatures. 298K (red, bottom), 308K (green, 2nd from bottom), 318K (blue, 2nd from top), 328K (purple, top).

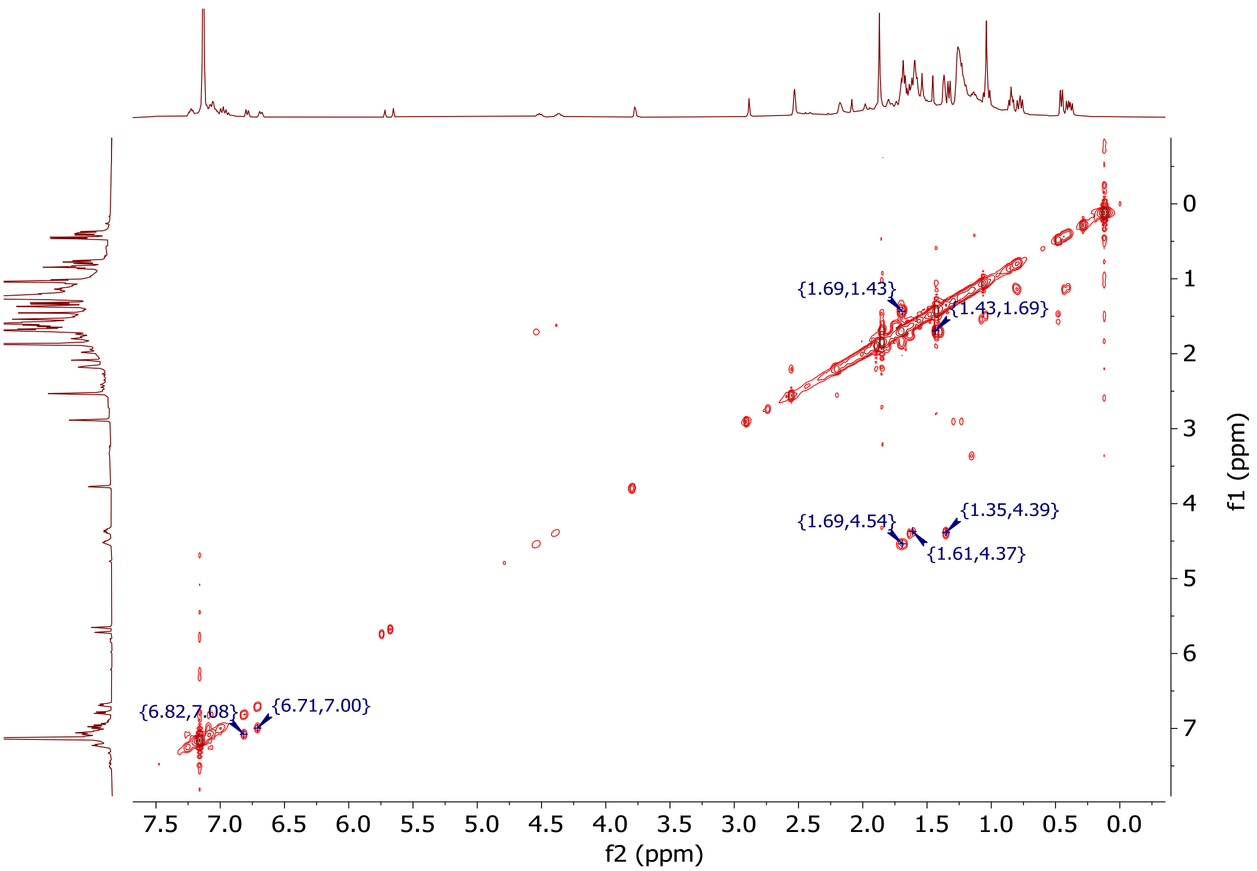

Figure S21: ^{1}H–^{1}H COSY NMR spectrum of **3** (500 MHz, C_6D_6, 300K)

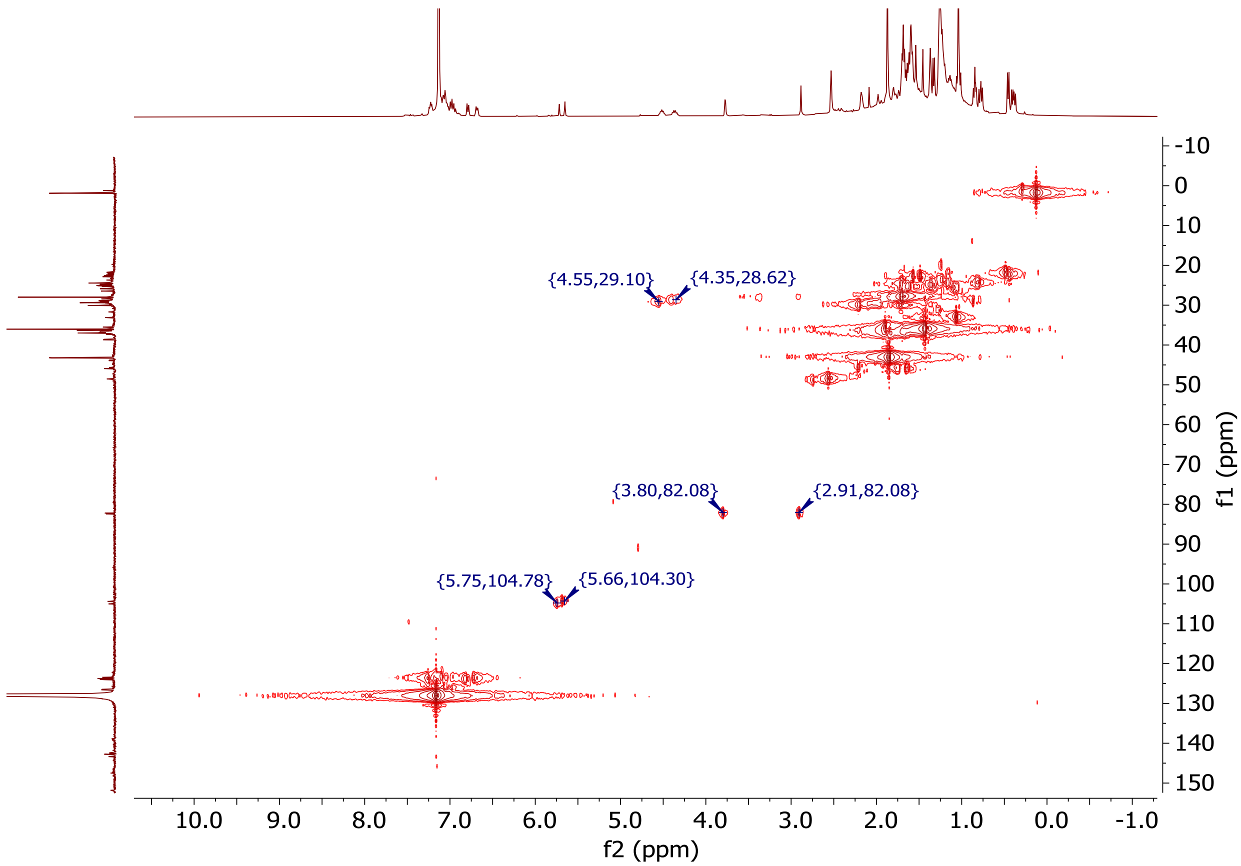

Figure S22: ^{1}H–^{13}C HSQC NMR spectrum of **3** ((600 MHz,151 MHz), C_6D_6, 300K)

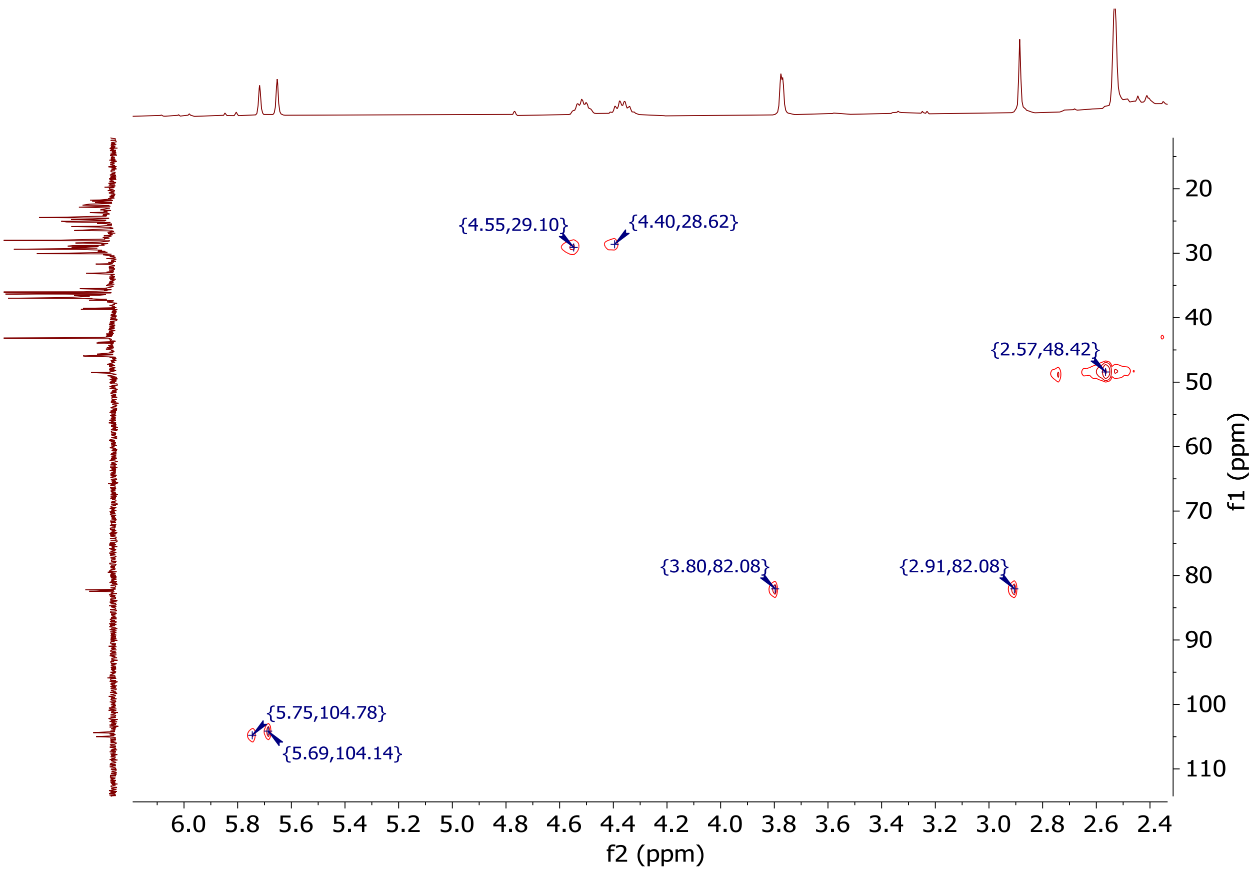

Figure S23: A zoomed in section of the ^{1}H–^{13}C HSQC NMR spectrum of **3** ((600 MHz,151 MHz), C_6D_6, 300K). This highlights the pair of inequivalent methylene (=CH_2) hydrogens at 3.80 and 2.91 ppm in the ^{1}H NMR are correlated to the same carbon resonance at 82.08 ppm. Additionally, the methine (C*H*) fragment of the bis anilido ligand appears as two distinct signals at (5.75 ppm, 104.78 ppm) and (5.69 ppm, 104.14 ppm) in this spectrum. This suggests that complex **3** exhibits two magnetically inequivalent but structurally similar species in solution.

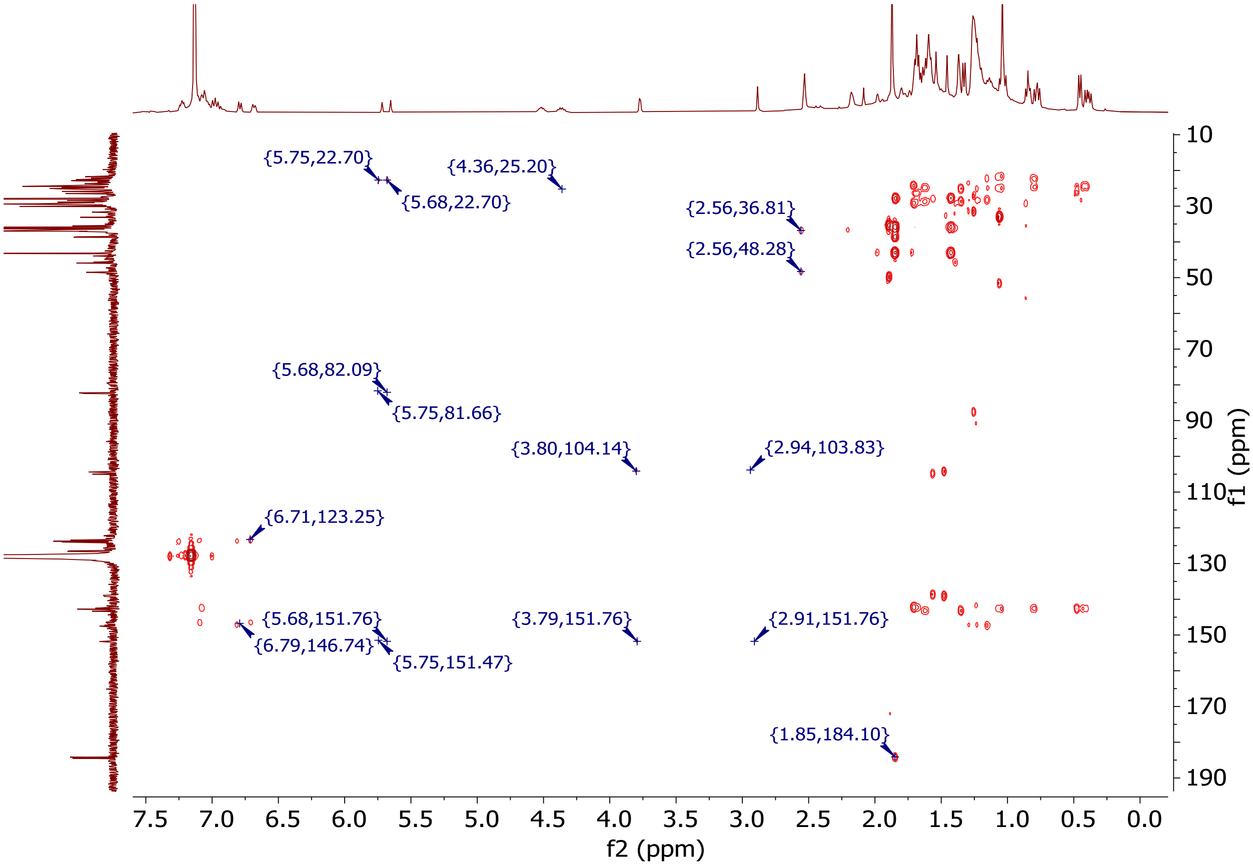

Figure S24: ^{1}H–^{13}C HMBC NMR spectrum of **3** ((600 MHz,151 MHz), C_6D_6, 300K)

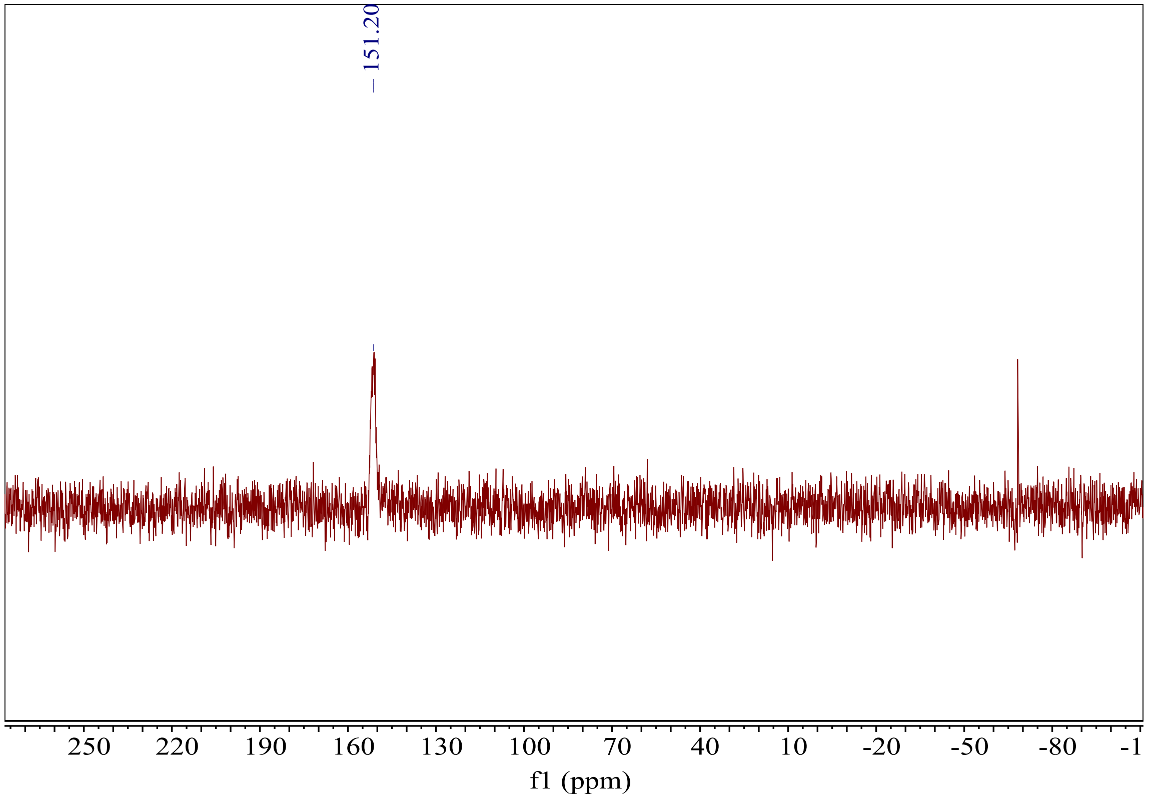

Figure S25: $^{31}P\{^1H\}$ NMR spectrum of **3** (202 MHz, C_6D_6, 300 K). The resonance at -68.21 pm corresponds to the internal standard (P≡CAd).

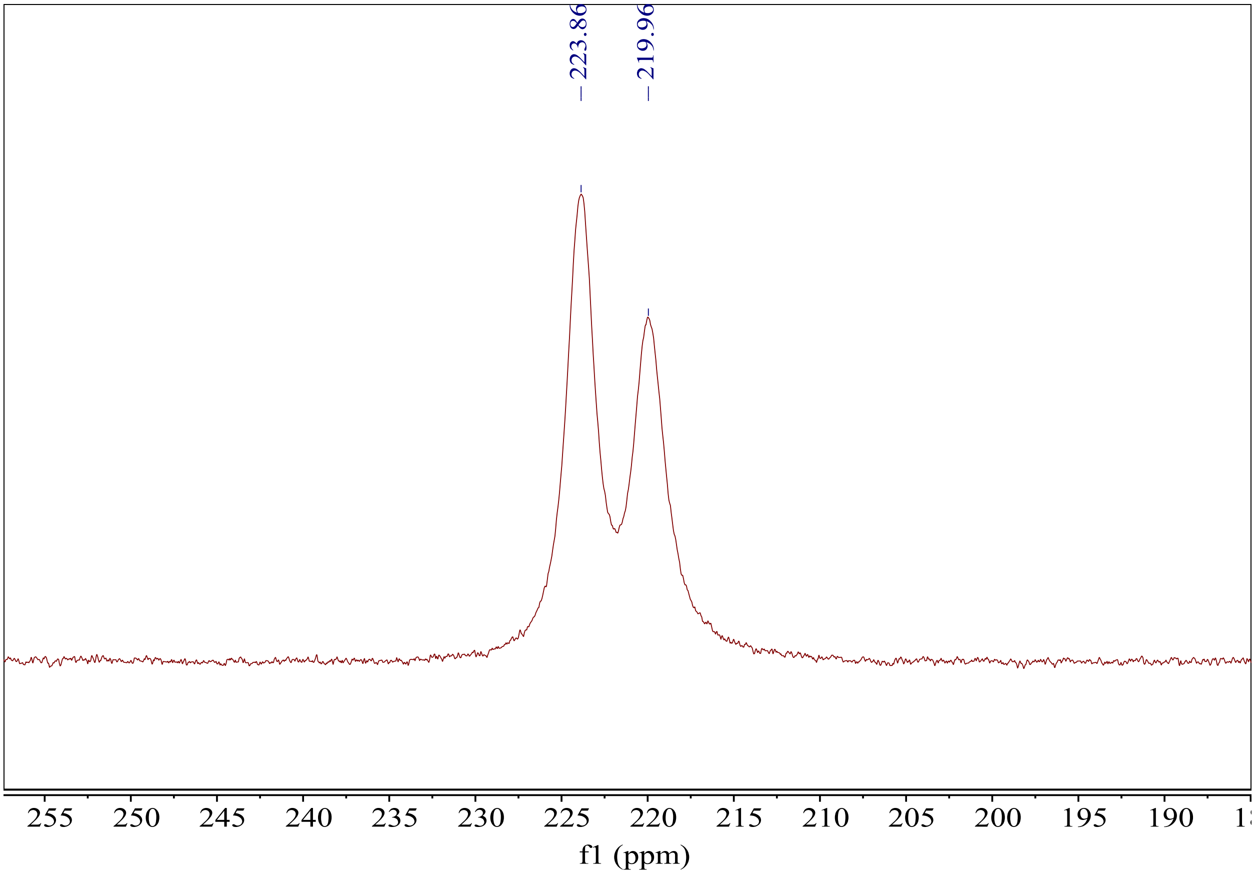

Figure S26: ^{51}V NMR spectrum of **3** (132 MHz, C_6D_6, 300 K)

4. IR Spectroscopic Data

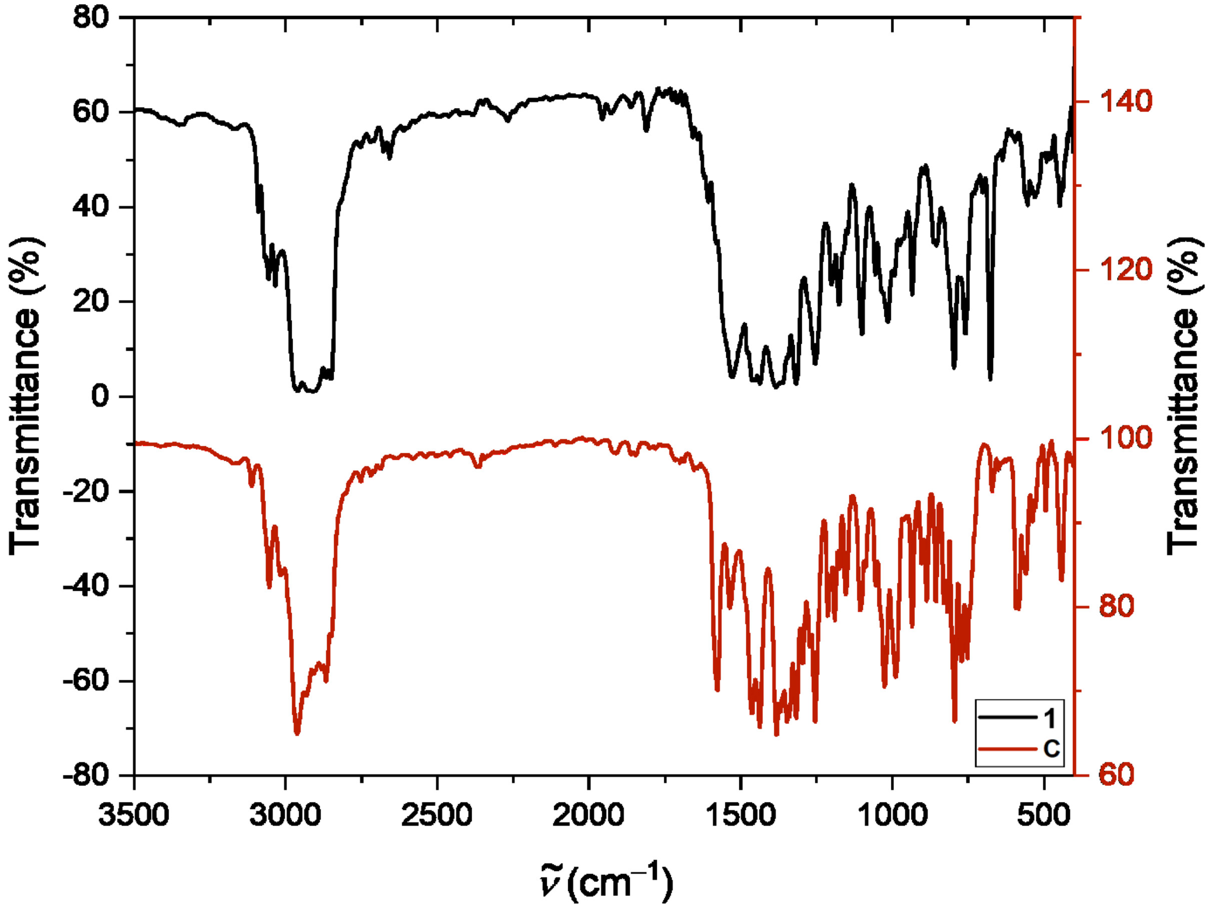

Figure S27: IR spectra of **1** *vs.* **C**

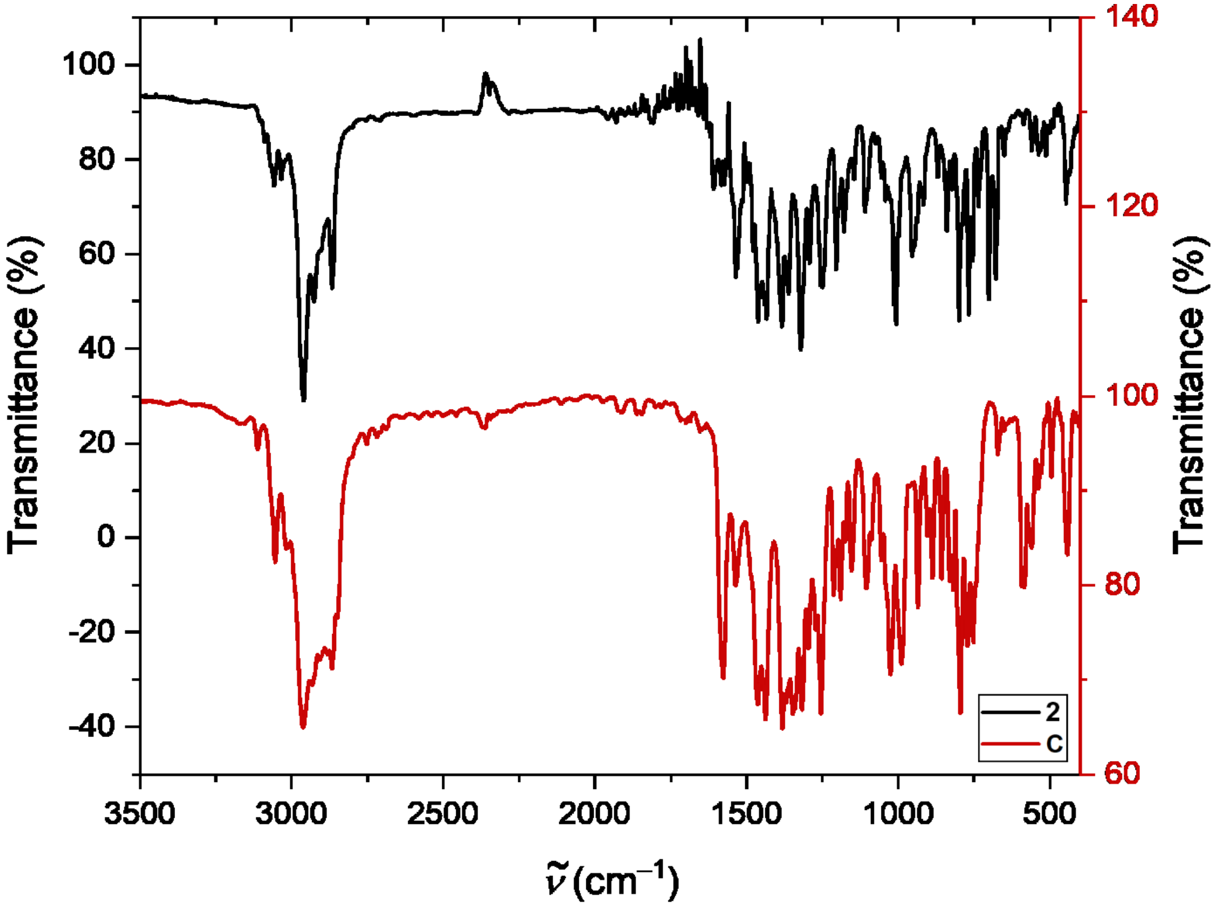

Figure S28: IR spectra of **2** *vs.* **C**

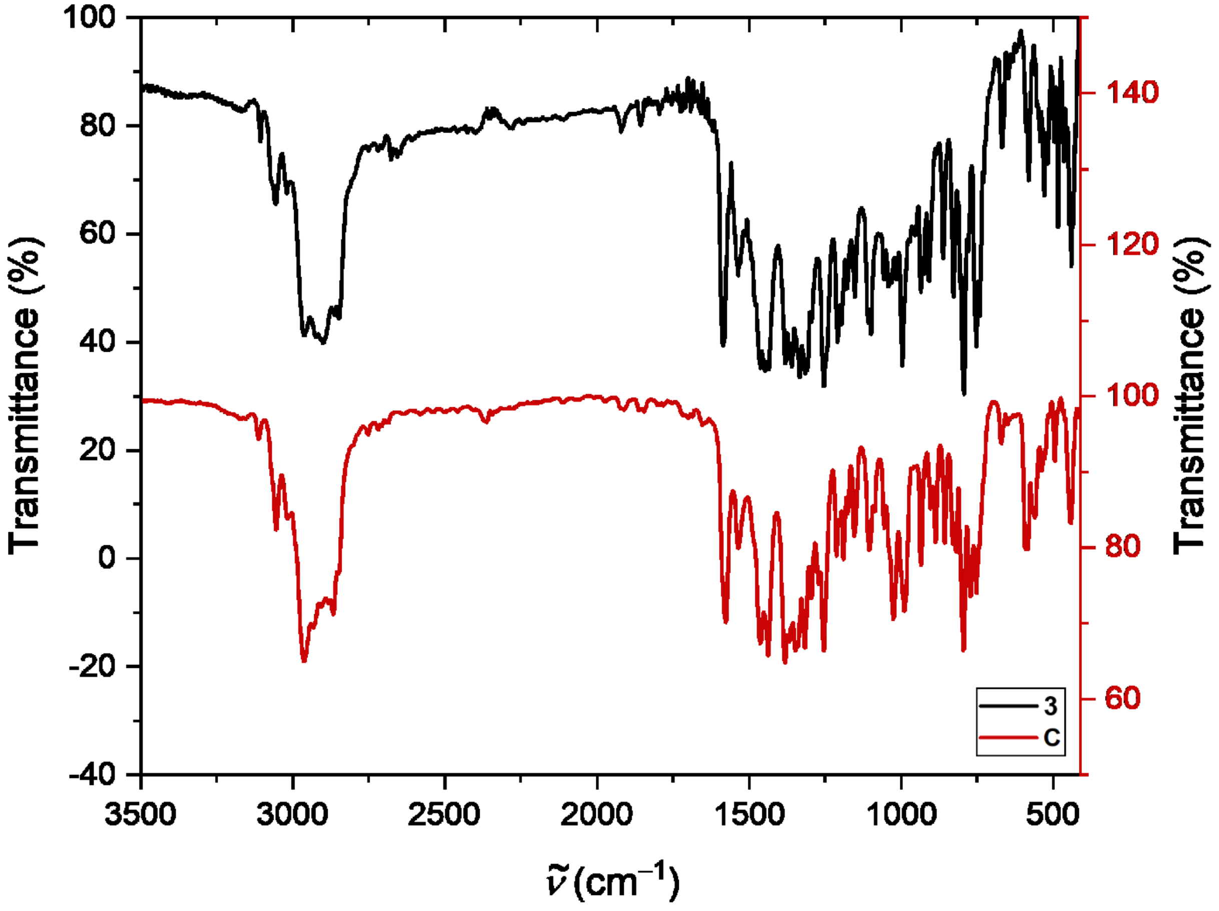

Figure S29: IR spectra of **3** *vs.* **C**

5. UV-Vis Spectroscopic Data

5.1: (dBDI)V(κ^2-***C***,***N***-^{t}Bu**C**C(Ad)**N**) (**1**)

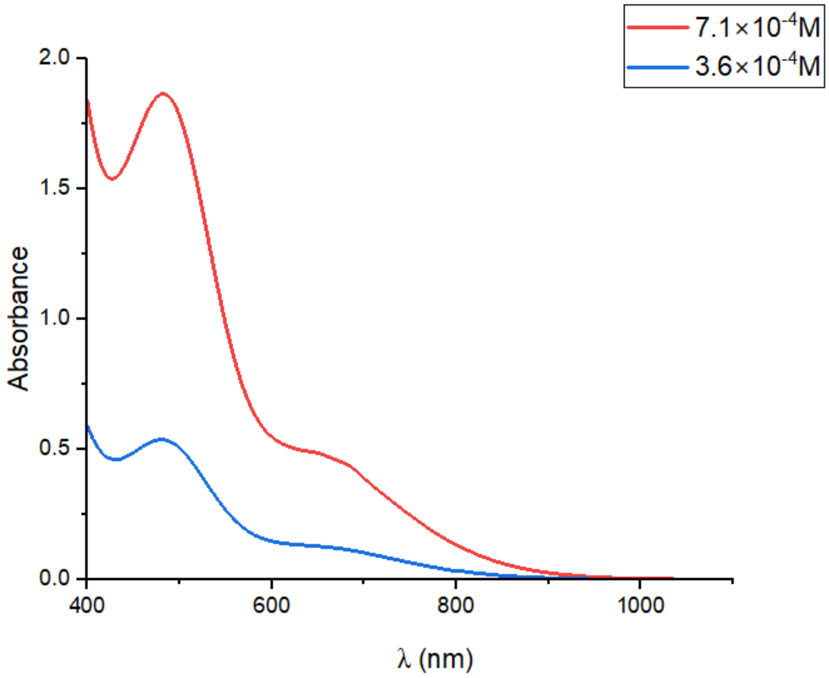

Figure S30: UV-Vis spectrum of **1** in pentane at various concentrations (red trace = 7.1 × 10^{-4} M and blue trace = 3.6 1 × 10^{-4} M).

5.2: (dBDI)V(κ[2]-***C***,***N***-[t]Bu**C**C(Ph)**N**) (**2**)

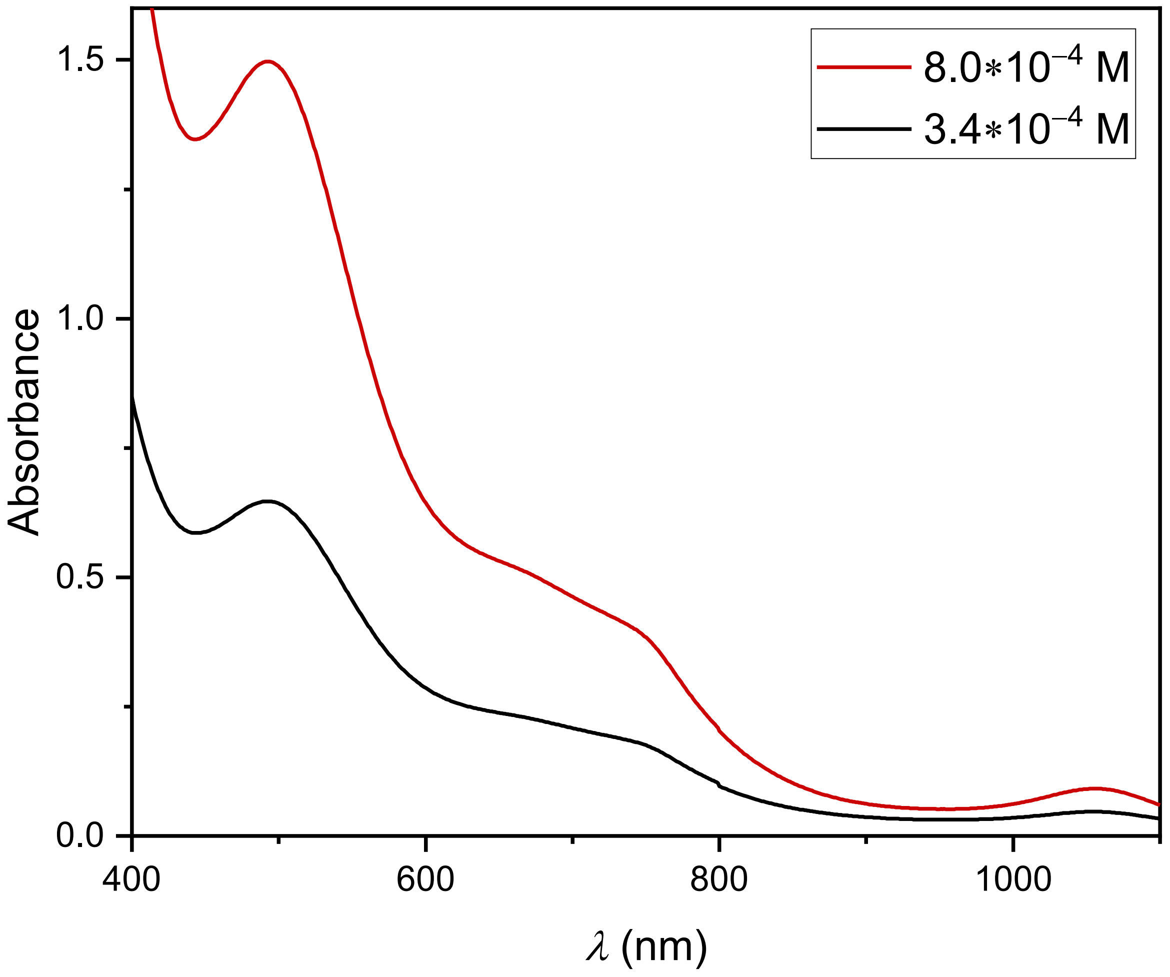

Figure S31: UV-Vis spectrum of **2** in pentane at various concentrations (red trace = 8.0 × 10^{-4} M and black trace = 3.4 × 10^{-4} M).

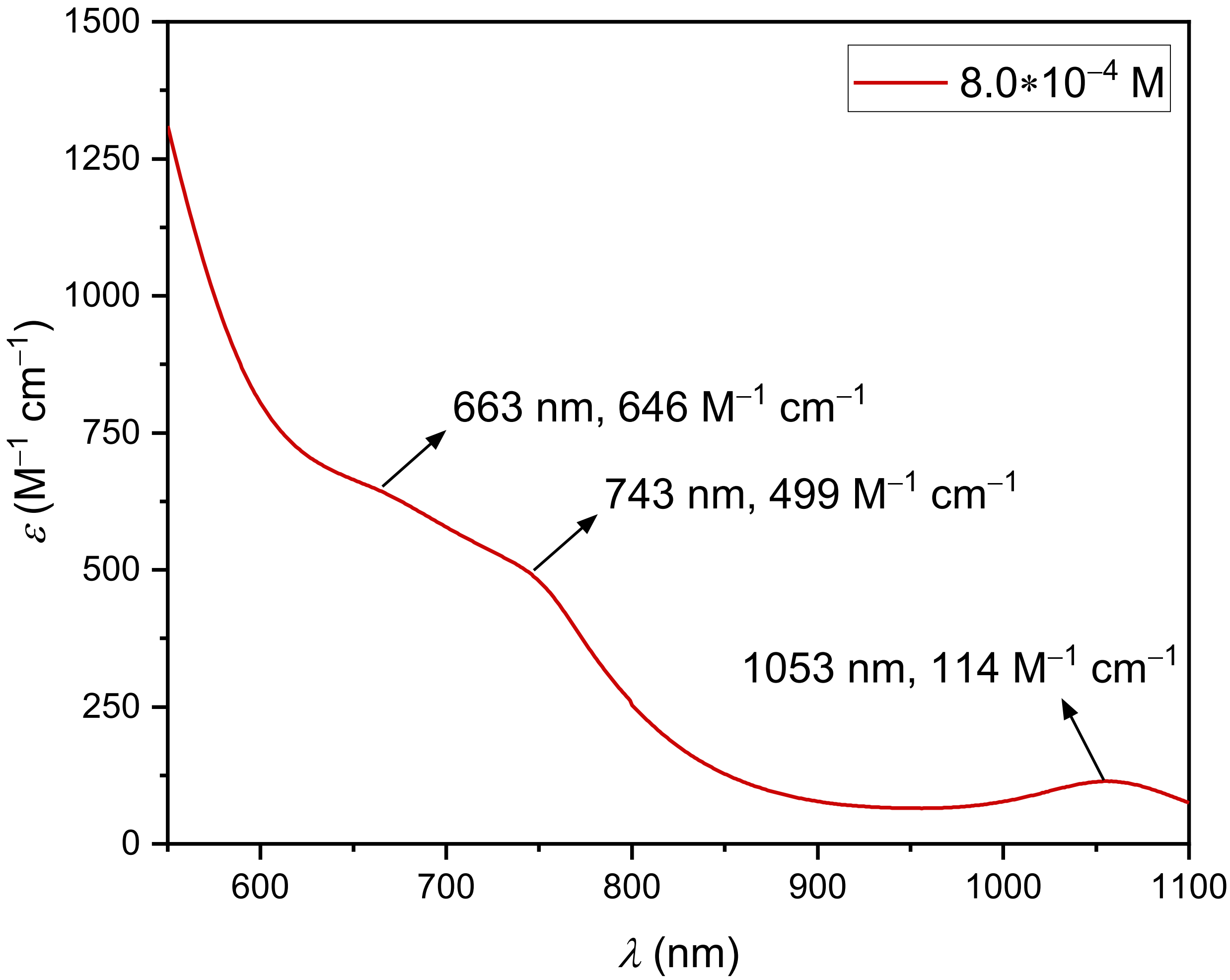

Figure S32: UV-Vis spectrum of a more concentrated sample of **2** in pentane (8.0 × 10^{-4} M).

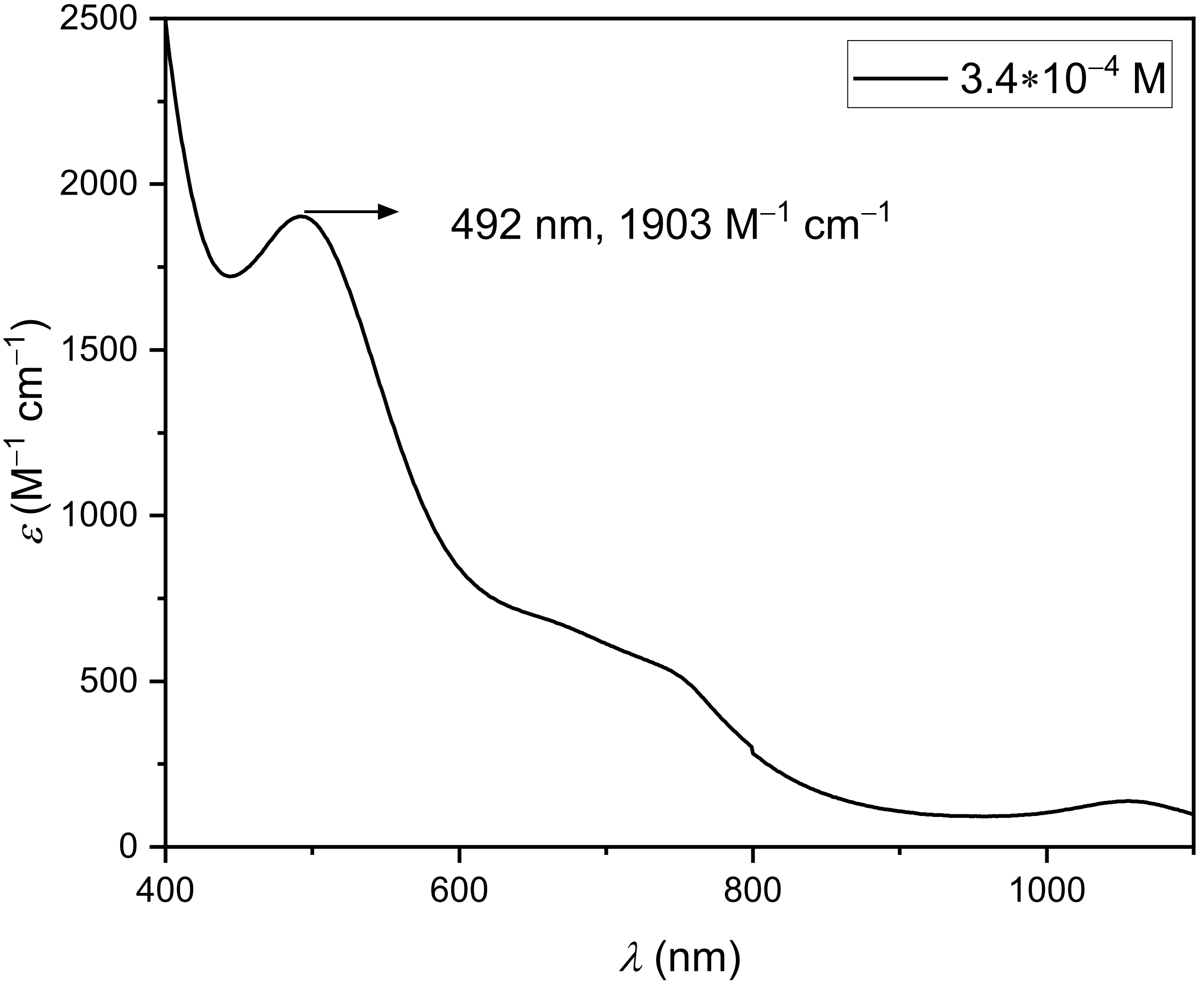

Figure S33: UV-Vis spectrum of a more dilute solution of **2** in pentane (3.4×10^{-4} M).

5.3: (dBDI)V(κ[2]-***C***,***C***-^{t}Bu**C**PCAd) (**3**)

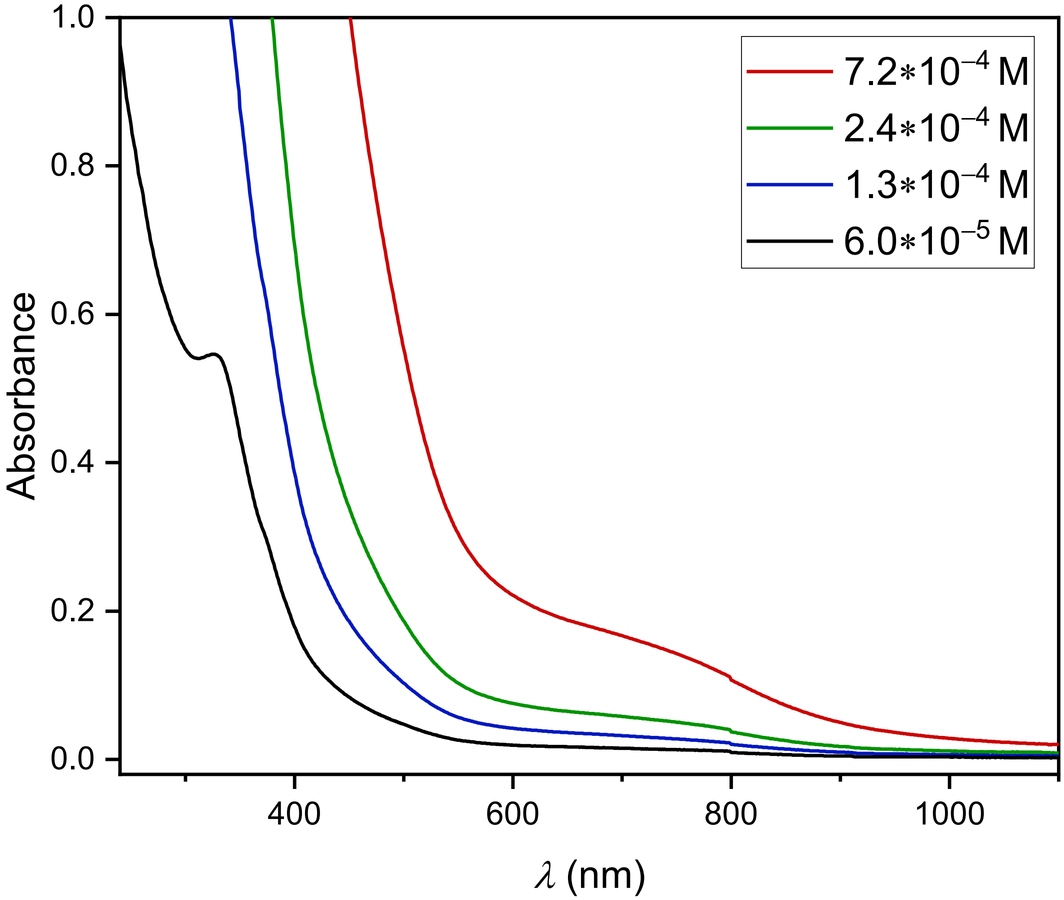

Figure S34: UV-Vis spectrum of **3** in pentane with various concentrations (red trace = 7.2 × 10^{-4} M, green trace = 2.4 × 10^{-4} M, blue trace = 1.3 × 10^{-4} M, and black trace = 6.0 × 10^{-5} M).

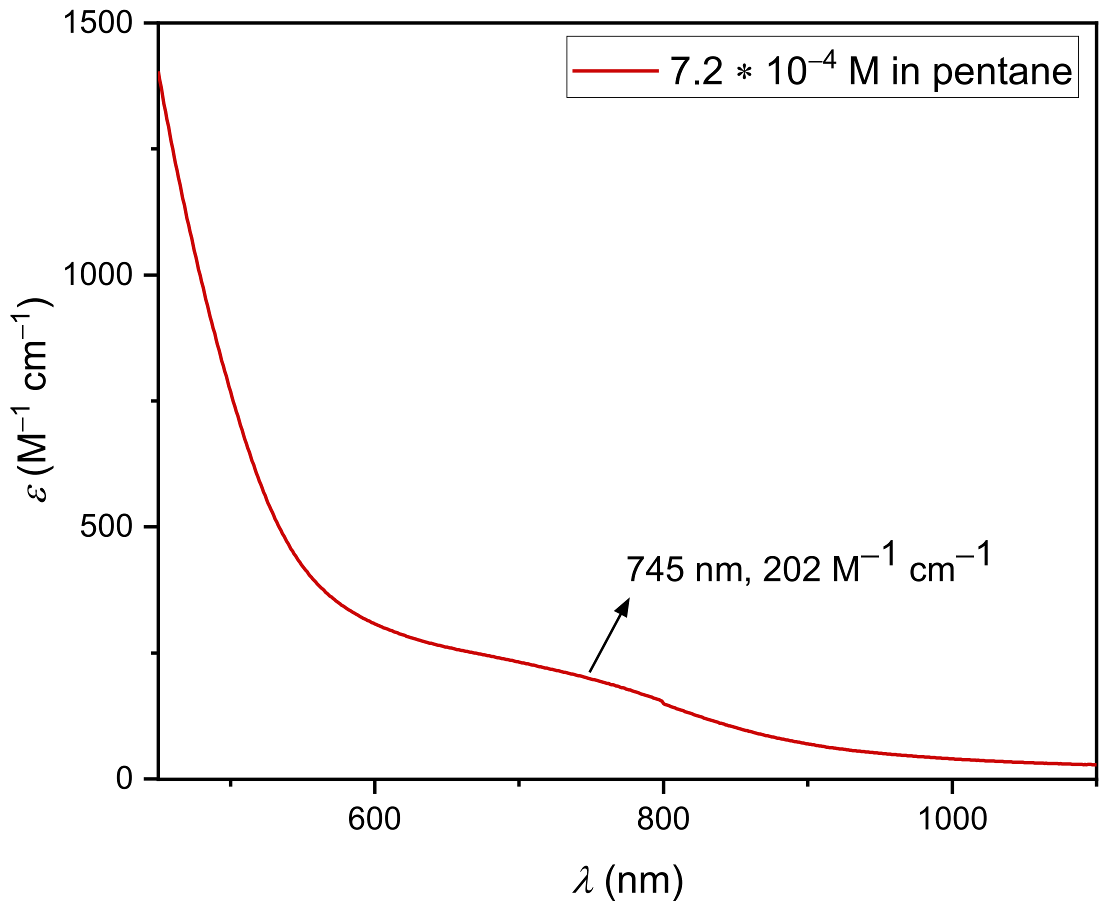

Figure S35: UV-Vis spectrum of concentrated sample of **3** in pentane (7.2×10^{-4} M).

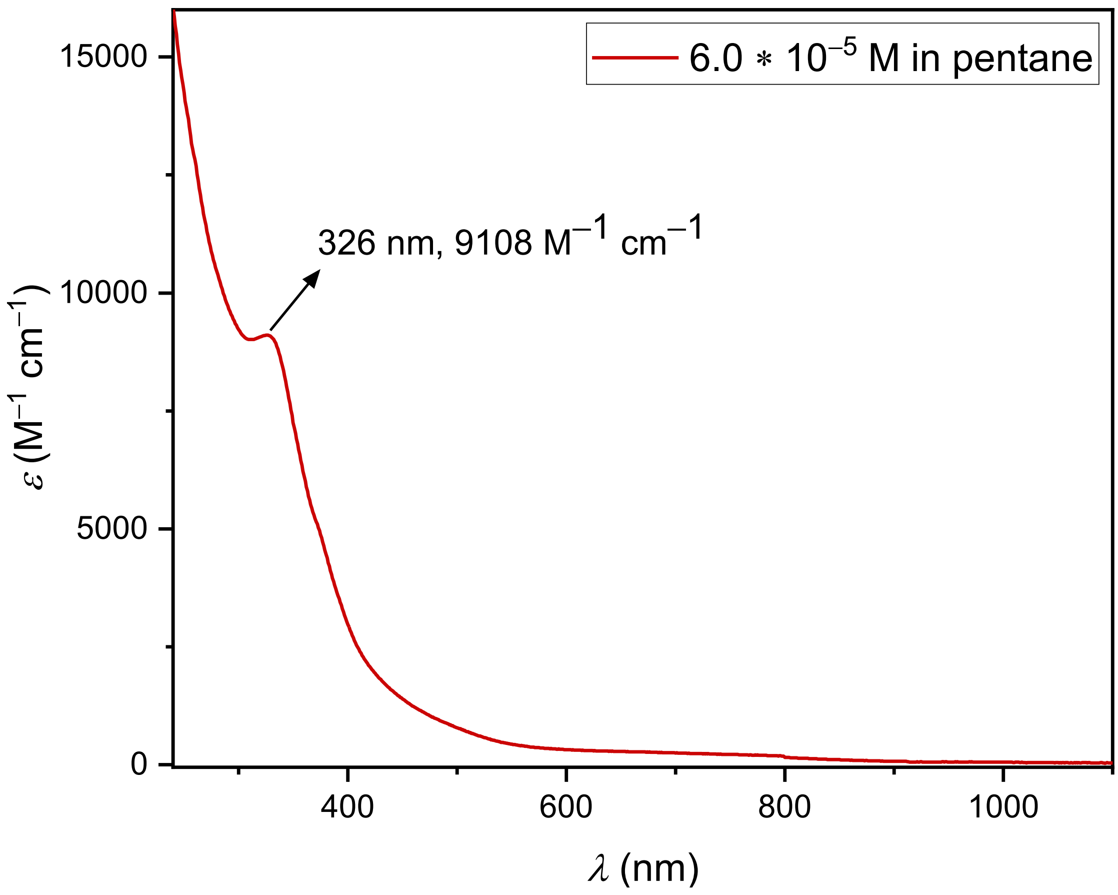

Figure S36: UV-Vis spectrum of dilute sample of **3** in pentane (6.0×10^{-5} M).

6. Computational Data

6.1. Functional Benchmark Study

Prior to conducting the electronic structure analysis, we performed a functional benchmark study on the structural parameters of three complexes to identify the most suitable computational methods for accurately describing the bonding nature of metallacycles. To assess the influence of the theoretical background and Hartree Fock exchange percentage of functionals, we compared the optimized structures of the three complexes using the PBE-D3(BJ),[5-7] B3LYP-D3(BJ)[12-15] and M06-2X[16] functionals (Table S1). The mean absolute error (MAE) and root mean squared error (RMSE) of atomic distances, when compared with X-ray crystal structures, showed only marginal differences between the observed and predicted geometries. Therefore, to maximize computational efficiency, we selected the PBE-D3(BJ) functional for the electronic structure analysis.

Table S1: MAE and RMSE of key atomic distances in **2-4** computed with various functionals.

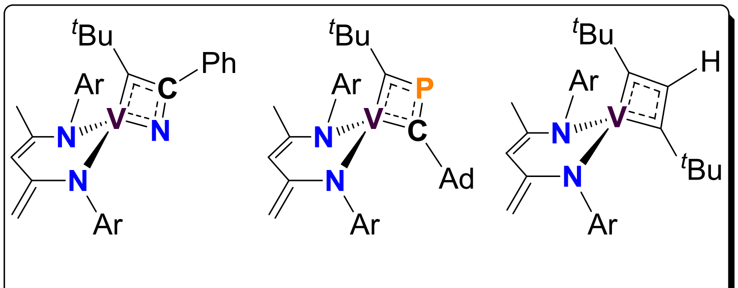

2 **3** **4**

	**Functional**	**MAE (Å)**	**RMSE (Å)**
**2**	PBE-D3(BJ)	0.012	0.015
	B3LYP-D3(BJ)	0.010	0.012
	M06-2X	0.021	0.025
**3**	PBE-D3(BJ)	0.014	0.018
	B3LYP-D3(BJ)	0.016	0.020
	M06-2X	0.029	0.034
**4**	PBE-D3(BJ)	0.030	0.033
	B3LYP-D3(BJ)	0.029	0.034

6.2. Molecular Orbital Correlation Diagram between V and dBDI

Figure S37 illustrates the molecular orbital correlation diagram between the metal center and the dBDI ligands. The minimal structural differences among these ligands in the complexes result in similar bonding interactions with the metal center, which are reflected in their comparable shapes and energy levels.

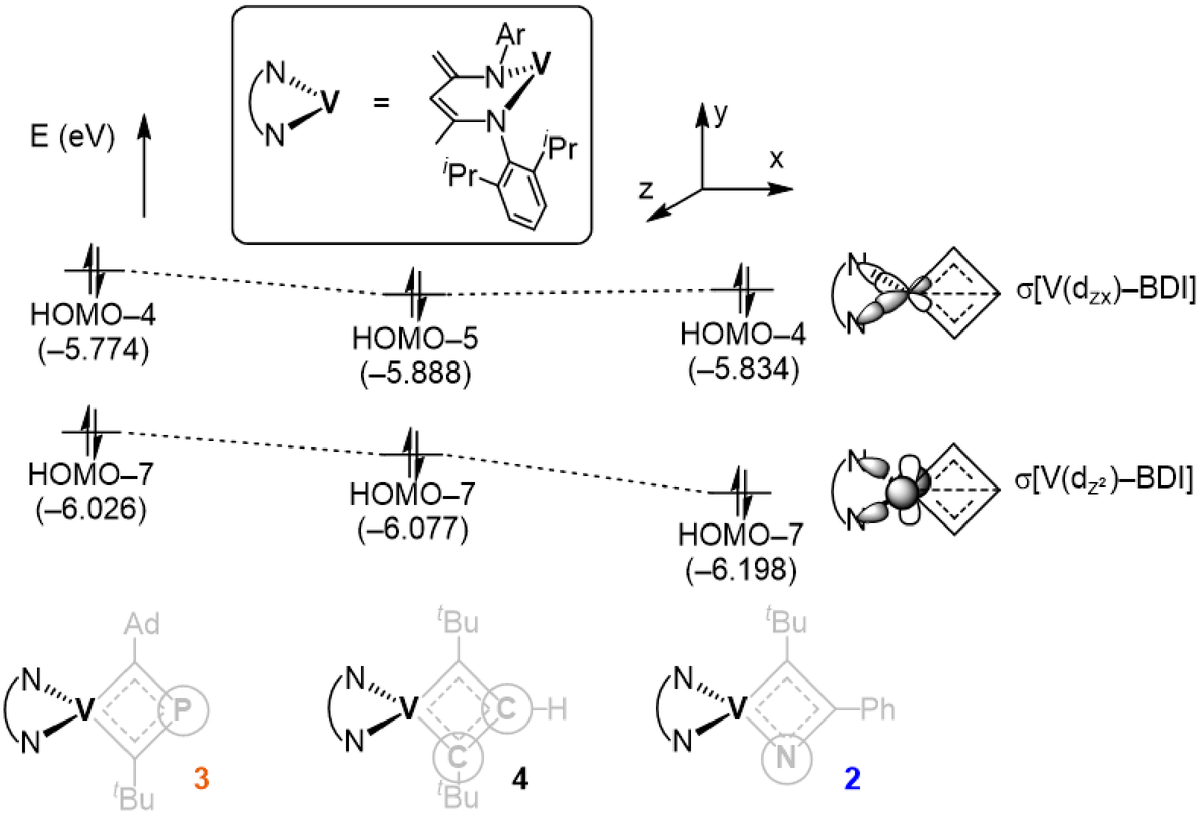

Figure S37: Molecular orbitals of **2**-**4** interacting between the metal center and the dBDI ligands

We identified that in complex **4**, HOMO-5, LUMO+1, and LUMO+15 involve interactions between the dxz orbital of the vanadium center (Vd_{xz}), the non-bonding orbital of the BDI ligand (BDInb), and the π* orbital of the cyclobutadienyl ligand (Lπ*). HOMO-5 represents the V–BDI σ-bond, while the high-lying Lπ* has a negligible contribution. LUMO+1 displays π-bonding interaction between Vd_{xz} and Lπ*, but the out-of-phase combination with BDInb renders this molecular orbital unoccupied. Finally, the π*(V–L) interaction is clearly identified in LUMO+15, although the contribution from the low-lying BDInb is negligible. These calculations are illustrated in **Figure S38**.

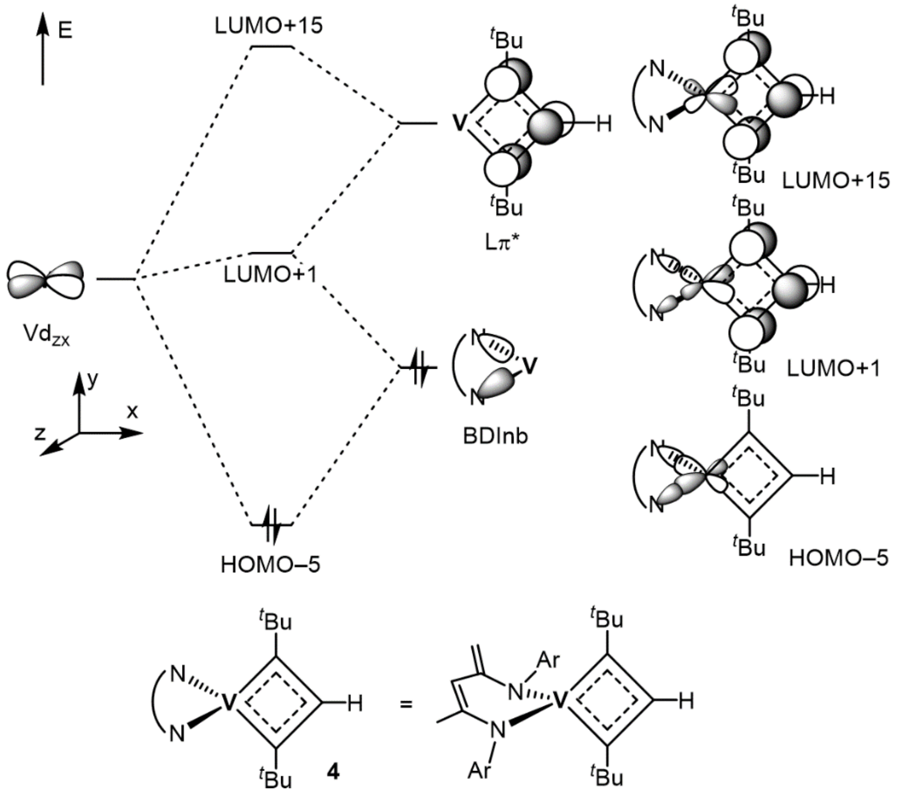

Figure S38: Molecular orbital correlation diagram of **4** describing the interactions between Vd_{xz}, BDInb, and Lπ*.

6.3. Mayer Bond Order Analysis

Mayer bond order[17] is a widely used method for determining bond order between atoms, and we initially applied it to identify the most probable resonance structure of each complex. **Figure S39** presents the Mayer bond orders between atoms in the MCBD scaffolds. However, we observed that the total bond order at the beta-position tends to be underestimated, conflicting with our chemical intuition. For example, the beta-carbon of complex **4** exhibits a total bond order of 3.47 (C−H calculated bond order in 0.85), which is significantly lower than the ideal value of 4.00. Therefore, we chose to present the Nalewajski-Mrozek bond order[18] in the main manuscript instead, as it offers clearer insights into bond orders and resonance structures.

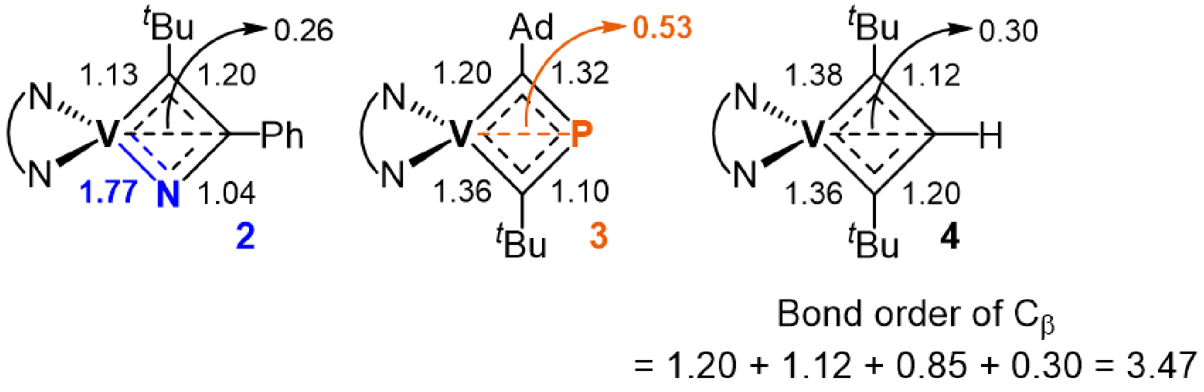

Figure S39: Mayer bond order between atoms in the metallacyclobutadienes.

6.4. QTAIM and ELF Analysis

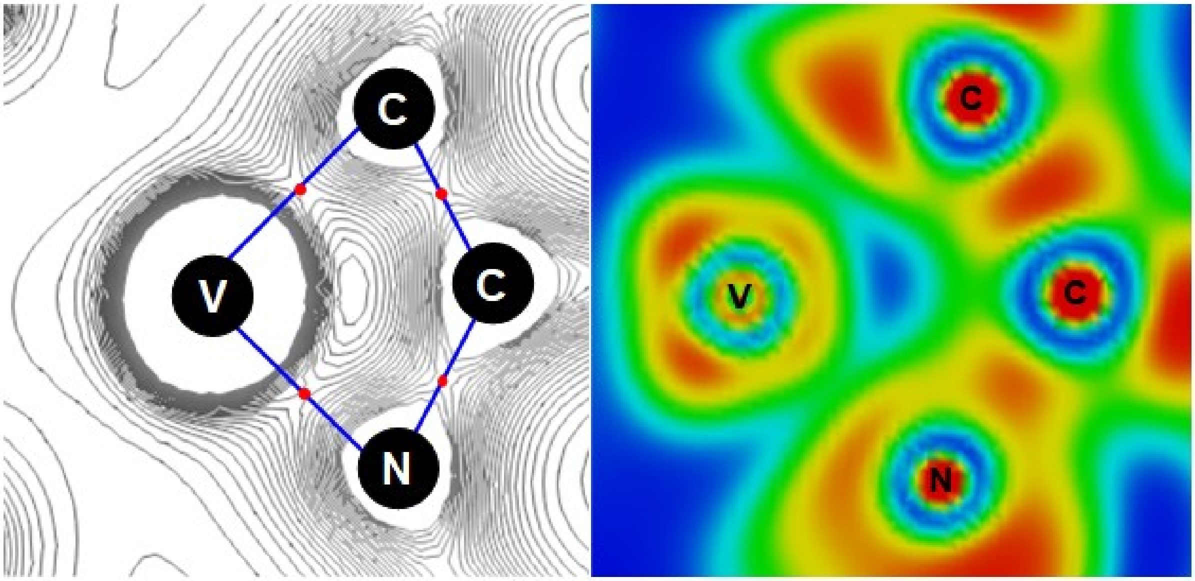

Figure S40: (Left) QTAIM plot and (Right) ELF plot of the MCBD scaffold in complex **2**. Blue lines indicate bond path points, and red dots indicate bond critical points.

Figure S40 illustrates the QTAIM and ELF plots of complex **2**. The QTAIM analysis identifies bond critical points positioned in the middle of each bond, indicating regions of electron density concentration. The ELF plot further reveals that the V–C, C–C and C–N bonds exhibit high covalent character, as evidenced by the significant electron density at their critical points. In contrast, the V–N bond displays relatively lower electron localization at its critical point, underscoring the ionic nature of the bond due to the significant electronegativity difference between vanadium and nitrogen.

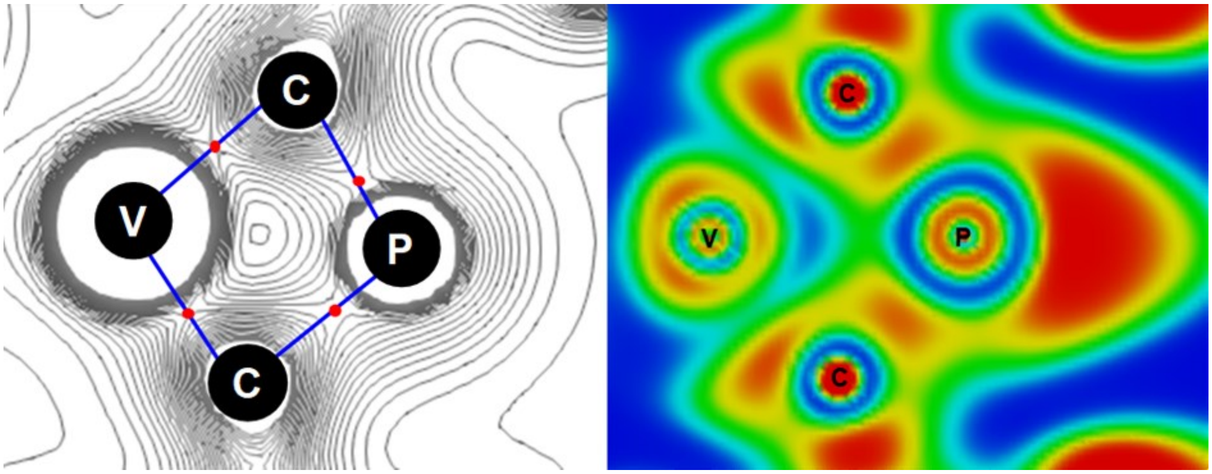

Figure S41: (Left) QTAIM plot and (Right) ELF plot of the MCBD scaffold in complex **3**. Blue lines indicate bond path points, and red dots indicate bond critical points.

Figure S41 illustrates the QTAIM and ELF plots of complex **3**. In the QTAIM analysis, the bond critical points for the V–C bonds are positioned in the middle of each bond, indicating balanced electron density. For the C–P bonds, however, the critical points are located closer to the phosphorus atom, reflecting the electron density's shift towards phosphorus. As discussed in the main text, the ELF plot distinctly highlights the lone pair of the phosphorus atom, further emphasizing its presence and contribution to the bonding environment in complex **3**.

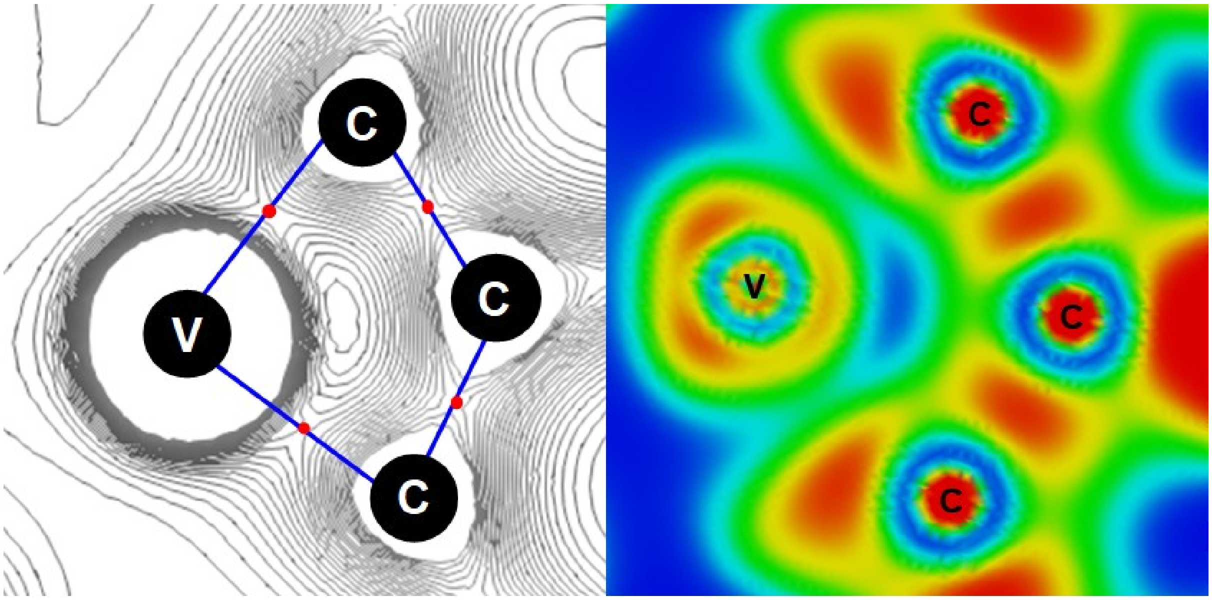

Figure S42: (Left) QTAIM plot and (Right) ELF plot of the MCBD scaffold in complex **4**. Blue lines indicate bond path points, and red dots indicate bond critical points.

Figure S42 illustrates the QTAIM and ELF plots of complex **4**. Owing to the symmetric bonding nature of this complex, both QTAIM and ELF plots display highly symmetric contour patterns, with bond critical points centrally located within each bond.

7. X-ray Crystallographic Data

Crystallographic data for compounds **2** and **3** are summarized in **Table S2**. Suitable crystals for X-ray analysis of each of the above-mentioned compounds were placed on the end of a Cryoloop coated in NVH oil. The X-ray intensity data collection of **3** was carried out on a Bruker APEXII[19] with CCD area detector using graphite-monochromated Mo–K_α radiation (λ = 0.71073 Å) at 100.(2) K. The X-ray intensity data collection of **2** was carried out on Rigaku XtaLAB Synergy-I,[20] using HPC area detector (HyPix-3000HE), employing confocal multilayer optic-monochromated Mo-Kα radiation (λ=0.71073Å) at a temperature of 100K. Preliminary indexing was performed from a series of 0.5° rotation frames with exposures of 10 and 15 for **3** and **2**, respectively. In case of **3**, rotation frames were integrated using SAINT,[21] producing a listing of non-averaged F^2 and $\sigma(F^2)$ values which were then passed to the SHELXTL[22] program package for further processing and structure solution. The integration process for **2** was performed using CrysAlisPro[20, 23], producing a listing of unaveraged F^2 and $\sigma(F^2)$ values. The intensity data were corrected for Lorentz and polarization effects and for absorption using SADABS[24] or SCALE3 ABSPACK.[25] The structure was solved by direct methods - SHELXT.[26] Refinement was by full-matrix least squares based on F^2 using SHELXL-2018.[27] All reflections were used during refinement. Non-hydrogen atoms were refined anisotropically and hydrogen atoms were refined using a riding model. These results were checked using the IUCR's CheckCIF routine.

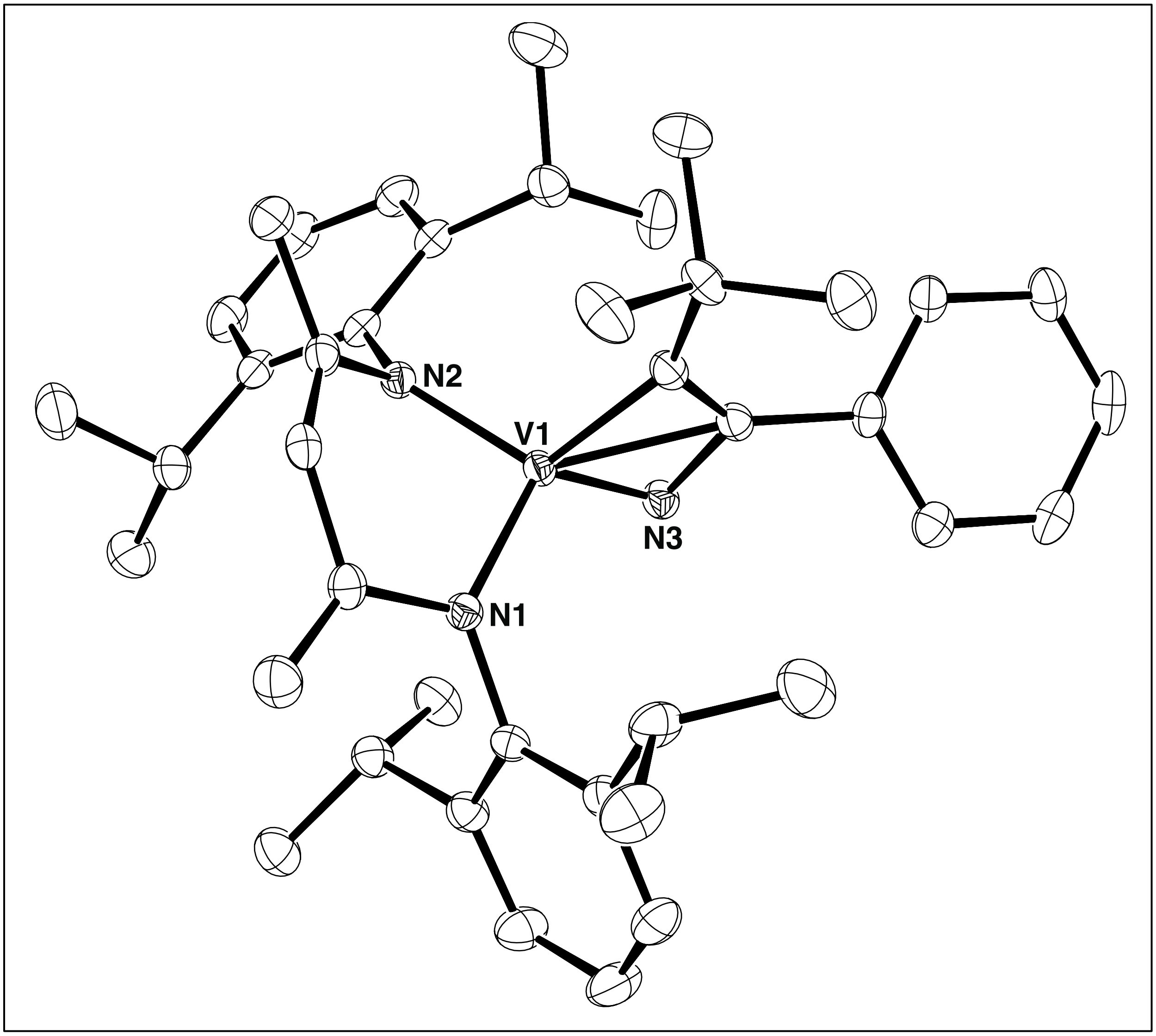

Figure S43: ORTEP drawing of **2** showing thermal ellipsoids with 50% probability level. Hydrogen atoms have been omitted for clarity

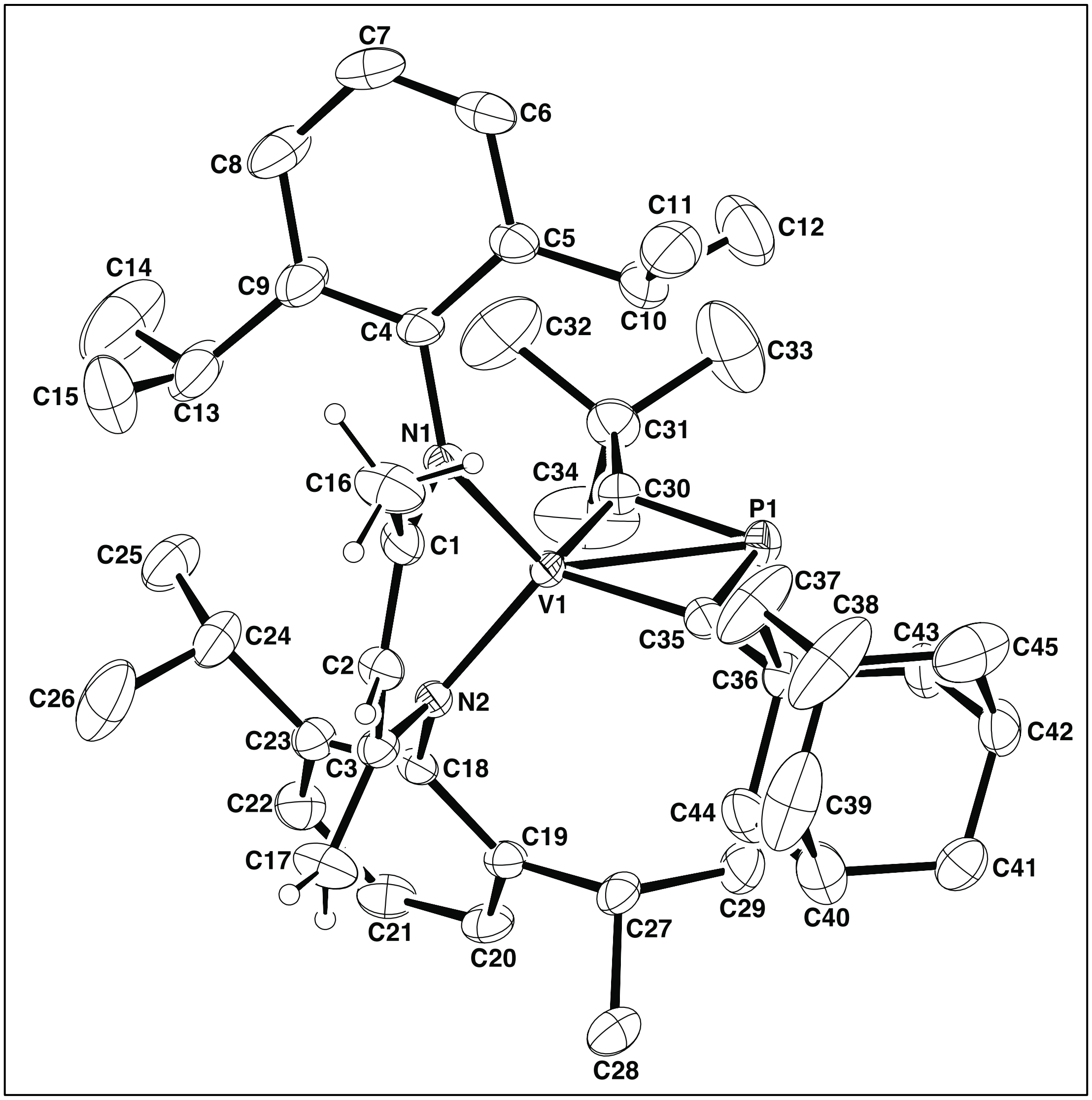

Figure S44: ORTEP drawing of **3** showing thermal ellipsoids with 50% probability level. Hydrogen atoms (except salient hydrogens of the ligand backbone) have been omitted for clarity

Table S2: Summary of structure determination of compounds **2** and **3**

Compound	**2**	**3**
CCDC Entry	2379015	2379016
Empirical formula	$C_{41}H_{55}N_3V$	$C_{45}H_{64}N_2PV$
Formula weight	640.82	714.89
Diffractometer	Rigaku XtaLAB Synergy–I (HyPix–3000HE)	Bruker APEXII
Temperature/K	100	100
Crystal system	monoclinic	monoclinic
Space group	$P2_1/n$	C2/c
a	21.2051(15)Å	37.668(3)Å
b	12.7630(2)Å	10.2592(10)Å
c	27.536(2)Å	20.923(2)Å
β	145.799(17)°	91.771(6)°
Volume	4188.9(11)Å^3	8081.7(13)Å^3
Z	4	8
d_{calc}	1.016 g/cm^3	1.175 g/cm^3
μ	0.264 mm^{-1}	0.317 mm^{-1}
F(000)	1380.0	3088.0
Crystal size, mm	0.5 × 0.27 × 0.07	0.16 × 0.14 × 0.04
2θ range for data collection	3.73 – 56.242°	3.896 – 55.1°
Index ranges	$-27 \leq h \leq 27, -16 \leq k \leq 16, -36 \leq l \leq 35$	$-48 \leq h \leq 48, -13 \leq k \leq 13, -27 \leq l \leq 27$
Reflections collected	64993	100472
Independent reflections	9922[R(int) = 0.0463]	9295[R(int) = 0.0596]
Data/restraints/parameters	9922/0/419	9295/234/508
Goodness-of-fit on F^2	1.099	1.036
Final R indexes [I≥2σ (I)]	$R_1 = 0.0418$, $wR_2 = 0.1070$	$R_1 = 0.0472$, $wR_2 = 0.1180$
Final R indexes [all data]	$R_1 = 0.0572$, $wR_2 = 0.1130$	$R_1 = 0.0696$, $wR_2 = 0.1308$
Largest diff. peak/hole	0.68/–0.72 eÅ^{-3}	0.79/–0.31 eÅ^{-3}

8. Appendix

8.1. Cartesian Coordinates of the Optimized Structures

==
=

2

==
=

N	0	0	0
C	0.98456651	0.89464293	-0.45478329
C	1.47957643	1.88736464	0.51169814
H	2.36208974	2.41142782	0.16809817
C	1.01900813	2.26093618	1.73331725
N	-0.06333968	1.63420924	2.36513343
C	-0.62490592	-0.88006246	-0.93611999
C	-1.8251243	-0.49418444	-1.56468958
C	-2.40681112	-1.35993047	-2.48628101
H	-3.32815512	-1.07900151	-2.97539372
C	-1.83336645	-2.58608353	-2.78413794
H	-2.30197226	-3.24525997	-3.50340026
C	-0.66575598	-2.96503581	-2.14719698
H	-0.22527173	-3.92711294	-2.3725034
C	-0.04653964	-2.13111782	-1.21845427
C	1.22392112	-2.61032161	-0.54772675

H	1.49386988	-1.87093896	0.20096656
C	0.9990367	-3.94386522	0.17095205
H	0.78489464	-4.74702897	-0.5369058
H	0.15964665	-3.88061227	0.86378482
H	1.88855064	-4.22874481	0.7346843
C	2.38889618	-2.70966256	-1.53840298
H	2.59782217	-1.73841434	-1.98518816
H	2.16695402	-3.41833285	-2.33934925
H	3.29233428	-3.05404959	-1.02959363
C	-2.47901363	0.84055097	-1.26958401
H	-2.18527891	1.11799084	-0.25738583
C	-1.9477045	1.91864331	-2.22468433
H	-2.15951577	1.63771949	-3.25926886
H	-0.87117779	2.03901688	-2.12073769
H	-2.42701924	2.87926245	-2.03099917
C	-4.00827546	0.77326574	-1.3218631
H	-4.39392442	-0.04970099	-0.71862784
H	-4.36932748	0.64098364	-2.34339398
H	-4.43891973	1.70101528	-0.94848511
C	1.48903799	0.8740094	-1.70684814
H	1.13301471	0.17523443	-2.44706281
H	2.24000123	1.59206535	-1.99926788

C	1.73701877	3.31927326	2.52511654
H	1.03497712	4.03292398	2.9558123
H	2.45425548	3.85603457	1.90723451
H	2.27221975	2.85723882	3.35832009
C	-0.91013191	2.36564872	3.24434852
C	-0.85550962	2.13285833	4.63062929
C	-1.70738077	2.85270774	5.4652078
H	-1.67464711	2.68047931	6.53289922
C	-2.57949817	3.79917705	4.95527696
H	-3.22772874	4.35703428	5.61848093
C	-2.61871539	4.02737522	3.58846763
H	-3.30703718	4.76357205	3.19769281
C	-1.8055897	3.31332492	2.71309502
C	-1.90559368	3.56252117	1.22042955
H	-1.44465165	2.71434194	0.71620233
C	-3.36447655	3.65342828	0.76475543
H	-3.41562205	3.71131524	-0.3214158
H	-3.85339853	4.54650995	1.15783317
H	-3.93369962	2.78500401	1.09591992
C	-1.13461275	4.82004333	0.79950898
H	-0.07389095	4.73295074	1.02664648
H	-1.52404263	5.70123251	1.31442134

H	-1.23310717	4.9792111	-0.27607392
C	0.12950233	1.16398963	5.25013595
H	0.67677856	0.69228413	4.43906847
C	1.15045292	1.89816376	6.12739413
H	1.90671016	1.20355888	6.49766806
H	0.66769696	2.36140017	6.99034859
H	1.65368017	2.68763681	5.56873834
C	-0.57626611	0.05820168	6.03852542
H	-1.31740729	-0.44372774	5.41917235
H	-1.08714947	0.45888283	6.91662839
H	0.14384957	-0.68651359	6.38355282
V	-0.05949422	-0.18911892	1.88520164
C	1.34433629	-1.23133227	2.70248447
N	-1.0620223	-1.28128442	2.67846499
C	0.21660514	-1.8781897	3.13138879
C	2.88083485	-1.21113214	2.80428884
C	3.40070008	-2.3735385	3.67040177
H	3.23199977	-3.34063898	3.18320026
H	4.47661232	-2.2814991	3.85761595
H	2.89758459	-2.40202953	4.64362925
C	3.53641465	-1.33750424	1.41654059
H	4.62978217	-1.34710726	1.49113078

H	3.23173045	-2.26258689	0.91404962
H	3.25732116	-0.50104808	0.76572833
C	3.38364113	0.09971071	3.43712126
H	4.47316929	0.09345337	3.55543313
H	3.12373666	0.9665895	2.81899639
H	2.94485455	0.25802233	4.42884322
C	0.05525523	-3.12868661	4.01559672
C	-0.93241352	-3.18549268	5.01340174
C	0.89152569	-4.24525033	3.84746417
C	-1.07869621	-4.31717355	5.81385395
C	0.74573316	-5.37721599	4.64760305
C	-0.24020456	-5.41856433	5.63473549
H	-1.58988347	-2.33438897	5.16111817
H	1.66160691	-4.22359218	3.0826307
H	-1.84943577	-4.3383427	6.57904639
H	1.40388702	-6.22861058	4.49938757
H	-0.35398771	-6.30040854	6.25827453

===
=

3

===
=

N	-0.26026637	0.08060384	-0.1423729
C	0.83818616	0.89963519	-0.51733608
C	1.27632299	1.89433624	0.43812193
H	2.1563495	2.43765167	0.10504712
C	0.75764778	2.29164854	1.6144635
N	-0.37006824	1.65363521	2.18553725
C	-0.7027946	-0.90319779	-1.11334457
C	-1.76457189	-0.59220669	-1.96211848
C	-2.16448487	-1.54255696	-2.89874939
H	-2.97274561	-1.32274027	-3.58699744
C	-1.54490359	-2.78128337	-2.95781727
H	-1.86828397	-3.51462952	-3.68812482
C	-0.51812926	-3.08490234	-2.07745976
H	-0.04594155	-4.05983787	-2.12909061
C	-0.08765157	-2.15131429	-1.13752703
C	1.04403974	-2.49366608	-0.19934876
H	1.14690668	-1.68174084	0.53017068
C	0.75349321	-3.7775224	0.58529129
H	0.62273837	-4.63270896	-0.07742536
H	-0.15487635	-3.65031704	1.17659635
H	1.58017226	-3.98620345	1.26552591
C	2.35282734	-2.61994476	-0.98983774

H	2.55304769	-1.69274817	-1.52775705
H	2.29183897	-3.43578438	-1.71105171
H	3.18675639	-2.81716023	-0.31629459
C	-2.45551575	0.75002055	-1.89523608
H	-2.23273942	1.20531038	-0.92295559
C	-1.90665963	1.6760297	-2.99008555
H	-2.07841192	1.24668485	-3.97792299
H	-0.83380992	1.81893402	-2.853038
H	-2.3957738	2.64928446	-2.93998651
C	-3.97797371	0.60419239	-2.01654757
H	-4.34069683	-0.1388622	-1.30561024
H	-4.26950543	0.29755002	-3.02140005
H	-4.46239048	1.55561767	-1.80009656
C	1.44676195	0.76399677	-1.71253302
H	1.1433615	0.02939098	-2.44218007
H	2.28744192	1.38770895	-1.97614538
C	1.38097765	3.38668322	2.41451943
H	0.63814763	4.12815217	2.70761693
H	2.17079886	3.87691701	1.84770542
H	1.81526907	2.96841792	3.32331778
C	-1.23367951	2.43854854	3.05694235
C	-1.15057131	2.27506025	4.43830705

C	-2.06208929	2.95492107	5.24430837
H	-2.02510124	2.83505153	6.32157862
C	-3.01264119	3.79499157	4.68875498
H	-3.71181346	4.32259356	5.32755157
C	-3.06154825	3.96646147	3.31425666
H	-3.79979064	4.63911823	2.89228549
C	-2.18620339	3.27847573	2.47701788
C	-2.29424494	3.46741228	0.97944652
H	-1.65985126	2.72694462	0.47869507
C	-3.73797307	3.26072464	0.50478902
H	-3.78300625	3.33809809	-0.58120368
H	-4.40616745	4.01213417	0.92604652
H	-4.09330455	2.27454412	0.80370027
C	-1.80437782	4.8651036	0.57498868
H	-0.76026829	4.99663483	0.85782074
H	-2.39642782	5.63935235	1.06447051
H	-1.88892286	4.99116648	-0.50486382
C	-0.07181318	1.44173243	5.09097612
H	0.59161128	1.06036697	4.30613351
C	0.76000058	2.31006043	6.04729551
H	1.61421806	1.74253778	6.41702319
H	0.16432053	2.63126182	6.90197274

H	1.13000738	3.19603596	5.53126303
C	-0.65199148	0.23416768	5.83623298
H	-0.94908153	-0.516731	5.09762564
H	-1.51536869	0.50778393	6.44116923
H	0.10570589	-0.20963825	6.48272917
V	-0.09139893	-0.15864317	1.70375406
C	1.16729685	-1.1566258	2.94648439
C	-1.45525895	-1.34485037	2.55616837
P	-0.20241713	-2.0267533	3.53219277
C	4.21000651	-3.04494269	4.52175879
C	4.53656773	-2.03520257	5.62817872
C	4.39035262	-0.61451367	5.07165936
C	2.95152053	-0.39712441	4.58364277
C	2.59362528	-1.40945479	3.47939134
C	2.76782701	-2.83104614	4.0455311
C	5.36150058	-0.41347022	3.90238898
C	5.17505838	-2.85205642	3.34630403
C	3.59245069	-1.21514158	2.31797392
C	5.03130808	-1.42692581	2.79991761
C	-2.9467611	-1.69514331	2.68339921
C	-3.16716396	-3.21226227	2.57076938
C	-3.53587037	-1.19212816	4.01094274

C	-3.68278838	-0.99668608	1.52015631
H	4.31529542	-4.0650756	4.91435742
H	5.55830784	-2.19223599	5.98911323
H	3.85346441	-2.17470439	6.47257863
H	4.61274634	0.11254349	5.86411002
H	2.2600943	-0.50792631	5.42368146
H	2.8525896	0.61841133	4.18733847
H	2.07652281	-2.97543668	4.88396665
H	2.51755513	-3.56679668	3.27410508
H	6.39246489	-0.55388283	4.24317211
H	5.26838937	0.60553654	3.512686
H	4.94772874	-3.57627763	2.55668453
H	6.20483299	-3.02506376	3.67676244
H	3.47327099	-0.20975411	1.90214409
H	3.36555123	-1.9351671	1.52479044
H	5.72153247	-1.28929158	1.95711364
H	-4.23005685	-3.45697271	2.61629333
H	-2.76380057	-3.58014801	1.62627544
H	-2.6558169	-3.72803001	3.38590505
H	-3.05613693	-1.69467679	4.85216909
H	-3.37721074	-0.11781568	4.10927694
H	-4.60842694	-1.3913848	4.05558798

H	-3.2797931	-1.34598129	0.56867871
H	-4.75265868	-1.20756685	1.54806223
H	-3.53681847	0.0826792	1.57806834

===
=

4

===
=

V	-0.04801538	-0.37538856	0.71437474
N	-1.4775621	0.63657989	1.43203499
N	1.15102361	-0.28796688	2.21263888
C	-1.92080255	0.01905234	2.63575279
C	-3.23529211	-0.01726693	2.98286322
C	-0.89266183	-0.56179245	3.5002099
C	0.47424468	-0.66488852	3.36641423
C	1.28498366	-1.2348473	4.49829841
C	0.68023931	-0.18419024	-0.95248191
C	-0.46273396	-2.10304153	0.17141031
C	0.17131932	-1.53437252	-0.96410542
C	2.57615392	-0.16534011	2.18066992
C	3.37248298	-1.28546106	1.83773358
C	4.76423481	-1.14563761	1.78946176

C	5.3795042	0.06707212	2.06728594
C	4.59096139	1.1658436	2.39294474
C	3.19542896	1.08072303	2.45430182
C	-2.31831954	1.61317186	0.82035009
C	-3.07686287	1.27235488	-0.32631797
C	-3.86770034	2.25596721	-0.93201627
C	-3.94666678	3.54115966	-0.41246352
C	-3.23199193	3.85618928	0.73990484
C	-2.41373866	2.91770287	1.37418369
C	1.41091188	0.54647841	-2.0421933
C	2.89783185	0.64370081	-1.63475467
C	0.81700135	1.96426685	-2.15282529
C	1.29945645	-0.1680968	-3.39729624
C	-1.172195	-3.42288475	0.29514309
C	-2.21921402	-3.30458137	1.41673015
C	-1.86888211	-3.80100938	-1.02405266
C	-0.17069622	-4.53409667	0.66517965
C	2.44763358	2.36956786	2.74859143
C	1.58240995	2.3312604	4.0134946
C	1.69035546	2.86243752	1.51012701
C	2.78376554	-2.64287825	1.49994376
C	3.09973849	-3.02037299	0.04572177

C	3.29013147	-3.73802657	2.44986317
C	-1.67627492	3.32236461	2.63653342
C	-1.07775552	4.73216535	2.52835039
C	-2.58963378	3.25072125	3.87149046
C	-3.14028331	-0.1366485	-0.88436336
C	-2.70771972	-0.19335937	-2.3538277
C	-4.54416047	-0.73031689	-0.695167
H	-3.54155889	-0.47782222	3.91985361
H	-3.99363692	0.45903336	2.36541013
H	-1.29562293	-0.97080694	4.42831406
H	2.18000867	-0.62463782	4.68254928
H	0.69048303	-1.28328999	5.4176899
H	1.63438528	-2.2479763	4.25167287
H	0.22452088	-2.10552526	-1.89848597
H	5.37664659	-2.0096679	1.52686756
H	6.46549731	0.16003792	2.02500726
H	5.06384877	2.12791778	2.60034602
H	-4.44848206	1.99891818	-1.81939638
H	-4.56956788	4.29474341	-0.89634885
H	-3.30640206	4.86240589	1.15309773
H	3.44618601	1.26960928	-2.35620164
H	3.0051223	1.07926803	-0.63265227

H	3.36691751	-0.34990408	-1.61411806
H	0.89462509	2.49209522	-1.19380256
H	1.35849263	2.54345023	-2.91661328
H	-0.24605467	1.93054023	-2.42934984
H	1.8317731	0.40846372	-4.16866604
H	1.75380725	-1.16969419	-3.35974987
H	0.25070207	-0.26942395	-3.71301368
H	-2.74076536	-4.26464523	1.55216797
H	-1.73486802	-3.0268269	2.36339428
H	-2.96298747	-2.52934629	1.19049672
H	-2.41609742	-4.74793021	-0.90023813
H	-2.58691014	-3.02700923	-1.3245382
H	-1.14287893	-3.93904906	-1.83910546
H	0.32241378	-4.31505869	1.62157433
H	-0.70136036	-5.49388719	0.76434463
H	0.59961099	-4.64444904	-0.11002949
H	3.2380978	3.11604188	2.94235264
H	0.71611915	1.6674397	3.91185071
H	1.20956511	3.34092308	4.24040287
H	2.17615081	1.99203148	4.87507964
H	1.30396277	3.87528301	1.67879028
H	0.83944864	2.2113642	1.26826544

H	2.36299702	2.89443679	0.6420308
H	1.69085528	-2.57006185	1.60178345
H	2.69150595	-4.01216406	-0.19399577
H	4.18650288	-3.05599496	-0.12271687
H	2.67213192	-2.28941245	-0.65146672
H	3.14254697	-3.4658954	3.50352404
H	4.36578547	-3.92055883	2.30620419
H	2.76399388	-4.68461327	2.2571831
H	-0.85858565	2.60258784	2.78016456
H	-0.55814898	4.88282162	1.57283665
H	-0.36725575	4.90654812	3.34921568
H	-1.85849028	5.50326834	2.61194101
H	-2.02606346	3.53421259	4.773315
H	-2.98920077	2.23904646	4.01145787
H	-3.43382339	3.94873464	3.76058751
H	-2.446216	-0.76039447	-0.30206918
H	-2.70253729	-1.23094865	-2.719021
H	-1.69877189	0.21958602	-2.47771592
H	-3.39302547	0.3806556	-2.99542141
H	-5.29935622	-0.14846238	-1.24489482
H	-4.8258198	-0.73674389	0.36666073
H	-4.58086659	-1.76557846	-1.06666949

9. References

1. D. Adhikari, F. Basuli, J. H. Orlando, X. Gao, J. C. Huffman, M. Pink and D. J. Mindiola, Zwitterionic and Cationic Titanium and Vanadium Complexes Having Terminal M−C Multiple Bonds. The Role of the β-Diketiminate Ligand in Formation of Charge-Separated Species, *Organometallics*, 2009, **28**, 4115-4125.

2. R. L. Gdula, M. J. Johnson and N. W. Ockwig, Nitrogen-atom exchange mediated by nitrido complexes of molybdenum, *Inorg. Chem.*, 2005, **44**, 9140-9142.

3. R. G. P. a. Y. Weitao, *Density Functional Theory of Atoms and Molecules*, Oxford University Press, 1989.

4. G. t. Te Velde, F. M. Bickelhaupt, E. J. Baerends, C. Fonseca Guerra, S. J. van Gisbergen, J. G. Snijders and T. Ziegler, Chemistry with ADF, *J. Comput. Chem.*, 2001, **22**, 931-967.

5. J. P. Perdew, K. Burke and M. Ernzerhof, Generalized gradient approximation made simple, *Phys. Rev. Lett.*, 1996, **77**, 3865.

6. S. Grimme, J. Antony, S. Ehrlich and H. Krieg, A consistent and accurate ab initio parametrization of density functional dispersion correction (DFT-D) for the 94 elements H-Pu, *J. Chem. Phys.*, 2010, **132**.

7. S. Grimme, S. Ehrlich and L. Goerigk, Effect of the damping function in dispersion corrected density functional theory, *J. Comput. Chem.*, 2011, **32**, 1456-1465.

8. E. Van Lenthe and E. J. Baerends, Optimized Slater- type basis sets for the elements 1–118, *J. Comput. Chem.*, 2003, **24**, 1142-1156.

9. J. I. Rodríguez, R. F. Bader, P. W. Ayers, C. Michel, A. W. Götz and C. Bo, A high performance grid-based algorithm for computing QTAIM properties, *Chem. Phys. Lett.*, 2009, **472**, 149-152.

10. J. I. Rodríguez, An efficient method for computing the QTAIM topology of a scalar field: The electron density case, *J. Comput. Chem.*, 2013, **34**, 681-686.

11. A. D. Becke and K. E. Edgecombe, A simple measure of electron localization in atomic and molecular systems, *J. Chem. Phys.*, 1990, **92**, 5397-5403.

12. J. C. Slater, *The self-consistent field for molecules and solids*, McGraw-Hill, 1974.

13. S. H. Vosko, L. Wilk and M. Nusair, Accurate spin-dependent electron liquid correlation energies for local spin density calculations: a critical analysis, *Can. J. Phys.*, 1980, **58**, 1200-1211.

14. A. D. Becke, Density-functional exchange-energy approximation with correct asymptotic behavior, *Phys. Rev. A*, 1988, **38**, 3098.

15. C. Lee, W. Yang and R. G. Parr, Development of the Colle-Salvetti correlation-energy formula into a functional of the electron density, *Phys. Rev. B*, 1988, **37**, 785.

16. Y. Zhao and D. G. Truhlar, A new local density functional for main-group thermochemistry, transition metal bonding, thermochemical kinetics, and noncovalent interactions, *J. Chem. Phys.*, 2006, **125**.

17. I. Mayer, Charge, bond order and valence in the AB initio SCF theory, *Chem. Phys. Lett.*, 1983, **97**, 270-274.

18. R. F. Nalewajski, J. Mrozek and A. Michalak, Two- electron valence indices from the Kohn- Sham orbitals, *Int. J. Quantum Chem.*, 1997, **61**, 589-601.

19. M. APEX3 2014.11-0: Bruker-AXS, Wisconsin, USA 2014.

20. R. C. CrysAlisPro 1.171.40.53: Rigaku Oxford Diffraction, Oxford, UK., 2019.

21. A. Bruker, Saint and SADABS, *Bruker AXS Inc., Madison, Wisconsin, USA*, 2009.

22. N. T. Shelxtl, 6.12, Bruker AXS Inc., Madison WI, USA, 2002, *Received: June*, 2009, **26**.

23. R. C. CrysAlisPro 1.171.40.89a: Rigaku Oxford Diffraction, Oxford, UK., 2020.

24. G. M. Sheldrick, SADABS, version 2.05 and version 2007/2; University of Göttingen: Göttingen, Germany, 2002, 2007, *Sheldrick, GM TWINABS, version*, 2007, **5**.

25. , SCALE3 ABSPACK v1.0.7: an Oxford Diffraction program, *Oxford Diffraction Ltd: Abingdon, UK*, 2005.

26. G. M. Sheldrick, SHELXT–Integrated space-group and crystal-structure determination, *Acta Crystallogr., Sect. A*, 2015, **71**, 3-8.

27. G. M. Sheldrick, Crystal structure refinement with SHELXL, *Acta Crystallogr., Sect. C,* 2015, **71**, 3-8.
